# Copper Coordination Chemistry of Sulfur Pendant Cyclen Derivatives: An Attempt to Hinder the Reductive-Induced Demetalation in ^64/67^Cu Radiopharmaceuticals

**DOI:** 10.1021/acs.inorgchem.1c01550

**Published:** 2021-07-19

**Authors:** Marianna Tosato, Marco Dalla Tiezza, Nóra V. May, Abdirisak Ahmed Isse, Sonia Nardella, Laura Orian, Marco Verona, Christian Vaccarin, André Alker, Helmut Mäcke, Paolo Pastore, Valerio Di Marco

**Affiliations:** † Department of Chemical Sciences, University of Padova, via Marzolo 1, 35131 Padova, Italy; ‡ Centre for Structural Science, Research Centre for Natural Sciences, Magyar tudósok Körútja 2, 1117 Budapest, Hungary; § Department of Pharmaceutical Sciences, University of Padova, via Marzolo 8, 35131 Padova, Italy; ∥ Roche Pharmaceutical Research and Early Development, Roche Innovation Center Basel F. Hoffmann-La Roche, Grenzacherstrasse 124, 4058 Basel, Switzerland; ⊥ Department of Nuclear Medicine, University Hospital Freiburg, Hugstetterstrasse 55, D-79106 Freiburg, Germany

## Abstract

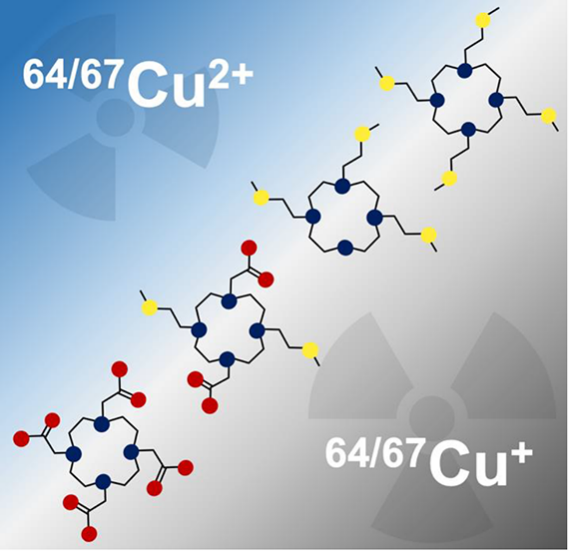

The Cu^2+^ complexes formed by a series of cyclen derivatives bearing sulfur pendant arms, 1,4,7,10-tetrakis[2-(methylsulfanyl)ethyl]-1,4,7,10-tetraazacyclododecane (DO4S), 1,4,7-tris[2-(methylsulfanyl)ethyl]-1,4,7,10-tetraazacyclododecane (DO3S), 1,4,7-tris[2-(methylsulfanyl)ethyl]-10-acetamido-1,4,7,10-tetraazacyclododecane (DO3SAm), and 1,7-bis[2-(methylsulfanyl)ethyl]-4,10-diacetic acid-1,4,7,10-tetraazacyclododecane (DO2A2S), were studied in aqueous solution at 25 °C from thermodynamic and structural points of view to evaluate their potential as chelators for copper radioisotopes. UV–vis spectrophotometric out-of-cell titrations under strongly acidic conditions, direct in-cell UV–vis titrations, potentiometric measurements at pH >4, and spectrophotometric Ag^+^–Cu^2+^ competition experiments were performed to evaluate the stoichiometry and stability constants of the Cu^2+^ complexes. A highly stable 1:1 metal-to-ligand complex (CuL) was found in solution at all pH values for all chelators, and for DO2A2S, protonated species were also detected under acidic conditions. The structures of the Cu^2+^ complexes in aqueous solution were investigated by UV–vis and electron paramagnetic resonance (EPR), and the results were supported by relativistic density functional theory (DFT) calculations. Isomers were detected that differed from their coordination modes. Crystals of [Cu(DO4S)(NO_3_)]·NO_3_ and [Cu(DO2A2S)] suitable for X-ray diffraction were obtained. Cyclic voltammetry (CV) experiments highlighted the remarkable stability of the copper complexes with reference to dissociation upon reduction from Cu^2+^ to Cu^+^ on the CV time scale. The Cu^+^ complexes were generated in situ by electrolysis and examined by NMR spectroscopy. DFT calculations gave further structural insights. These results demonstrate that the investigated sulfur-containing chelators are promising candidates for application in copper-based radiopharmaceuticals. In this connection, the high stability of both Cu^2+^ and Cu^+^ complexes can represent a key parameter for avoiding *in vivo* demetalation after bioinduced reduction to Cu^+^, often observed for other well-known chelators that can stabilize only Cu^2+^.

## Abstract 2

A series of cyclen derivatives bearing sulfur- or mixed-sulfur-carboxylated pendant arms were considered as chelators for copper radioisotopes in radiopharmaceuticals. Stability and structure of their Cu^2+^ and Cu^+^ complexes were studied in aqueous solution using a multimethodological approach. The stability of both Cu^2+^ and Cu^+^ complexes should prevent the reductive-based copper demetallation observed *in vivo* for other copper chelators.

## Introduction

A flourished number of researches have been conducted during the past decades to develop radiopharmaceuticals for noninvasive imaging and treatment of tumors. In particular, copper has received much interest because it possesses several radioisotopes (copper-60, copper-61, copper-62, copper-64, and copper-67) with half-life and emission properties suitable for diagnostic and therapeutic applications.^1−3^ Copper-64 (^64^Cu, *t*_1/2_ 12.7 h) is undoubtedly the most versatile because its unique decay profile, which combines electron capture (*I*_EC_ 43%), β^+^ (*I*_β^+^_ 18%, *E*_β^+^,max_ 655 keV) and β^–^ emission (*I*_β^–^_ 39%, *E*_β^–^,max_ 573 keV), makes it suitable for positron emission tomography (PET) imaging and, in principle, radiotherapy by using the same radiopharmaceutical.^4−6^ Furthermore, ^64^Cu can provide a matched PET imaging pair with the pure β^–^ emitter copper-67 (^67^Cu, *t*_1/2_ 61.9 h, β^*–*^ 100%, *E*_β^–^,max_ 141 keV).^7,8^ The theranostic approach of using both ^64^Cu and ^67^Cu can allow low-dose scouting scans to obtain dosimetry information, followed by higher dose therapy in the same patient, thus taking a major step toward personalized medicine.^9^

To obtain site-specific delivery of the emitted radiation, the radioisotopes must be firmly coordinated by a bifunctional chelator (BFC) appended to a tumor-targeting biomolecule (e.g., small molecule, peptide, or antibody) through a covalent linkage.^10−12^ If the radionuclide is released *in vivo* from the BFC, high background activity levels are detected, which limit target visualization under diagnostic imaging, and an unintended radiation burden occurs on healthy tissues.^13^ For these reasons, a suitable BFC for ^64/67^Cu should provide high thermodynamic stability and kinetic inertness to avoid possible transchelation and transmetalation reactions in biological media.^23^ Fast complexation under mild conditions is also crucial for allowing the use of heat- and pH-sensitive biovectors.^10,14,15^

A particular case of competitive reactions is represented by copper reduction from Cu^2+^ to Cu^+^, which can be promoted *in vivo* because of the presence of endogenous reductants. Cu^+^ possesses markedly different coordination preferences compared to Cu^2+^ and is much more labile to ligand exchange. Therefore, premature dissociation and release of ^64/67^Cu can occur.^16−18^ As such, it is important for a BFC selected for ^64/67^Cu to be able to firmly complex both Cu^2+^ and Cu^+^ or to stabilize Cu^2+^ to prevent reduction.^16,19−26^

**Figure 1:**
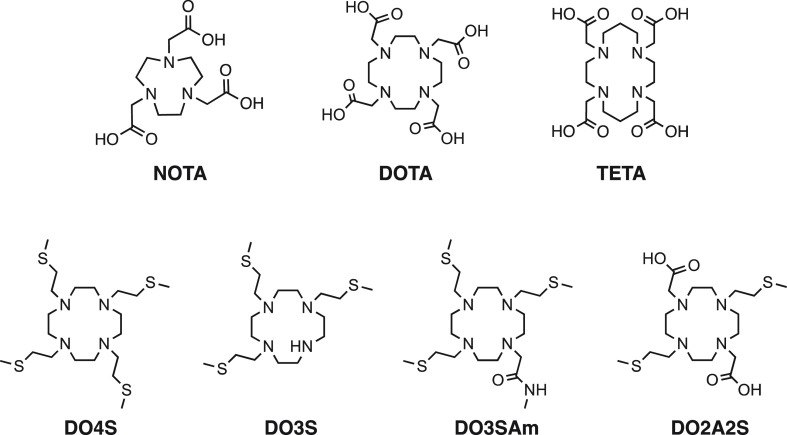
Select state-of-the-art copper chelators (NOTA, DOTA, and TETA) and ligands investigated in this work (DO4S, DO3S, DO3SAm, and DO2A2S).

Within the large number of acyclic and cyclic ligands that were investigated for copper radionuclides, the family of azamacrocyles provides a wide range of platforms useful for the design of progressively improved BCFs. For example, polyaminocarboxylate-based macrocycles, including 1,4,7,10-tetraazacyclododecane-1,4,7,10-tetraacetic acid (DOTA), 1,4,8,11-tetraazacyclotetradecane-1,4,8,11-tetraacetic acid (TETA; Figure 1), and their derivatives, form Cu^2+^ complexes with excellent thermodynamic stability but suffering from marked kinetic lability, which causes *in vivo* demetalation.^2,6,20,27,28^ To overcome this limit, constrained or reinforced polyaza chelators, such as dicarboxylic acid cross-bridged cyclen [4,10-bis(carboxymethyl-1,4,7,10-tetraazabicyclo[5.5.2]tetradecane, CB-DO2A], cyclam [4,11-bis(carboxymethyl)-1,4,8,11-tetrazabicyclo[6.6.2]hexadecane-4,11-diacetic acid, CB-TE2A], and other derivatives, were developed (Figure S1).^1,2,6,16,29−32^ The increased rigidity of the ligand backbone makes these complexes less prone to dissociation but also causes slow formation rates, thus needing harsh labeling conditions such as high temperature and prolonged reaction time. While still practicable for bioconjugates of some targeting vectors, these severe labeling conditions preclude the use of more thermosensitive biomolecules (e.g., antibodies). Besides the high kinetic inertness obtainable through structurally constrained derivatives, also 1,4,7-triazacyclononane-1,4,7-triacetic acid (NOTA; Figure 1) or its derivatives and sarcophagine chelators (Figure S1) have demonstrated remarkable inertness combined with mild labeling conditions.^33^

The quest for novel BFCs of ^64/67^Cu that combine high *in vivo* stability and kinetic inertness with quantitative and fast radiolabeling in mild conditions and no demetalation upon Cu^2+^/Cu^+^ reduction is still a significant challenge.^34^ With regard to the latter decomplexation pathway, only a few attempts have been made to develop BFCs able to securely bind both Cu^2+^ and Cu^+^.^25,35,36^ In light of this, we have hypothesized that the introduction of soft sulfur donor arms on a cyclen scaffold would stabilize both copper oxidation states, and we have chosen a small library of N-functionalized cyclen derivatives bearing sulfide pendant chains (Figure 1). These ligands have recently been considered in our previous works, where the formation of very stable complexes with soft metal ions (Ag^+^ and Cd^2+^) was observed.^37−39^

The cyclen and DOTA backbone has been modified by introducing an increasing number of sulfanyl arms, leading to 1,4,7,10-tetrakis[2-(methylsulfanyl)ethyl]-1,4,7,10-tetraazacyclododecane (DO4S), 1,4,7-tris[2-(methylsulfanyl)ethyl]-1,4,7,10-tetraazacyclododecane (DO3S), and 1,7-bis[2-(methylsulfanyl)ethyl]-4,10-diacetic acid-1,4,7,10-tetraazacyclododecane (DO2A2S).^38^ DO4S was designed as a model ligand in which all DOTA carboxylic groups have been substituted with sulfur donors. DO3S possesses a nonalkylated nitrogen that could be used as a reacting site to later covalently attach a biovector. To mimic the behavior of DO3S conjugated to a targeting molecule, 1,4,7-tris[2-(methylsulfanyl)ethyl]-10-acetamido-1,4,7,10-tetraazacyclododecane (DO3SAm) was considered as well. Finally, DO2A2S represents a hybrid ligand between DOTA and DO4S with two opposite sulfur atoms and two carboxylates.

To evaluate the potential of the proposed ligands as BFCs for ^64/67^Cu-based radiopharmaceuticals, we have investigated their Cu^2+^ and Cu^+^ complexes from thermodynamic and structural points of view. This study was performed with natural copper through UV–vis, electron paramagnetic resonance (EPR) and NMR spectroscopies, X-ray crystallography, and electrochemical methods [potentiometric titrations, cyclic voltammetry (CV), and electrolysis], and the results were supported by accurate relativistic density functional theory (DFT) calculations.

## Results and Discussion

### Protonation Properties of the Ligands

The basicity of different ionizable protons governs the competition between the metal ion of interest and the protons for the binding sites of the chelator during metal complexation.^40^ In our previous work, we have explored the acid–base properties of DO4S, DO3S, DO3SAm, and DO2A2S in aqueous NaNO_3_ (0.15 mol/L) at 25 °C using combined potentiometric and UV–vis spectrophotometric titrations.^37^ Despite DO4S, DO3S, and DO3SAm possessing four ionizable amino groups, only two acidity constants (p*K*_a3_ and p*K*_a4_) were accurately determined (Table S1).^37^ For DO2A2S, which contains six protonable sites (four amines and two carboxylates), the last three p*K*_a_ values were obtained (Table S1).^37^ The other acidity constants are very low (<2) because of the electrostatic repulsion between the positive charges resulting from the progressive protonation of the amino groups. For DO2A2S, protonations were unfavored also because of its capability to form intramolecular hydrogen bonds.

In the present work, other acidity constants, namely, p*K*_a2_ for DO4S, DO3S, and DO3SAm and p*K*_a3_ for DO2A2S, were determined using in-batch UV–vis spectrophotometric titrations at very acidic conditions (pH <2), where pH potentiometry cannot give reliable results. The p*K*_a2_ values for DO4S (1.9), DO3S (2.0), and DO3SAm (1.9) certainly belong to the amino groups, while the p*K*_a3_ value for DO2A2S (1.8) likely corresponds to the deprotonation of a carboxylate. The obtained values are summarized in Table S1, and the speciation diagrams are presented in Figures S2 and S3. The results are coherent with those usually observed for other cyclen derivatives.^41^

### Complexation Kinetics of Cupric Complexes

Preliminary data obtained on the complex formation between Cu^2+^ and the examined ligands demonstrated that these reactions can be remarkably slow. As the attainment of rigorous thermodynamic data requires solutions to be at equilibrium, time conditions for reaching equilibrium were explored as a function of pH and at room temperature before performing the thermodynamic measurements.

The UV–vis spectra and time course of the complexation reaction between Cu^2+^ and the investigated sulfide-bearing chelators are shown in Figures S4–S6. DOTA was also included for comparison purposes (Figures S7). At concentrations of ∼10^–4^ mol/L for both Cu^2+^ and the ligand, the complex formation was always found to be instantaneous (<10 s) at neutral pH, while at pH 4.8, it was complete (>99%) in a few seconds for DO2A2S and DOTA and within ∼1 h for DO4S, DO3S, and DO3SAm (Table S2). The reactions became progressively slower under increasingly acidic conditions, as resumed in Table S2: at pH 2.0, DOTA and DO2A2S reached the equilibrium in a few hours, while for the other ligands, the equilibrium was established only after ∼10 days. Other experiments were performed that showed the reaction rates increasing proportionally with the concentration of the reactants (Table S3).

The marked difference between the complex formation rates of the pure sulfide-bearing chelators and the carboxylate ones can be rationalized by analyzing the role that the acetate arms play in the complexation event. These negatively charged pendants can interact with the incoming Cu^2+^ ions, forming an out-of-cage intermediate, which is later transformed into an in-cage product (where the metal ion is coordinated by the nitrogen atoms and by the donor atoms of the pendants), so that the overall reaction can be accelerated by increasing the local concentration of the metal ion close to the ligand cavity.^42,43^ This ability has been indicated for DOTA, and it appears to be absent when all carboxylates are replaced by sulfanyl groups. If the pH decreases, protonated species become increasingly predominant (Figures S2 and S3). In these forms, the protons induce an electrostatic repulsion toward the Cu^2+^ ions and block access of the metal ion to the ligand cavity, progressively slowing complex formation.

### Solution Thermodynamics of Cupric Complexes

The slow equilibration at acidic pH (see above) and the high stability of the Cu^2+^ complexes formed by the examined ligands hampered determination of the equilibrium constants by conventional potentiometry. Therefore, UV–vis spectrophotometric out-of-cell titrations under strongly acidic conditions, direct in-cell UV–vis titrations, potentiometric titrations at pH >4, and spectrophotometric Ag^+^–Cu^2+^ competition experiments were performed.

**Figure 2:**
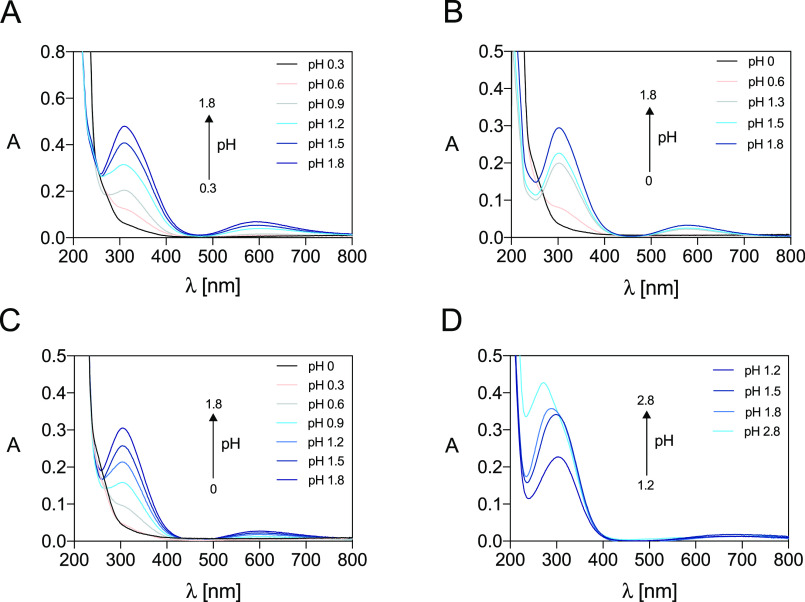
Select UV–vis spectra at pH <2 of the Cu^2+^ complexes formed by (A) DO4S (*C*_Cu_^2+^ = *C*_DO4S_ = 1.5 × 10^–4^ mol/L), (B) DO3S (*C*_Cu_^2+^ = *C*_DO3S_ = 1.0 × 10^–4^ mol/L), (C) DO3SAm (*C*_Cu_^2+^ = *C*_DO3SAm_ = 1.1 × 10^–4^ mol/L), and (D) DO2A2S (*C*_Cu_^2+^ = *C*_DO2A2S_ = 0.9 × 10^–4^ mol/L) at *I* = 0.15 mol/L NaCl (for solutions at pH >0.8) and *T* = 25.0 °C.

**Figure 3:**
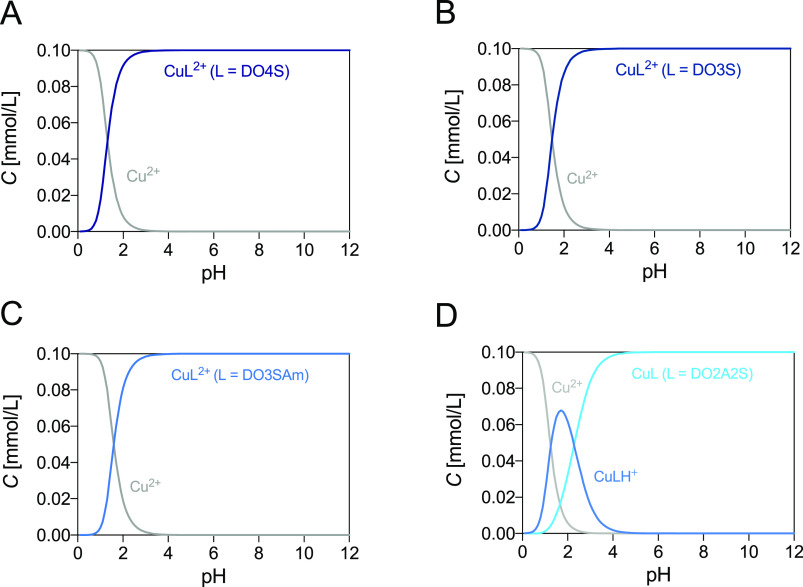
Distribution diagrams of (A) Cu^2+^-DO4S, (B) Cu^2+^-DO3S, (C) Cu^2+^-DO3SAm, and (D) Cu^2+^-DO2A2S. The plots were calculated from the overall stability constants reported in Tables 1 and S1 at *C*_Cu_^2+^ = *C*_L_ = 1.0 × 10^–4^ mol/L.

Figures 2 and S8 report the electronic spectra of solutions containing Cu^2+^-DO4S, Cu^2+^-DO3S, Cu^2+^-DO3SAm, and Cu^2+^-DO2A2S at equilibrium at pH <2 and >2, respectively, while the spectroscopic data are summarized in Table S4 (the spectra for the free ligands were obtained in our previous work^37^). The marked absorbance variations at pH <2 can be interpreted by the complex formation. At pH larger than ∼2, only very minor changes were detected in the spectra of Cu^2+^-DO4S, Cu^2+^-DO3S, and Cu^2+^-DO3SAm, suggesting that the speciation does not change in the investigated pH range (2–11). UV–vis titrations performed at different metal-to-ligand molar ratios demonstrated that only a 1:1 metal-to-ligand complex exists, as deduced from the sharp inflection point at ca. 1:1 molar ratio in the titration curves (Figure S9). The formation of only one Cu^2+^ complex in the pH range 4–11 was indicated also by potentiometric titrations. According to both spectrophotometric and potentiometric data, this complex is CuL^2+^, where L denotes the completely deprotonated ligand. For Cu^2+^-DO2A2S, formation of the deprotonated 1:1 metal-to-ligand complex (CuL) was also confirmed, but an additional species, CuLH^+^, was detected at pH below ∼4. The overall stability constants determined are given in Table 1, together with literature values for DOTA, and the corresponding distribution diagrams are shown in Figure 3.

**Table 1:** Overall Stability Constants (logβ) of the Cu^2+^ Complexes Formed by DO4S, DO3S, DO3SAm, and DO2A2S at *I* = 0.15 mol/L NaCl and *T* = 25 °C^a^

ligand	equilibrium reaction^b^	logβ
DO4S	Cu^2+^ + L ⇋ CuL^2+^	19.8 ± 0.1^c^
		19.6 ± 0.4^d^
DO3S	Cu^2+^ + L ⇋ CuL^2+^	20.34 ± 0.06^c^
		20.10 ± 0.08^d^
DO3SAm	Cu^2+^ + L ⇋ CuL^2+^	19.8 ± 0.2^c^
		19.7 ± 0.2^d^
DO2A2S	Cu^2+^ + H^+^ + L^2–^ ⇋ CuHL^+^	24.22 ± 0.09^c^
	Cu^2+^ + L^2–^ ⇋ CuL	22.0 ± 0.3^d^
		21.9 ± 0.2^c^
DOTA	Cu^2+^ + 2H^+^ + L^4–^ ⇋ CuH_2_L	30.8^e^
	Cu^2+^ + H^+^ + L^4–^ ⇋ CuHL^–^	26.60^e^
	Cu^2+^ + L^4–^ ⇋ CuL^2–^	22.30^e^

a The literature data for DOTA are reported for comparison.

b L denotes the ligand in its totally deprotonated form.

c Obtained by UV–vis spectrophotometric titrations.

d Obtained by Ag^+^–Cu^2+^ competition (no ionic strength control).

e From ref (44).

Competition Ag^+^–Cu^2+^ measurements were also performed to determine the Cu^2+^-ligand stability constants because the constants of the Ag^+^-ligand complexes are known.^38^ The electronic spectra of the preformed Ag^+^ complex with DO4S, DO3S, DO3SAm, and DO2A2S immediately after the addition of 0.2–4 equiv of Cu^2+^ and at equilibrium are shown in Figure S10. Figures S11–S14 reflect the changes in the spectra over time for each ligand, indicating the slow kinetics of the transmetalation reactions at room temperature. For this reason, the solutions containing Ag^+^, Cu^2+^, and the ligand were forced to equilibrium through heating. The increase of the absorption at the characteristic wavelength of the Cu^2+^ complexes clearly reflects their formation (Figure S15). It is worth noting that reaction intermediates can be detected in some cases if the UV–vis spectra at the reaction start and at equilibrium are compared with those obtained during the reaction course (e.g., Figure S11). The stability constants calculated by competitive titrations (Table 1) agree well with those obtained from UV–vis spectrophotometric measurements.

To gain insight into the *in vivo* stability of the cupric complexes and to compare the stability of the Cu^2+^ complexes formed by different ligands, the pCu^2+^ (pCu^2+^ = −log [Cu^2+^]_free_) was computed because this parameter takes into account the influence of ligand basicity and metal-ion hydrolysis: higher pCu^2+^ values denote more stable complexes under the specified conditions.^45^ The pCu^2+^ values of the investigated sulfide-bearing ligands and other important ^64/67^Cu^2+^ chelators, at various pH values, are listed in Table 2 (the thermodynamic stability of other radiopharmaceutically relevant Cu^2+^-chelator complexes can be found in the literature).^46^ The obtained results revealed that the investigated ligands form very stable Cu^2+^ complexes, with a pCu^2+^ value higher or comparable to those of the well-known ^64/67^Cu^2+^ chelators NOTA, DOTA, and TETA. Among those, DO2A2S forms the most stable complexes. Its higher stability compared to those of DO4S, DO3S, and DO3SAm can be attributed to the preference of Cu^2+^ to hard carboxylic donors rather than to soft sulfur ones. Compared to DOTA, the extra stability of the cupric complexes formed by DO2A2S should be related to the lower basicity of this ligand, which makes it a better complexing agent for Cu^2+^. It is also worth noting that the comparable stabilities of DO4S, DO3S, and DO3SAm indicate that the Cu^2+^ complexation properties are preserved upon the loss of one sulfide arm and N-alkylation of the nitrogen atom.

**Table 2:** pCu^2+^ Values for the Cupric Complexes Formed by DO4S, DO3S, DO3SAm, DO2A2S, and Select State-of-the-Art ^64/67^Cu^2+^ Ligands^a^

	pCu^2+^		
ligand	pH 4.0	pH 6.0	pH 7.4
DO4S	9.3	11.3	17.7
DO3S	8.9	10.9	17.5
DO3SAm	8.5	10.5	17.2
DO2A2S	10.1	12.5	19.4
DOTA	7.6	9.8	17.4
NOTA	10.9	13.0	18.2
TETA	7.3	9.6	16.2
Cyc4Me	7.3	11.3	14.1

a pCu^2+^ calculated at *C*_Cu_^2+^ = 10^–6^ mol/L and *C*_L_ = 10^–5^ mol/L using the constants of Tables 1 and S1 or taken from refs (44) and (48).

### Structural Investigation of the Cupric Complexes

The UV–vis absorption spectra of the Cu^2+^ complexes with DO4S, DO3S, and DO3SAm (Figures 2 and S8) were examined also to obtain structural information. Spectra display a strongly intense UV band (ε ≈ 3.6 × 10^3^ L/cm·mol; Table S4) centered at 309, 303, and 304 nm, respectively. Bosnich et al. have assigned the intense band in the 350 nm region in the spectra of square-planar, square-pyramidal, and tetrahedral amine–thioether donor arrays to a sulfur-to-Cu^2+^ ligand-to-metal charge-transfer transition.^47^ Therefore, the absorption at around 300 nm for the investigated Cu^2+^ complexes can be attributed to the same transition. A broadband above 500 nm (Figure S16) was also found in all solutions (ε ≈ 4 × 10^2^ L/cm·mol; Table S4), characteristic of the d–d orbital transition of the Cu^2+^ ion.

The involvement of the sulfur pendants in the Cu^2+^ coordination sphere is indicated also when the spectra of Figures 2 and S8 are compared to those of Cu^2+^-cyclen and Cu^2+^-1,4,7,10-tetra-*n*-butyl-1,4,7,10-tetraazacyclododecane (DOT-*n*-Bu; Figure S17), where DOT-*n*-Bu is the *tert*-butylated analogue of DO4S, which was considered to compare the electronic effect of secondary (cyclen) and tertiary (DOT-*n*-Bu) amines.^37^ The UV absorption peak of Cu^2+^-DOT-*n*-Bu is red-shifted with respect to that of Cu^2+^-cyclen, indicating that replacement of the Cu^2+^-coordinating secondary amines with tertiary ones has a role in the observed spectral changes. In turn, peaks of Cu^2+^-DO4S, Cu^2+^-DO3S, and Cu^2+^-DO3SAm are red-shifted with respect to that of Cu^2+^-DOT-*n*-Bu, so that a different coordination mode is suggested when sulfanyl arms replace *tert*-butyl ones; i.e., one or more sulfur atoms should be involved in the metal binding. Conversely, the visible bands attributed to the d–d transition (above 500 nm) are much more similar for all ligands.^41,49,50^ The extinction coefficients in the visible region are remarkably high, which can be explained by the so-called intensity-stealing or intensity-borrowing of a neighboring higher-energy transition. A strongly distorted arrangement is thus suggested.^51^ According to these results, the coordination sphere around the Cu^2+^ center can be depicted as either a distorted square pyramid or a distorted octahedron.^49^

The involvement of sulfur in the Cu^2+^ coordination can be deduced also if the pCu^2+^ for Cu^2+^-1,4,7,10-tetramethyl-1,4,7,10-tetrazacyclododecane (Cyc4Me) is compared to that for Cu^2+^-DO4S (Table 2) because the former contains tertiary amines but no sulfur donors: the Cu^2+^ complex formed by DO4S is more stable than that formed by Cyc4Me. A DFT calculation was performed to indicate whether this difference can be explained only by the electronic effects of the nitrogen atoms. The Gibbs free energies in water (Δ*G*_water_) of the two complexes were compared, supposing that both ligands bind the metal ion through all nitrogen atoms and no sulfur is involved for DO4S. The results (Table S5) show that the Cu^2+^ complex of DO4S is less stable than that of Cyc4Me by 3.3 kcal/mol. Because the experimental result was opposite, the coordinating role of sulfur(s) is further supported.

**Figure 4:**
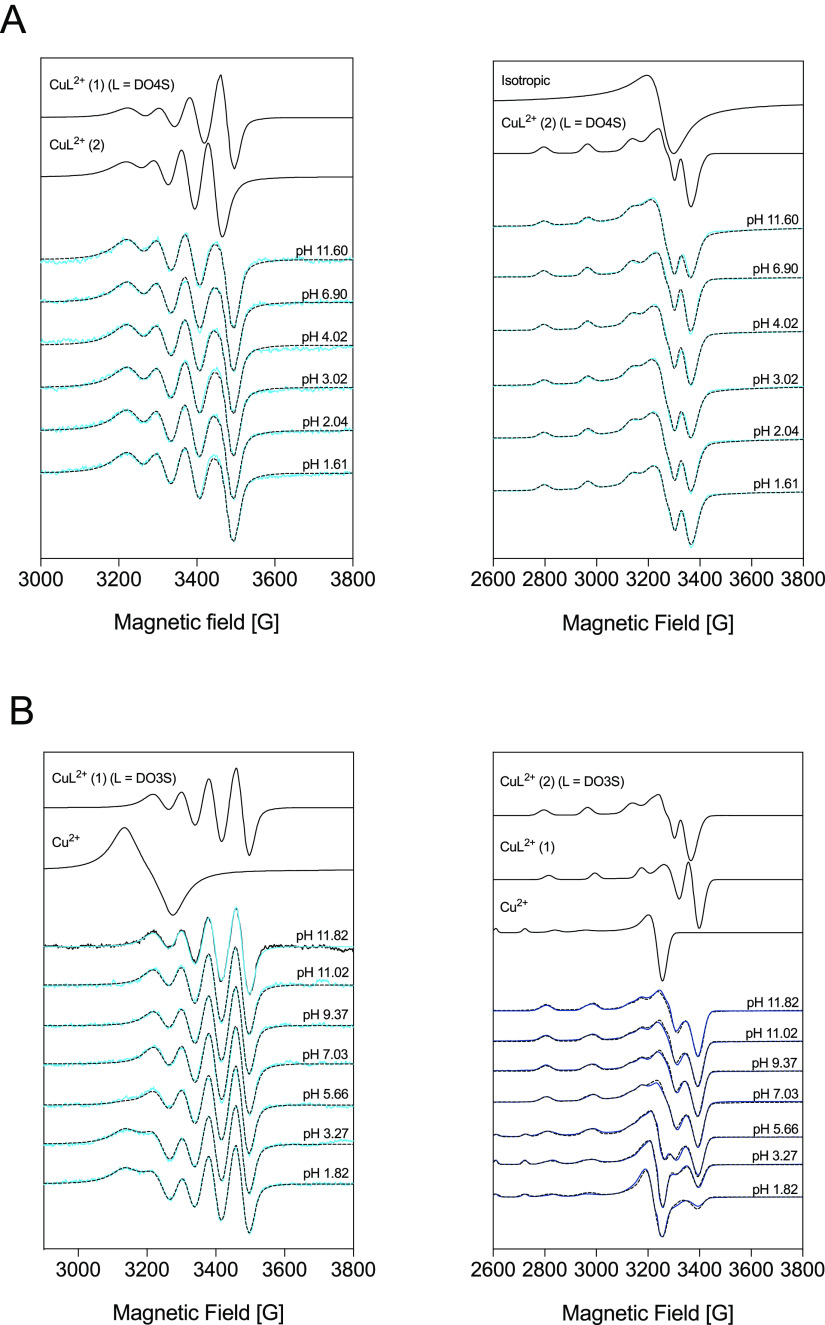
Measured (solid lines) and simulated (dotted lines) EPR spectra for solutions containing Cu^2+^ and (A) DO4S (*C*_Cu_^2+^ = 1.0 × 10^–3^ mol/L; *C*_DO4S_ = 1.3 × 10^–3^ mol/L) and (B) DO3S (*C*_Cu_^2+^ = 1.0 × 10^–3^ mol/L; *C*_DO3S_ = 1.1 × 10^–3^ mol/L) at room temperature (left) and 77 K (right). The component spectra obtained from the simulation are shown in the upper part.

To gain additional structural information, the cupric complexes of DO4S and DO3S were studied using EPR spectroscopy. The experimental EPR spectra are presented in Figure 4, together with the simulated ones using the parameters summarized in Table 3.

**Table 3:** EPR Parameters of the Components Obtained by the Simulation of Room Temperature (Isotropic Parameters) and 77 K (Anisotropic Parameters) Spectra Measured in Solutions Containing Cu^2+^-DO4S, Cu^2+^-DO3S, and Cu^2+^-DO2A2S and Suggested Coordination^a^

	isotropic parameters^b^		anisotropic parameters^c^					
	*g*_0_	*A*_0_ (×10^**–4**^ cm^–1^)	*g*_⊥_ or *g*_*x*_, *g*_*y*_	*g*_∥_ or *g*_*z*_	*A*_⊥_ or *A*_*x*_, *A*_*y*_ (×10^–4^ cm^–1^)	*A*_∥_ or *A*_*z*_ (×10^–4^ cm^–1^)	calculated^d^*g*_0,calc_	suggested coordination
L = DO4S								
CuL^2+^(1)	2.091	71.7						[4N]
CuL^2+^(2)	2.103	63.6	2.048, 2.058	2.209	20.3, 23.5	171.2	2.105	[4N]S_ax_
L = DO3S								
Cu^2+^	2.196	34.9	2.085	2.423	11.8	127.2	2.197	
CuL^2+^(1)	2.093	74.0	2.036	2.184	15.6	179.3	2.085	[4N]
CuL^2+^(2)			2.048, 2.058	2.209	20.3, 23.5	171.2	2.105	[4N]S_ax_
L = DO2A2S								
Cu^2+^			2.085	2.423	11.8	127.2	2.197	
CuLH_2_^2+^(1)			2.066	2.257	11.5	158.1	2.129	[3N,S]
CuLH_2_^2+^(2)			2.058	2.214	28.7	164,7	2.110	[4N]S_ax_
CuLH^+^			2.060	2.234	25.8	161,5	2.118	[3N,O]N_ax_
CuL			2.075	2.272	24.5	142.8	2.141	[2N,2O]2N_ax_
L = Cyclen^e^								
CuL			2.040, 2.055	2.197	16.9, 21.0	181.9	2.097	[4N]H_2_O_ax_
L = DOTA^f^								
CuL^2–^(1)			2.058	2.301	10.0	150.0	2.139	[2N,2O]2N_ax_
CuL^2–^(2)			2.061	2.241	15.0	157.2	2.121	[3N,O]N_ax_

a The literature data for Cu^2+^-DOTA and Cu^2+^-cyclen are reported for comparison.

b The experimental error was ±0.001 for *g*_0_ and ±1 × 10^–4^ cm^–1^ for *A*_0_.

c The experimental error was ±0.002 for *g*_*x*_ and *g*_*y*_, ±0.001 for *g*_*z*_, and ±1 × 10^–4^ cm^–1^ for *A*_*x*_, *A*_*y*_, and *A*_*z*_.

d Calculated by the equation *g*_0,calc_ = (*g*_*x*_ + *g*_*y*_ + *g*_*z*_)/3 on the basis of anisotropic values.

e From ref (41).

f From ref (52).

The room temperature EPR spectra measured for Cu^2+^-DO4S are unaffected by the pH (Figure 4). This indicates that the metal coordination environment does not change in the investigated pH range (1.61–11.60), as expected (Figure 3A). Unfortunately, nitrogen splitting was not well resolved, and, consequently, the number of the coordinated nitrogen donor atoms could not be accurately determined; we assumed this number to be four because also for Cu^2+^-DOTA and Cu^2+^-cyclen all four nitrogen atoms are coordinated to the metal center.^41,52^ The measured spectra can be simulated assuming the presence of two isomeric species in a ca. 50:50 ratio, named CuL^2+^(1) and CuL^2+^(2) (Figure S18). The former was treated with a lower *g*_0_ value, which indicates a stronger ligand field in the equatorial plane, while for the latter, a higher *g*_0_ was considered (Table 3). Because for CuL^2+^(2) *g*_*z*_ > (*g*_*x*_ + *g*_*y*_)/2, this Cu^2+^-DO4S isomer should have elongated axial bonds consistent with distorted square-pyramidal or octahedral geometries, as was also indicated by UV–vis.^41,53^ Therefore, we can hypothesize that CuL^2+^(1) and CuL^2+^(2) have [4N] and [4N,S] coordination, respectively, and in the latter, sulfur should bind copper axially (the notation [4N]S_ax_ was used in Table 3). As a comparison, for the Cu^2+^-cyclen complex, the geometry is square-pyramidal, with four nitrogen atoms in the equatorial plane and one oxygen atom (from H_2_O or anions) in the apical plane, and in this symmetrical arrangement, *g*_*z*_ was found to be significantly lower and *A*_*z*_ higher (Table 3).^41^

The spectra recorded at 77 K for Cu^2+^-DO4S were described with the superposition of an usual spectrum component originating from a Cu^2+^ complex with a distorted geometry and an isotropic singlet spectrum (Figure 4). The latter can be originated from an aggregation of paramagnetic species in which a dipole–dipole interaction causes the line broadening. For the usual spectrum, the average *g*_0_ value (2.105) is very close to the measured *g*_0_ of CuL^2+^(2) (2.103) detected at room temperature, so that this isomer likely becomes predominant at 77 K. Different from room temperature, at 77 K the ratio of the isotropic spectra varies depending on the pH (Figure S18); however, this change can be due to differences in the freezing conditions.

The room temperature EPR spectra of Cu^2+^-DO3S were simulated with the spectrum of one CuL^2+^ species and the spectrum of free copper at the acidic pH range (Figure 4). Because the examined solution was freshly prepared before the measurements, the low complexation rate described above justifies the presence of the free metal ion at low pH. The obtained *g*_0_ and *A*_0_ values of the CuL^2+^ complex formed by DO3S are very close to those of the CuL^2+^(1) isomer formed by DO4S, pointing out the same coordination mode (Table 3). At low temperature, besides the free copper, two isomeric components can be detected for Cu^2+^-DO3S with a 55:45 ratio (Figures 4 and S19). Both spectra show an usual elongated octahedral or square-pyramidal geometry, and the calculated *g*_0_ values suggest the same coordination environment as the two isomers CuL^2+^(1) and CuL^2+^(2) observed for DO4S at room temperature.

DFT calculations have been performed on Cu^2+^-DO4S and Cu^2+^-DO3S complexes to gain theoretical support for their structure in solution. A preliminary conformational analysis indicated that the complexes having four coordinated nitrogen atoms are the most stable. These isomers were investigated by evaluating the relative stability of the Cu^2+^ complexes in which zero, one, or two sulfide arms, i.e., [4N], [4N,S], and [4N,2S], respectively, are coordinated to the metal center (Figure S20). The results are shown in Table 4.

**Table 4:** Electronic and Gibbs Free Energies (in the Gas Phase and in Water) for the DO4S and DO3S Complexes of Cu^2+^ and Cu^+^^a^

			gas phase		water	
M	ligand	coordination	Δ*E*	Δ*G*	Δ*E*	Δ*G*
Cu^2+^	DO4S	[4N]	–412.4	–399.4	–192.7	–179.7
		[4N,S]	–417.4	–404.2	–190.9	–177.7
		[4N,2S]	–410.1	–396.6	–179.8	–166.2
	DO3S	[4N]	–411.4	–399.5	–196.6	–184.8
		[4N,S]	–418.1	–403.8	–197.4	–183.1
		[4N,2S]	–411.2	–395.5	–185.9	–170.3
Cu^+^	DO4S	[4N]	–117.3	–104.7	–60.6	–48.0
		[4N,S]	–128.3	–115.4	–68.9	–56.0
		[4N,2S]	–122.5	–108.5	–60.6	–46.6
	DO3S	[4N]	–119.7	–108.6	–63.4	–52.4
		[4N,S]	–130.6	–117.1	–71.4	–57.9
		[4N,2S]	–126.2	–111.4	–63.9	–49.0

a All energies are in kilocalories per mole. Level of theory: (COSMO-)ZORA-OPBE/TZ2P//ZORA-OPBE/TZP.

For both ligands, the Δ*G*_water_ values for the [4N] and [4N,S] complexes are particularly close: because the accuracy of the computed energies is on the order of ±1 kcal/mol, it is reasonable to assume that both isomers are present in an aqueous environment. These two isomers likely correspond to the CuL^2+^(1) and CuL^2+^(2) species detected also by EPR experiments. As well, the sulfur bonding indicated by the UV–vis spectra of Cu^2+^-DO4S and Cu^2+^-DO3S shown in Figure 2 can now be attributed to the presence in solution of the [4N,S] species, which as seen accounts for around half of the Cu^2+^ complexes. The coordination of a second sulfur atom is disfavored for both ligands because the final [4N,2S] complex has a less negative Δ*G*_water_ of more than 10 kcal/mol compared to those of the [4N] and [4N,S] complexes.

The activation strain model (ASM) and energy decomposition analysis (EDA) have been used in the gas phase to rationalize the origin of the theoretical preference of these Cu^2+^ complexes to bind either zero or one sulfide (Table S6). The strain energy (Δ*E*_strain_) of Cu^2+^-DO4S increases by a value of 7.5 kcal/mol when passing from [4N] to [4N,S], which is the energy required to bring one extended pendant to the form it has in the coordinated metal complex. However, the [4N,S] complex shows a more stabilizing interaction energy (Δ*E*_int_) of 12.5 kcal/mol over the [4N] one mainly because of a less destabilizing Pauli repulsion (Δ*E*_Pauli_), so that these two complexes result in similar total energy contents. The [4N,2S] complex is destabilized compared to the [4N,S] one because it requires an additional strain energy of 6.7 kcal/mol to bend and coordinate a new pendant to the metal, whereas the interaction energy is virtually unaffected. For Cu^2+^-DO3S, the energy differences were very similar and can be interpreted analogously to that for Cu^2+^-DO4S.

Attempts were made to obtain suitable crystals for Cu^2+^-DO4S and Cu^2+^-DO3S in order to perform structural investigations also in the solid state through single-crystal X-ray diffraction. Such attempts were successful for Cu^2+^-DO4S.

**Figure 5:**
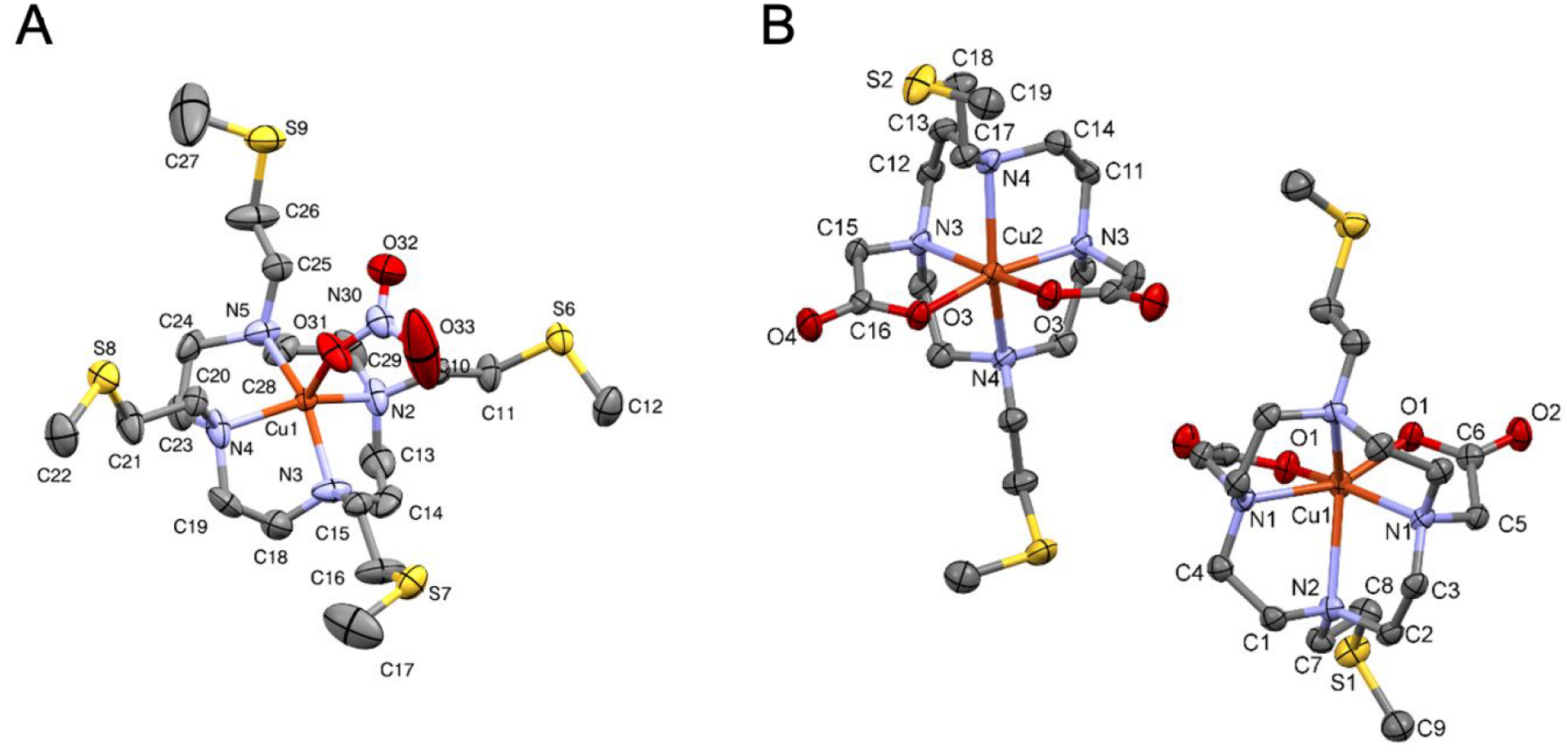
ORTEP diagrams of (A) [Cu(DO4S)(NO_3_)]·NO_3_ and (B) [Cu(DO2A2S)] (Cu1 = molecule #1; Cu2 = molecule #2) with atom numbering. Thermal ellipsoids are drawn at the 50% probability level. Water molecules, hydrogen atoms, and nonbonded nitrate anions are omitted for the sake of clarity. The symmetry codes for molecules #1 and #2 in [Cu(DO2A2S)] are −*x* + 1, *y*, −*z* + 1 and −*x* + 2, *y*, −*z* + 1, respectively.

A view of the crystal structure of [Cu(DO4S)(NO_3_)]·NO_3_ is shown in Figure 5, and selected bond distances and angles are gathered in Table 5. Crystal data and refinement details are provided in Table S7. The complex crystallizes in the monocline space group, and the asymmetrical unit contains a CuL^2+^ molecule and two nitrate anions. Each Cu^2+^ ion is surrounded by four nitrogen atoms of the macrocyclic ring and a nitrate anion in a square-pyramidal geometry. The average bond distances between the metal center and the nitrogen atoms (2.04 Å) are close to those observed for N4–Cu complexes like [Cu(cyclen)(NO_3_)](NO_3_).^54^ Sulfur atoms do not form any bond with Cu^2+^ in the crystal because they are more than 5.0 Å away from the metal center and together form an S4 plane, coplanar to the N4 plane. The structure of [Cu(DO4S)(NO_3_)]·NO_3_ likely resembles that of the [4N] isomer CuL^2+^(1) detected in solution by EPR and computed by DFT (see above).

**Table 5:** Selected Bond Lengths and Angles of the Cu^2+^ Coordination Environments in the Crystal Structures of [Cu(DO4S)(NO_3_)]·NO_3_ and of Both Molecules of [Cu(DO2A2S)]^a^

		[Cu(DO2A2S)]			
[Cu(DO4S)(NO_3_)]·NO_3_		molecule #1		molecule #2	
bond	distance (Å)	bond	distance (Å)	bond	distance (Å)
Cu1–N5	2.03(7)	Cu1–O1	1.954(2)	Cu2–O3	1.955(2)
Cu1–N3	2.04(7)	Cu1–N1	2.150(3)	Cu2–N3	2.110(3)
Cu1–N2	2.05(7)	Cu1–N2	2.536(3)	Cu2–N4	2.336(3)
Cu1–N4	2.06(7)				
Cu1–O31	2.15(6)				

		[Cu(DO2A2S)]			
[Cu(DO4S)(NO_3_)]·NO_3_		molecule #1		molecule #2	
bond	angle (deg)	bond	angle (deg)	bond	angle (deg)
N5–Cu1–N2	86.8(3)	O1–Cu1–N1	80.3(1)	O3–Cu2–N3	84.1(1)
N5–Cu1–N3	151.9(3)	N1–Cu1–N1^#1^	117.2(2)	N3–Cu2–N3^#2^	103.3(2)
N5–Cu1–N4	87.6(3)	N2–Cu1–N2^#1^	125.6(2)	N4–Cu2–N4^#2^	149.9(1)
N5–Cu1–O31	104.6(3)	O1–Cu1–O1^#1^	87.0(1)	O3–Cu2–O3^#2^	89.6(1)
N3–Cu1–O31	103.3(3)	O1–Cu1–N1^#1^	157.49(9)	O3–Cu2–N3^#2^	169.4(1)
N2–Cu1–O31	110.5(3)				
N4–Cu1–O31	98.7(3)				

a See Figure 5 for atom labeling. Additional data are summarized in Tables S8, S9, S11, and S12. Symmetry codes: #1, −*x* + 1, *y*, −*z* + 1; #2, −*x* + 2, *y*, −*z* + 1.

Turning to Cu^2+^-DO2A2S, Figures 2 and S8 show that the UV–vis spectra of Cu^2+^-DO2A2S solutions at equilibrium are markedly different from those of Cu^2+^-DO4S, Cu^2+^-DO3S, and Cu^2+^-DO3SAm. At pH >2, where the complex CuL exists, a high-energy charge-transfer absorption band centered at around 272 nm, and a weaker d–d transition at 715 nm were found. The close similarity to the absorption band maxima of the CuL^2–^ complex formed by DOTA (Figure S21B) suggests an analogous distorted octahedral coordination environment where the Cu^2+^ ion is bound with a [2N,2O] equatorial arrangement and with the two other nitrogen donors in the axial position.^52,55,56^ The less prominence of the shoulder at 310 nm (Figure S21B), compared to Cu^2+^-DOTA, may indicate that the Jahn–Teller distortion is partially quenched in the Cu^2+^-DO2A2S complex.

Under highly acidic pH (<2), the absorbance in the UV region of Cu^2+^-DO2A2S is slightly dropped with a simultaneous broadening and red shift from 276 to 303 nm (Figure 2), while in the visible region, the band is blue-shifted from 715 to 680 nm (Figure S16). These findings can be attributed to the formation of a different complex, namely, CuLH^+^ (Figure 3). Also DOTA forms protonated complexes at acidic pH,^52^ but the band shifts observed for DO2A2S were not detected: the Cu^2+^-DOTA bands only change in intensity because of the lower electron density of the amine groups upon the protonation of noncoordinated carboxylates, while the d–d band is almost pH-insensitive because the protonation of distant nonbonding carboxylates does not exert a marked influence in the electronic structure of the metal complex (Figure S21A).^56^ It can be deduced that for Cu^2+^-DO2A2S the protonation of the carboxylic groups imposes more severe structural changes to the coordination sphere than for Cu^2+^-DOTA. Interestingly, the UV–vis absorption spectrum of Cu^2+^-DO2A2S at highly acidic pH becomes similar to those of the Cu^2+^ complexes formed by the pure sulfur-bearing ligands (DO4S, DO3S, and DO3SAm), so that an analogous coordination geometry may be inferred; i.e., one sulfur atom can be supposed to be involved in the metal coordination. Unlike amines and carboxylates, sulfur donors do not undergo acid–base competitive protonation equilibria and can coordinate metal ions also at strongly acidic pH.

**Figure 6:**
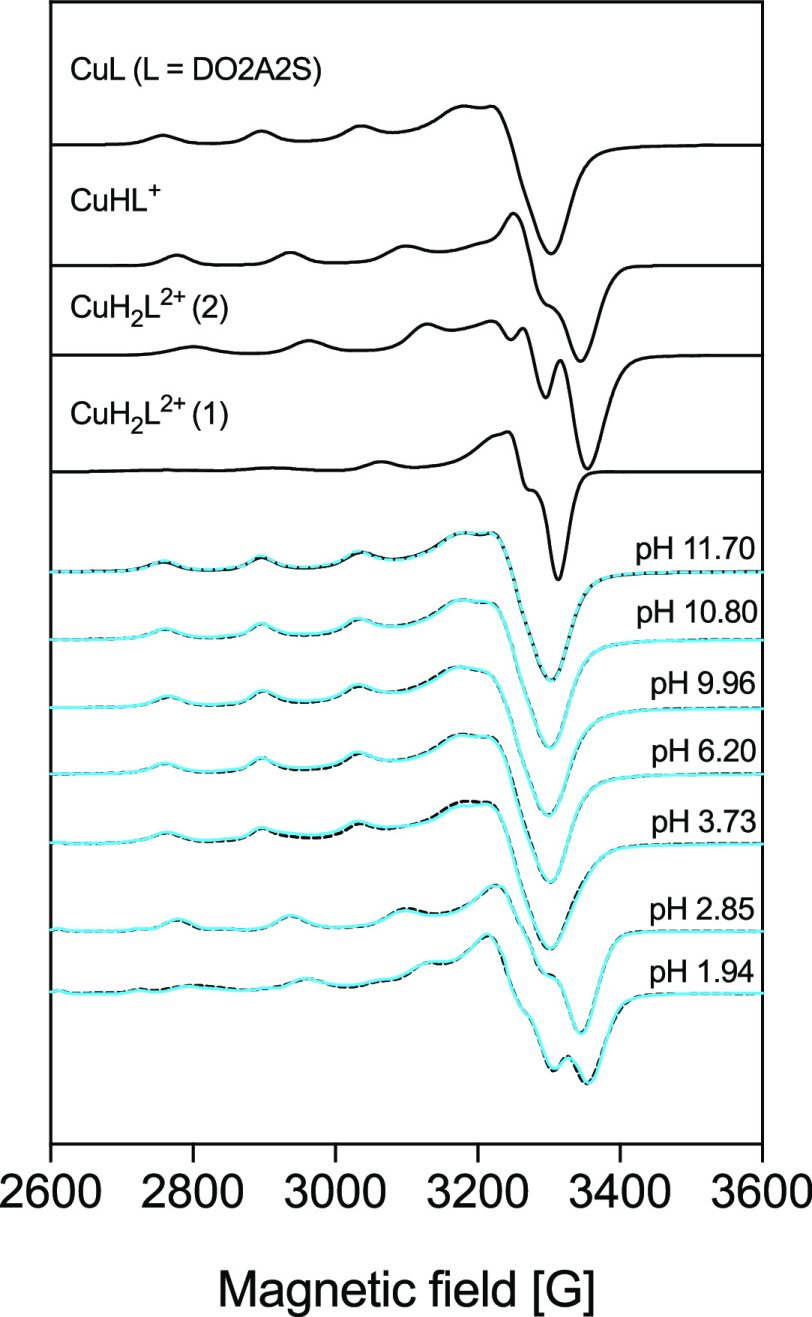
Measured (solid lines) and simulated (dotted lines) spectra for solutions containing Cu^2+^ and DO2A2S (*C*_Cu_^2+^ = 1.0 × 10^–3^ mol/L; *C*_DO2AS_ = 1.1 × 10^–3^ mol/L) at 77 K. The component spectra obtained from the simulation are shown in the upper part.

Solutions containing Cu^2+^ and DO2A2S were examined also by EPR, but the signal intensity was very low at room temperature, so that it was possible to simulate only the spectra of frozen solutions (Figure 6 and Table 3). In comparison, anisotropic EPR parameters of Cu^2+^-DOTA complexes measured at different pH values^52^ were also collected in Table 3. For Cu^2+^-DOTA at pH ∼7, two differently coordinated isomers were detected, indicated as CuL^2–^(1) and CuL^2–^(2) (Table 3). The spectra for Cu^2+^-DO2A2S show a clear pH dependence (Figure 6) because an increase in the proton content causes a noticeable change in the profiles, similar to what was observed in the UV–vis investigation. Above pH 3.73, one spectrum becomes predominant and its EPR parameters are near those of the CuL^2–^(1) isomer formed by DOTA, suggesting a similar [4N,2O] coordination environment with two axially bound nitrogen atoms ([2N,2O]2N_ax_; Table 3), as was also deduced from the electronic spectra. At pH 2.85, a CuLH^+^ complex was detected, and its parameters are close to those of the CuL^2–^(2) isomer formed by DOTA. At pH 1.94, two-component spectra could be detected, which were assigned as CuLH_2_^2+^(1) and CuLH_2_^2+^(2) (Figures 6 and S22). The EPR parameters of the latter are similar to those of the CuL^2+^(2) isomer formed by DO4S. Deprotonation of the carboxylate groups causes a substantial rearrangement of the structure, which results in a higher *g*_*z*_ value compared to those of the protonated complexes (Table 3). In the UV–vis spectra, this appeared as a red shift of the λ_max_ value (Figure S16) because the *g*_*i*_ and *A*_*i*_ values are related to the electronic transitions by the factors derived from the ligand-field theory.^41,57^ Different from the UV–vis data, EPR reports also the presence of a bisprotonated species, and it accounts for this species, rather than for the monoprotonated one, the involvement of sulfur in the coordination sphere. The very large temperature difference (room temperature and 77 K) among the two data sets can explain this disagreement.

The coordination of Cu^2+^-DO2A2S as a function of the pH was further investigated by DFT (Table 6). When both carboxylates are deprotonated, the most stable structure is achieved through a double coordination by the oxygen donors on the Cu^2+^ metal center: the formed bonds are particularly strong (Δ*G*_water_ = −206.7 kcal/mol) thanks to the anionic nature of the two pendants. When one of the carboxylates is protonated, the corresponding bond is weakened, as Δ*G*_water_ is reduced by almost 20 kcal/mol. Detachment of the protonated acetate group is possible and leads to a more stable structure, with the remaining anionic carboxylate group coordinated to the metal. In these conditions, no coordination of the sulfur arm is likely to occur, from an energetic point of view, because it does not contribute to stabilization of the final complex. Such DFT predictions agree very well with the EPR experimental results. When, finally, both carboxylate arms are protonated (situation not shown in Table 6), they do not bind the metal center. A situation analogous to that of DO4S and DO3S originates, so that one additional isomer can form involving one sulfur atom in the metal binding, as suggested from the UV–vis and EPR spectra.

**Table 6:** Electronic and Gibbs Free Energies (in the Gas Phase and in Water) for the DO2A2S Complexes of Cu^2+^ and Cu^+^^a^

			gas phase		water	
M	coordination	form^b^	Δ*E*	Δ*G*	Δ*E*	Δ*G*
Cu^2+^	[4N,2O]		–698.8	–684.7	–220.8	–206.7
	[4N,2O]	H^+^	–563.3	–548.3	–202.6	–187.6
	[4N,O]	H^+^	–565.7	–550.1	–210.5	–194.9
	[4N,O,S]	H^+^	–563.8	–546.4	–201.0	–183.7
	[4N,S]	H^+^	–554.6	–539.4	–198.8	–183.6
	[4N,2S]	H^+^	–545.5	–528.2	–186.7	–169.4
Cu^+^	[4N,2O]		–260.6	–260.6	–66.3	–57.2
	[4N,O]		–257.7	–257.7	–75.5	–66.0
	[4N,O,S]		–253.1	–253.1	–71.4	–61.0
	[4N,S]		–248.1	–248.1	–77.8	–67.8
	[4N,2S]		–243.7	–243.7	–69.1	–56.2
	[4N,2O]	H^+^	–193.4	–193.4	–63.1	–50.5
	[4N,O]	H^+^	–203.1	–203.1	–73.1	–59.8
	[4N,O,S]	H^+^	–199.6	–199.6	–68.7	–54.1
	[4N,S]	H^+^	–188.1	–188.1	–96.9	–82.4
	[4N,2S]	H^+^	–184.2	–184.2	–65.2	–48.7

a All of the energies are in kilocalories per mole. Level of theory: (COSMO-)ZORA-OPBE/TZ2P//ZORA-OPBE/TZP.

b The two carboxylates were considered to be either deprotonated (−) or monoprotonated (H^+^).

A crystal of Cu^2+^-DO2A2S suitable for a crystallographic analysis, [Cu(DO2A2S)], was obtained from water at neutral pH. The complex crystallizes in the monocline crystal system in the *I*2 space group, and the unit cell contains four neutral CuL molecules without the inclusion of counterions or solvent molecules. The crystal structure of [Cu(DO2A2S)] is shown in Figure 5, and the unit cell and packing arrangements viewed from the different crystallographic directions are shown in Figures S23 and S24. Selected bond distances and angles are gathered in Table 5. Crystal data and refinement details are provided in Table S10. The asymmetrical unit contains two complexes (molecule #1 and #2) with slightly different coordination geometries. In both molecules, Cu^2+^ is positioned in a 2-fold rotation axis that mirrors half of the complexes. Two carboxylates and four nitrogen atoms, but no sulfides, are clearly involved in the metal binding, in agreement with the Cu^2+^-DO2A2S structural data obtained in solution from UV–vis, EPR, and DFT in similar pH conditions where the crystal was formed. The coordination geometry for both molecules is a distorted octahedron with [2N,2O]2N_ax_ coordination similar to the crystal structure of Cu^2+^-DOTA.^55^ The axial N–Cu–N angle deviates significantly from the ideal 180° because it is 129.6(2)° for molecule #1 and 149.9(1)° for molecule #2 (Table 5). The conformations of the two [Cu(DO2A2S)] molecules and that of Cu^2+^-DOTA are compared in Figure S25.

### Electrochemical Properties

The Cu^2+^ complexes formed by DO4S, DO3S, and DO2A2S were examined in aqueous solutions at nearly physiological pH (∼7) by CV.

In the cyclic voltammogram of the unbound Cu^2+^ (Figure S26), a cathodic peak for the reduction of Cu^2+^ to Cu^+^ was observed at about −0.08 V versus saturated calomel electrode (SCE), while two overlapping peaks were found on the backward scan due to the oxidation of Cu^+^ and the anodic stripping of Cu^0^ deposited on the electrode because of Cu^+^ dismutation during the scan.

The cyclic voltammograms of the investigated free ligands are shown in Figure S27. DO4S, DO3S, and DO2A2S were demonstrated to be electrochemically inactive in the potential range of the Cu^2+^/Cu^+^ redox couple, i.e., from +0.5 to −0.5 V versus SCE. At about 0.8 V versus SCE, DO4S and DO3S showed small oxidation peaks, whereas DO2A2S exhibited a well-developed anodic peak. The oxidation processes underlying these peaks were not further examined because of their low intensity (DO4S and DO3S) and proximity to the anodic electrolyte discharge. The anodic peak of DO2A2S might be assigned to the oxidation of its carboxylic groups. DO4S and DO3S bear oxidizable thioethers, but the observed anodic peaks cannot be assigned to oxidation of the sulfanyl side chains because the typical oxidation potentials of these groups are higher than 1.0 V.^58−60^ It is more likely that they are due to impurities in the ligands resulting from their synthesis.

**Figure 7:**
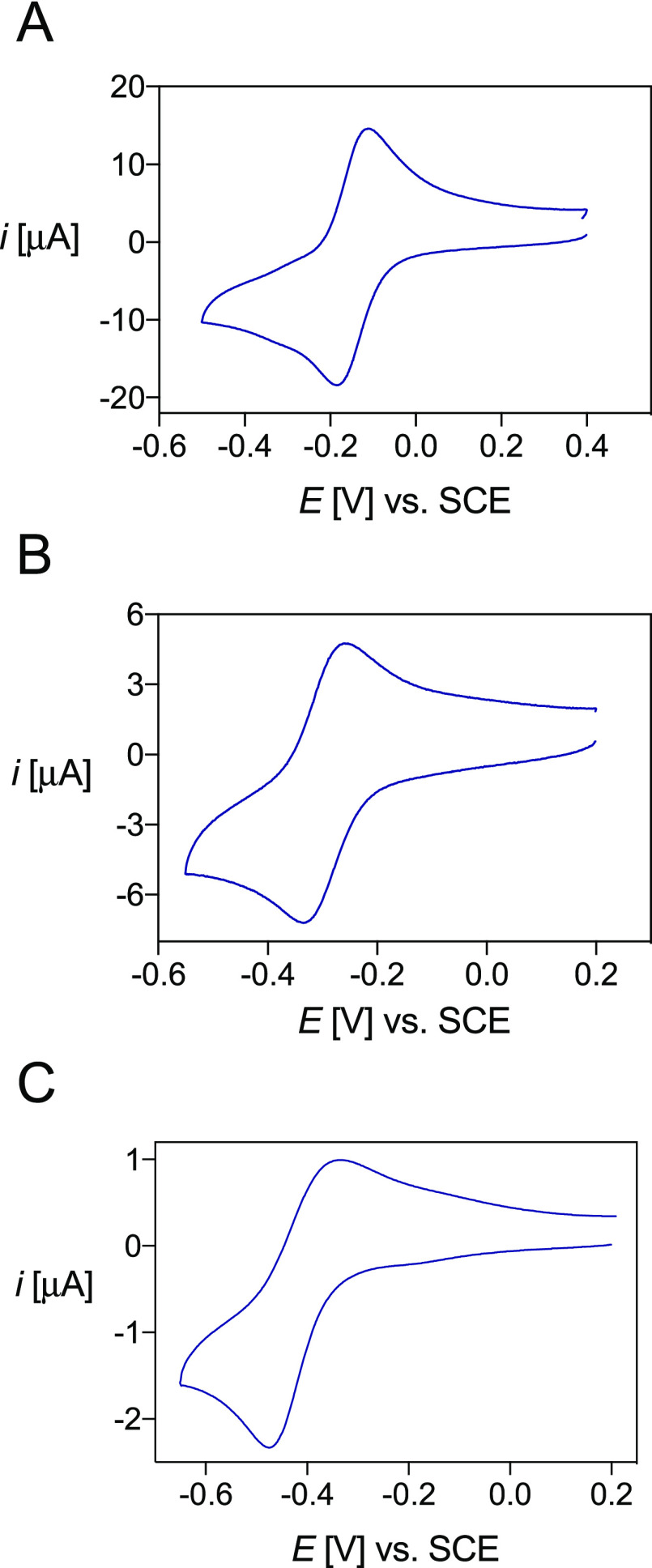
Cyclic voltammograms of the copper complexes of (A) DO4S (*C*_[Cu(DO4S)]^2+^_ = 1.02 × 10^–3^ mol/L), (B) DO3S (*C*_[Cu(DO3S)]^2+^_ = 1.13 × 10^–3^ mol/L), and (C) DO2A2S (*C*_[Cu(DO2A2S)]_ = 6.48 × 10^–4^ mol/L) in aqueous solution at pH 7, *I* = NaNO_3_ 0.15 mol/L, and *T* = 25 °C. Scan rates: 0.1 V/s (A and B) and 0.01 V/s (C).

Typical cyclic voltammograms of the copper-ligand complexes are presented in Figure 7, while their electrochemical properties are summarized in Table 7. At physiological pH, all solutions exhibited two peaks assigned to the redox couple of the Cu^2+^/Cu^+^ complexes (Figure 7). This voltammteric behavior did not change with time or after multiple reduction/oxidation cycles, indicating that no demetalation with copper loss occurs after Cu^2+^ reduction. The long-time stability of Cu^+^ complexes was confirmed by controlled-potential electrolysis, which allowed *in situ* preparation of the chelates, followed by NMR characterization (see below).

**Table 7:** Cathodic Peak Potential (*E*_pc_), Anodic Peak Potential (*E*_pa_), and Half-wave Potential (*E*_1/2_) for Copper Complexes of DO4S, DO3S, and DO2A2S in Aqueous Solution at pH 7, *I* = 0.15 mol/L NaNO_3_, and *T* = 25 °C

complex	*E*_pc_ [V] vs SCE^a^	*E*_pa_ [V] vs SCE^a^	Δ*E*_p_ [V] vs SCE^a^	*E*_1/2_ [V] vs SCE^a^
Cu-DO4S	–0.182 ± 0.001	–0.115 ± 0.003	0.067	–0.149 ± 0.001
Cu-DO3S	–0.334 ± 0.004	–0.252 ± 0.003	0.082	–0.293 ± 0.005
Cu-DO2A2S	–0.496^b^	–0.341^b^	0.155^b^	–0.421 ± 0.004

a Average of the values measured at 0.01 V/s ≤ *v* ≤ 0.2 V/s.

b Value at *v* = 0.01 V/s.

Variation of the scan rate did not modify the voltammetric pattern of Cu-DO4S and Cu-DO3S; only the current intensity changed with the scan rate (Figure S28). Electron transfer (ET) to Cu^2+^ complexes with these ligands was quite fast, with Δ*E*_p_ = *E*_pa_ – *E*_pc_ values slightly higher than the canonical 60 mV for Nernstian ET processes. Conversely, Δ*E*_p_ for Cu-DO2A2S was much higher than 60 mV and remarkably increased as the scan rate was raised, indicating the occurrence of quasi-reversible ET (Figure S28). The value of Δ*E*_p_ = 155 mV measured at *v* = 0.01 V/s increased to 260 mV at *v* = 0.1 V/s. At higher scan rates, the process tended toward the behavior of irreversible ET with a drastic decrease of the anodic peak in the reverse scan. For all complexes, the cathodic peak current (*i*_pc_) varied linearly with *v*^1/2^, indicating that all electrode processes are under diffusion control (Figure S29), and the voltammetric analyses allowed us to conclude that no demetalation occurs when Cu^2+^ is reduced to Cu^+^, with all ligands being able to accommodate both copper oxidation states.

Differences were evidenced in the redox kinetics: ET was essentially reversible for the Cu^2+^ complexes of DO4S and DO3S, while sluggish kinetics were observed for Cu-DO2A2S. The activation Gibbs free energy of ET for Cu-DO4S and Cu-DO3S should mainly arise from solvent reorganization, while a significant contribution from inner reorganization is also present in the case of Cu-DO2A2S. A plausible conformational change accompanying ET to Cu^2+^-DO2A2S might be the decoordination of one or two acetate arms and the simultaneous coordination of one or two sulfur atoms to form a stable Cu^+^-DO2A2S complex.

The obtained electrochemical data can also give insights into the ability of the Cu^2+^ complexes to withstand reductive-induced decomplexation *in vivo*. The standard reduction potentials of the Cu^2+^ complexes were calculated from CV, assuming that *E*^0^ = *E*_1/2_ = (*E*_pa_ + *E*_pc_)/2 (Table 7). The estimated threshold for typical bioreductants (*E*^0^ = −0.64 V vs SCE) is more negative than the *E*_1/2_ values of Table 7. Therefore, all of the investigated copper complexes are likely to be reduced in the presence of biological reductants.^34^ However, the stability observed by CV strongly suggests that the resulting Cu^+^ complexes would not undergo demetalation.

CV was previously used to evaluate the ability of Cu^2+^ chelates to withstand reductive-induced demetalation. Several Cu^2+^ complexes with macrocyclic compounds such as TETA and CB-DO2A exhibited irreversible cyclic voltammograms, suggesting instability of electrogenerated Cu^+^ chelates.^4,16^ Conversely, all complexes investigated here undergo one-electron reduction to give highly stable Cu^+^ chelates, as shown by CV and confirmed by controlled-potential electrolysis (see below).

### Solution Thermodynamics and Structural Investigation of the Cuprous Complexes

The stability constants of the Cu^+^ complexes were calculated using the electrochemical data and the stability constants of the corresponding Cu^2+^ complexes, as described in the Supporting Information. It was also assumed that the complex formed between Cu^+^ and each ligand at pH 7 is CuL^+^ because Cu^2+^ (see above), Cd^2+^, and Ag^+^^37,38^ also form this complex under the same conditions. The results are summarized in Table 8, together with the calculated pCu^+^ values (pCu^+^ = −log [Cu^+^]_free_) at different pH values, which indicate that DO4S forms the most stable Cu^+^ complexes.

**Table 8:** Overall Stability Constants (logβ) for the Cu^+^ Complexes Formed by DO4S, DO3S, and DO2A2S at *I* = 0.15 mol/L and *T* = 25°C and Calculated pCu^+^ Values at Different pH Values^a^

			pCu^+^		
ligand	equilibrium reaction	logβ	pH 4.0	pH 6.0	pH 7.4
DO4S	Cu^+^ + L ⇋ CuL^+^	19.8 ± 0.2	10.7	14.7	17.2
DO3S	Cu^+^ + L ⇋ CuL^+^	17.2 ± 0.2	7.9	11.9	14.5
DO2A2S	Cu^+^ + L^2–^ ⇋ CuL^–^	16.7 ± 0.1	7.3	11.3	14.1

a pCu^+^ calculated at *C*_Cu_^+^ = 10^–6^ mol/L and *C*_L_ = 10^–5^ mol/L

**Figure 8:**
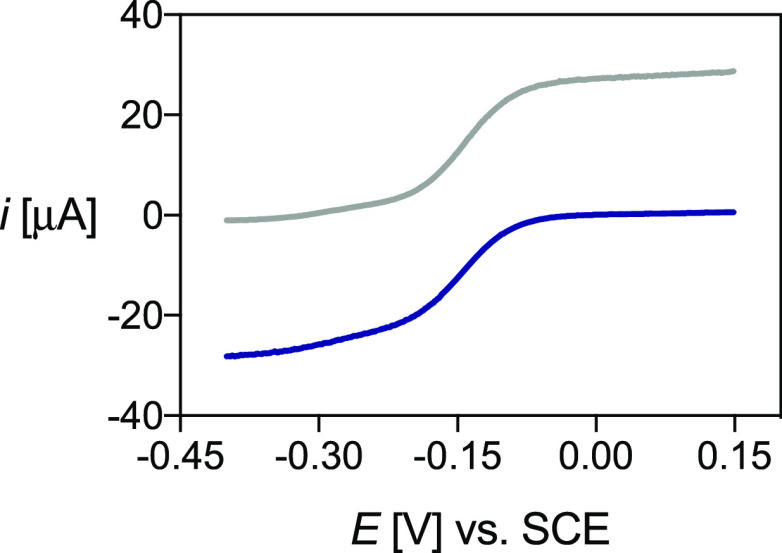
LSV of Cu-DO4S before (blu) and after (gray) electrolysis at −0.35 V, performed with a rotating disk electrode at ω = 2000 rpm and *v* = 0.005 V/s, with *I* = NaNO_3_ 0.15 mol/L and *T* = 25 °C.

Bulk electrolyses of Cu^2+^-DO4S and Cu^2+^-DO2A2S solutions were performed at nearly neutral pH to isolate and characterize the corresponding Cu^+^ complexes. Linear-scan voltammetry (LSV) was used to monitor the evolution of the species in solution. A representative example of LSV before and after electrolysis is reported in Figure 8. The Cu^+^ complexes of both ligands remain stable at least for some hours after their formation.

**Figure 9:**
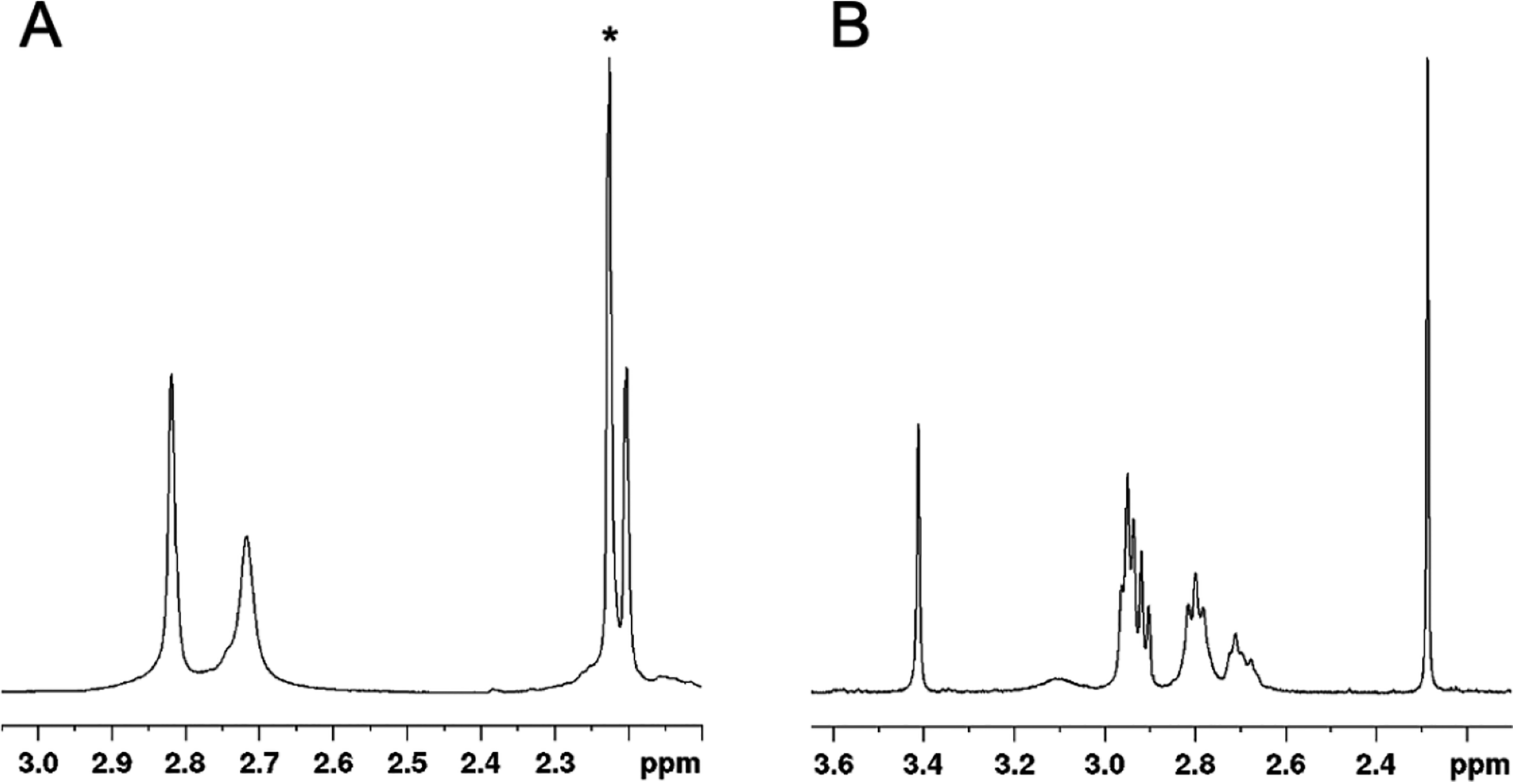
^1^H NMR spectra (400 MHz, room temperature, H_2_O + 10% D_2_O) of the in situ generated cuprous complexes: (A) DO4S (*C*_Cu_*= C*_DO4S_ = 1.6 × 10^–3^ mol/L) and (B) DO2A2S (*C*_Cu_ = *C*_DO2A2S_ = 1.4 × 10^–3^ mol/L) at pH 7. The signal marked with an asterisk (2.22 ppm) is related to the acetone impurity.

NMR spectra performed on the Cu^+^-ligand solution obtained after electrolysis are shown in Figure 9. The NMR spectral data are summarized in Table S13, and a comparison between the NMR spectra of the free ligands and the respective Cu^+^ complexes, showing significant changes of the proton chemical shifts associated with the complexation event, is reported in Figures S30 and S31.

The ^1^H NMR spectrum of Cu^+^-DO4S (Figure 9) is consistent with the formation of a highly symmetric complex because it exhibits only three signals. The singlet at 2.20 ppm was attributed to the SCH_3_ protons, whereas those at 2.72 and 2.82 ppm include all other protons. According to the peak integrations, these are the NCH_2_ protons of the pendant arms, and the ring NCH_2_ together with SCH_2_, but from the monodimensional spectrum, it is not possible to state which signal belongs to which protons. The downfield shift observed for the SCH_3_ protons upon Cu^+^ complexation (from ca. 2.18 ppm for the monoprotonated free ligand^37^ to 2.20 ppm for the Cu^+^ complex; Figure S30) suggests the formation of Cu^+^–S bond(s). Indeed, all sulfur-related signals are equivalent on the NMR time scale, suggesting either that all four sulfur atoms are bound or that their exchange is rapid on the NMR time scale. Considering also the reversible voltammetric pattern, which suggests a similar coordination for the Cu^+^ and Cu^2+^ complexes, it is possible to argue that all of the ring nitrogen atoms and one rapidly exchanging sulfur are present in the metal coordination sphere of Cu^+^-DO4S. In the case of Ag^+^-DO4S solutions, the metal ion was likely bound by two nitrogen and two sulfur atoms.^38^ If the ^1^H NMR spectrum of Cu^+^-DO4S is compared with that of Ag^+^-DO4S (Figure S32),^38^ the metal coordination seems to be different because the signals change in shape and position.

DFT calculations performed on Cu^+^-DO4S and Cu^+^-DO3S complexes confirm that one sulfur atom is bound to Cu^+^ (Table 4). The Cu^+^ complexes of DO4S and DO3S are stabilized in the [4N,S] coordination mode by 6–8 kcal/mol compared to the [4N] one. The coordination of a second sulfur atom to Cu^+^, giving a [4N,2S] coordination, is disfavored because a less negative Δ*G*_water_ is obtained (by ∼9 kcal/mol if compared to [4N,S]). Using ASM and EDA (Table S6), it can be observed that stabilization of the [4N,S] complex is assigned mainly to the contribution of the interaction energy (Δ*E*_int_) and the orbital interaction term (Δ*E*_oi_). The destabilization experienced by the addition of a second sulfide is due to an increased strain contribution (Δ*E*_strain_).

A Kohn–Sham molecular orbital (KS-MO) analysis has been performed for Cu^+^-DO4S to explain the reason behind the more stabilizing Δ*E*_oi_ of the [4N,S] complex compared to the [4N] one. The electron density donation from the highest occupied molecular orbital (HOMO)–3 orbital of the ligand (Figure S33) to the 4s orbital of Cu^+^ [lowest unoccupied molecular orbital (LUMO)] was found to be the strongest interaction and the principal bonding force of the [4N] complex. The same interaction is also present in the [4N,S] and [4N,2S] complexes, with the only difference being that the donating orbitals are HOMO–4 and HOMO–5, respectively. This orbital interaction is slightly more efficient in the [4N,S] complex because of a lower energy gap and a higher overlap between the metal and ligand orbitals. However, the main Δ*E*_oi_ stabilization originates from a secondary bonding mode, which is active only when a sulfide pendant group directly coordinates the metal center, namely, the electron donation that occurs from the HOMO of the ligand to the LUMO+1 (4p_*z*_ orbital) of the metal center (Figure S34).

Controlled-potential electrolysis of Cu^2+^-DO2A2S confirmed the formation of a stable Cu^+^-DO2A2S species. ^1^H NMR spectra for this complex indicate a decreased ligand flexibility upon Cu^+^ coordination because both ring and side-arm protons gave signals narrower than those of the free ligand (Figure S31). The transannular sulfur-donor atoms appear to be involved in the Cu^+^ binding because the SCH_3_ (2.28 ppm) signals of the complex are significantly downfield-shifted compared to the monoprotonated free ligand (2.15 ppm^37^), and also the SCH_2_ signal pattern of the chelator changes considerably upon Cu^+^ complexation. This result, combined with the CV data, can represent proof that coordination sphere switching occurred when Cu^2+^ was reduced to Cu^+^. The Cu^+^-DO2A2S NMR spectra are similar to those obtained for Ag^+^-DO2A2S (Figure S35), but signals are narrower when Cu^+^ is coordinated, which might indicate that the cuprous complex is characterized by a slowed-down fluxional interconversion compared to the Ag^+^ one.^38^

The stability of the Cu^+^-DO2A2S complexes was investigated by DFT, particularly tackling any possible change in coordination due to carboxylate protonation. When no protonation occurs, two structures are predominant and reflect the most probable Cu^+^ complex geometries (Table 6): they are both coordinated (in the apical region, i.e., above the metal center) by a single chain in which the [4N,S] species is ∼2 kcal/mol more stable than the [4N,O] one. The protonation of a single carboxylate group results into two intriguing effects. First, the relative stability among the different types of coordination does not change with respect to the unprotonated structures. Second, the [4N,S] complex is now greatly stabilized by 22.6 kcal/mol compared to the [4N,O] one, thus further favoring the formation of a Cu^+^ complex with a single sulfur chain coordinated to the metal center.

## Experimental Section

### Materials

All chemicals were purchased from commercial suppliers (Sigma-Aldrich, Fluka, and VWR Chemicals) and used as received. 1,4,7,10-Tetrakis[2-(methylsulfanyl)ethyl]-1,4,7,10-tetraazacyclododecane (DO4S), 1,4,7-tris[2-(methylsulfanyl)ethyl]-1,4,7,10-tetraazacyclododecane (DO3S), 1,4,7-tris[2-(methylsulfanyl)ethyl]-10-acetamido-1,4,7,10-tetraazacyclododecane (DO3SAm), and 1,7-bis[2-(methylsulfanyl)ethyl]-4,10-diacetic acid-1,4,7,10-tetraazacyclododecane (DO2A2S) were synthesized according to previously published procedures.^37^ 1,4,7,10-Tetraazacyclododecane-1,4,7,10-tetraacetic acid (DOTA) was obtained from Chematech. All solutions were prepared in ultrapure water (Purelab Chorus, Veolia).

### Complexation Kinetics

The kinetics of the reactions between Cu^2+^ and DO4S, DO3S, DO3SAm, DO2A2S, and DOTA were investigated using UV–vis spectroscopy on a Cary 60 UV–vis spectrophotometer (Agilent) in the range from 200 to 800 nm using a quartz spectrophotometric cell of 1 cm path length at room temperature. Equimolar amounts of Cu^2+^ and the corresponding ligand were mixed in buffered aqueous solutions at pH 2.0 (1.0 × 10^–2^ mol/L HCl), 3.0 (1.0 × 10^–3^ mol/L HCl), 4.8 (acetic/acetate), and 7.0 (2-[4-(2-hydroxyethyl)piperazin-1-yl]ethanesulfonic acid buffer). Concentrations ranged from 1.0 × 10^–4^ to 1.0 × 10^–3^ mol/L. The UV–vis spectra were collected immediately after mixing at different time points. The complexation reaction was monitored directly by an increase of the charge-transfer or d–d bands at the characteristic wavelengths.

### Thermodynamic Measurements

Hydrochloric acid (HCl; Sigma-Aldrich, 37%, 1 and 0.1 mol/L) and carbonate-free 0.1 mol/L sodium hydroxide (NaOH; Fluka, 99% min) solutions were prepared. The former was standardized against sodium carbonate (Na_2_CO_3_; Aldrich, 99.95–100.5%) and the latter against 0.1 mol/L HCl. Ligand stock solutions were prepared at ∼2.0 × 10^–3^ mol/L, while Cu^2+^ stock solutions were prepared at ∼2.0 × 10^–3^ mol/L from an analytical-grade chloride salt (CuCl_2_·2H_2_O; Sigma-Aldrich, 99.9%) by the dissolution of weighted compounds in a calibrated volumetric flask. All stock solutions were stored at 4 °C. The ionic strength (*I*) was fixed to 0.15 mol/L with sodium chloride (NaCl; Fluka, 99%), unless otherwise stated. Each experiment was performed independently at least three times.

The potentiometric measurements were carried out as reported previously,^37,38^ but the starting pH was brought to ∼4, taking into account the complexation kinetic measurements.

UV–vis pH-spectrophotometric titrations were carried out by the out-of-cell and in-cell methods in the pH range 0–3 and from pH ≥3, respectively, at room temperature. In the first method, stock solutions of the ligands and CuCl_2_ were mixed in independent vials to obtain a 1:1 metal-to-ligand molar ratio (final concentrations ∼10^–4^ mol/L), and different amounts of 1 mol/L HCl were added to adjust the pH. The vials were sealed, heated to 80 °C in a thermostated bath to ensure complete complexation of Cu^2+^, and then cooled to room temperature and opened. The absorption spectra were recorded on a Cary 60 UV–vis spectrophotometer (Agilent) in the range from 200 to 800 nm using a quartz spectrophotometric cell of 1 cm path length. The equilibrium was considered to be reached when no variations of the UV–vis spectra were detected. A similar procedure was adopted to determine the lowest ligand protonation constant of the ligands, but in this case, no metal ion was added and no heating was needed. Direct titrations were carried out in a 3 mL water-jacketed glass cell maintained at 25.0 ± 0.1 °C using a Haake F3 cryostat. Removal of the atmospheric CO_2_ prior to and during the titration was ensured by a constant flow of purified nitrogen. The ligand concentration in the titration cell was varied in the range 5 × 10^–5^–2 × 10^–4^ mol/L, and the metal-to-ligand ratios were between 1:1 and 1:2. The solutions were acidified with a known volume of HCl, and the titrations were carried out by accurate NaOH additions (approximately microliters). The pH was measured with a Mettler Toledo pH-meter equipped with a glass electrode daily calibrated with commercial buffer solutions (pH 4.0, 7.0, and 9.0), except in very acidic solutions (pH <2), where it was computed from the HCl concentration (pH = −log *C*_HCl_). After each addition, the pH was allowed to equilibrate, a sample aliquot was transferred to the spectrophotometric cell, and the spectrum was recorded. The aliquot was transferred back to the titration vessel, and new additions were made up to a pH of around 11.

UV–vis spectrophotometric titrations were performed by adding known volumes of a Cu^2+^ solution to the chelator one (∼1 × 10^–4^ mol/L), buffered at pH 4.8 by acetic/acetate. Metal-to-ligand ratios ranged between 0 and 3. The UV–vis spectra were recorded, and the stoichiometry was determined by plotting the absorbance at the characteristic wavelength as a function of the metal-to-ligand ratios [*n*(Cu^2+^)/*n*(L)].

Titrations with Ag^+^ as a competitor were performed using UV–vis spectroscopy at pH 4.8 (acetic/acetate buffer) without control of the ionic strength. Batch titration points were prepared by adding varying amounts of Cu^2+^ to a solution containing the preformed Ag^+^ complex (*C*_Ag_ = *C*_L_ ∼ 1 × 10^–4^ mol/L). Different metal-to-metal ratios, between 0 and 4, were attained. Because of the slow kinetics of the transmetalation reactions at room temperature, solutions were brought to equilibrium by heating at ∼55 °C before the UV–vis spectra measurements. Equilibrium was considered to be reached when the UV–vis spectra did not change.

The overall equilibrium constants (logβ_*pqr*_ = [M_*p*_L_*q*_H_*r*_]/[M]^*p*^[L]^*q*^[H]^*r*^) were obtained by refinement of the thermodynamic data using the *PITMAP* software^61^ and refer to the overall equilibria *p*M^*m*+^ + *q*H^+^ + *r*L^*l*–^ ⇆ M_*p*_H_*q*_L_*r*_^*pm*+*q*–*rl*^, where M is the metal ion and L the nonprotonated ligand molecule. The errors quoted are the standard deviations calculated by the fitting program. The constants for ligand protonation, Cu^2+^ hydroxo species, and, in the case of the competition titrations, also the Ag^+^ complexes were taken from the literature.^37,38,62^

### EPR Measurements

All EPR spectra were recorded using a Bruker EleXsys E500 spectrometer (microwave frequency 9.54 GHz, microwave power 13 mW, modulation amplitude 5 G, and modulation frequency 100 kHz). The pH-dependent EPR spectra were recorded in a freshly prepared solution containing (1.1–1.3) × 10^–3^ mol/L ligand (DO4S, DO3S, and DO2A2S) and 1.0 × 10^–3^ mol/L CuCl_2_, in the pH range 1.8–12. NaOH and HCl solutions were employed to adjust the pH. The ionic strength was fixed using 0.15 mol/L NaCl. The room temperature EPR spectra were collected in capillaries recording 12 scans. For the frozen solution spectra, 0.2 mL samples were diluted with 0.05 mL of methanol to avoid the crystallization of water and transferred into EPR tubes. Anisotropic EPR spectra were recorded in a Dewar containing liquid nitrogen at 77 K. The room temperature spectra were corrected by subtracting the background spectrum of pure water. The spectra were simulated by the “EPR” program^63^ using the parameters *g*_0_ and *A*_0_, copper hyperfine (*I*_Cu_ = ^3^/_2_) coupling, and three linewidth parameters. The anisotropic EPR spectra were analyzed with the same program. Rhombic or axial **g** tensor (*g*_*x*_, *g*_*y*_, and *g*_*z*_) and copper hyperfine tensor (*A*_*x*_^Cu^, *A*_*y*_^Cu^, and *A*_*z*_^Cu^) have been used. Orientation-dependent parameters (α, β, and γ) were used to fit the linewidths through the equation σ_MI_ = α + β*M*_*i*_ + γ*M*_*i*_^2^, where *M*_*i*_ denotes the magnetic quantum number of the copper nucleus. Because natural Cu^2+^ was used for the measurements, the spectra were calculated as the sum of the spectra of ^63^Cu and ^65^Cu weighted by their natural abundances (69.17% and 30.83%, respectively). The hyperfine and superhyperfine coupling constants and relaxation parameters were obtained in field units (gauss = 10^–4^ T).

### X-ray Crystal Structure

Blue crystals of [Cu(DO4S)(NO_3_)]·NO_3_ and [Cu(DO2A2S)] suitable for X-ray diffraction were obtained in solutions containing equimolar amounts of metal and ligand. For DO4S, slow evaporation of a methanol solution was performed, whereas for DO2A2S, crystals arose in water at pH ∼7 set with NaOH. X-ray measurements were made at room temperature on a Nicolet P3 (for Cu^2+^-DO4S) and a Rigaku RAXIS-RAPID II (for Cu^2+^-DO2A2S) diffractometer using numerical absorption correction with graphite-monochromated Mo Kα radiation.^64^ The structures were solved with direct methods, and missing atoms were determined by difference Fourier techniques and refined according to the least-squares method against *F*^2^. For Cu^2+^-DO4S, disordered side chains of molecules have been refined isotropically into two conformations, and all non-hydrogen atoms were refined anisotropically. In general, carbon-bound hydrogen atoms were geometrically located and refined as riding. The isotropic displacement parameters of the hydrogen atoms were approximated from the *U*(eq) value of the atom to which they were bonded. For Cu^2+^-DO4S, the *SHELX 93* crystallographic software package was used,^65^ and the details about data collection and structure refinement are given in Table S7. For Cu^2+^-DO2A2S, the *CrystalClear* software was used.^66^ The *SIR2014*(67) and *SHELX*(68) program packages under *WinGX*(69) software were used to solve the structure and for its refinement. The data collection and refinement parameters are listed in Table S10. The selected bond lengths and angles of Cu^2+^-DO2A2S were calculated by *PLATON* software.^70^ The graphical representation and the edition of the CIF files were done by *Mercury*(71) and *EnCifer*(72) software. The structures were deposited with CCDC 2036253 for [Cu(DO4S)(NO_3_)]·NO_3_ and CCDC 2078038 for [Cu(DO2A2S)].

### CV

CV was carried out in a six-necked cell equipped with three electrodes and connected to an Autolab PGSTAT 302N potentiostat, interfaced with *NOVA 2.1* software (Metrohm) at room temperature. The CV experiments were performed using a glassy-carbon working electrode (WE) fabricated from a 3-mm-diameter rod (Tokai GC-20). The counter electrode (CE) was a platinum wire, and the reference electrode was a saturated calomel electrode (SCE). Before each experiment, the working electrode surface was cleaned by polishing with 0.25 μm diamond paste, followed by ultrasonic rinsing in ethanol for 5 min. All electrochemical experiments were performed in a ∼1 × 10^–3^ mol/L aqueous solution of preformed Cu^2+^ complexes. The pH of the solutions was adjusted to 7 with NaOH and/or HNO_3_ solutions. NaNO_3_ was used as the supporting electrolyte at a 0.15 mol/L concentration without purification. The sample solutions were degassed by bubbling N_2_ before all measurements and kept under a N_2_ stream during the measurements. Cyclic voltammograms with scan rates ranging from 0.005 to 0.2 V/s were recorded in the region from −0.5 to 0.5 V. At this potential range, the solvent with the supporting electrolyte and the free ligands were found to be electroinactive.

### Electrolysis and NMR

Exhaustive electrolyses of the preformed Cu^2+^ complexes of DO4S and DO2A2S (∼1 × 10^–3^ mol/L) were carried out with a glassy-carbon WE. The CE was a platinum foil separated from the working solution through a glass double frit (G3) filled with a conductive solution (0.15 mol/L NaNO_3_), and the reference electrode was SCE. The electrolyses were performed at *E* = −0.35 and −0.75 V for Cu-DO4S and Cu-DO2A2S, respectively. LSV was used to monitor the evolution of the species in solution. Each electrolysis was considered to be complete when the cathodic current reached <2% of the initial value.

The *in situ* generated Cu^+^ complexes of DO4S and DO2A2S were transferred into NMR tubes using a Schlenk line to avoid the presence of O_2_. ^1^H NMR spectra were recorded at room temperature on a 400 MHz Bruker Avance III HD spectrometer. The water signal was suppressed using an excitation sculpting pulse scheme.^73^ Proton chemical shifts are reported in parts per million.

### DFT Calculations

All DFT calculations were performed with the Amsterdam Density Functional (*ADF*) program.^74−76^ The OPBE^77−79^ generalized gradient approximation density functional was used, in combination with two basis sets: geometry optimizations and frequency analysis have been carried out with the TZP (triple-ζ quality augmented with one set of polarization functions on each atom), whereas the final energy evaluation has been done with the TZ2P (triple-ζ quality and is augmented with two sets of polarization functions on each atom). Scalar relativistic effects were accounted for using the zeroth-order regular approximation (ZORA).^80^ This level of theory is denoted in the text as ZORA-OPBE/TZ2P//ZORA-OPBE/TZP. All of the calculations were performed in the gas phase and in water; for the latter case, the solvation effects have been quantified using the COSMO (COnductor-like Screening MOdel) approach (level of theory: COSMO-ZORA-OPBE/TZ2P//ZORA-OPBE/TZP).^81−84^ A radius of 1.93 Å and a relative dielectric constant of 78.39 were used. The empirical parameter in the COSMO equation was considered to be 0.0. The radii of the atoms are the classical MM3 radii divided by 1.2. Equilibrium geometries were optimized under no symmetry constraint using analytical gradient techniques. All structures were verified by frequency calculations: for all energy minima, only real frequencies associated with the vibrational normal modes were found.

TheActivation Strain Model (ASM), also known as the distortion/interaction model, has been used to understand the nature of the metal–ligand chemical bonding. It is a fragment-based approach to understanding chemical reactions and the associated barriers.^85^ The starting point is two separate reactants, which approach from infinity and begin to interact and deform each other. In this model, the energy Δ*E* is decomposed into the strain energy Δ*E*_strain_ and interaction energy Δ*E*_int_ (eq 1):
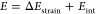
1Δ*E*_strain_ is the energy associated with deformation of the reactants from their relaxed geometries into the structure they acquire in the product. Δ*E*_int_ is the actual interaction energy between the deformed fragments/reactants. The latter can be further analyzed in the framework of the Kohn−Sham Molecular Orbitals (KS-MO) model using a quantitative decomposition of the bond into a purely electrostatic interaction (Δ*V*_elstat_), Pauli repulsion (Δ*E*_Pauli_, called also exchange or overlap repulsion), and (attractive) orbital interactions (Δ*E*_oi_) (eq 2).
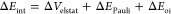
2

## Conclusions

A series of cyclen derivatives bearing sulfide pendant arms, namely, DO4S, DO3S, DO3SAm, and DO2A2S, were considered as Cu^2+^ complexing agents in view of their possible use as BFCs in ^64^Cu- and ^67^Cu-based radiopharmaceuticals.

The thermodynamic data indicate that these ligands possess high affinity toward Cu^2+^, which is a prerequisite for any BFC to securely deliver the radiometals to tumor cells. The complex stability is comparable or even higher than that of well-known Cu^2+^ chelators like DOTA, NOTA, and TETA.

The most probable solution structures of Cu^2+^-DO4S and Cu^2+^-DO3S involve the copresence of isomers having either no or one coordinated sulfide atom. A crystal was obtained for Cu^2+^-DO4S in which the ligand coordinates the metal ion through its four nitrogen atoms. For Cu^2+^-DO2A2S, the same coordination as that for Cu^2+^-DOTA was detected at pH values above ∼4. This structure was found also in the solid state on a crystal obtained for Cu^2+^-DO2A2S. The Cu^2+^-DO2A2S structure changed at acidic pH, when the carboxylated arms are protonated, because one sulfur atom replaced all carboxylates in the metal-ion binding.

The aim of this work was not only to develop stable Cu^2+^ chelators and to study their structures but especially to propose a class of ligands able to withstand the copper demetalation observed *in vivo* for many cupric BFCs due to the bioreduction of Cu^2+^ to Cu^+^. Although DO4S, DO3S, and DO2A2S are probably not able to prevent the bioreduction of Cu^2+^, their Cu^+^ complexes are highly stable because of the coordination of one sulfur atom to the metal center. This stability might prevent copper demetalation *in vivo*. Their ability to stabilize cupric as well as cuprous ions makes these chelators a promising scaffold for ^64^Cu/^67^Cu complexation.

To fully assess the potential of sulfanyl cyclen derivatives for nuclear medicine applications, further evaluations are necessary. The Cu^2+^-ligand complexes should be investigated to evaluate the kinetics of complex formation at radiolabeling conditions, which imply reduced metal and ligand concentrations. The complex stability or inertness should, in turn, be studied at physiological conditions, such as, *e.g.*, in serum and/or in the presence of competing ligands and metal ions, and at the extremely low concentrations typically attained in the bloodstream. This work can be performed using radioactive copper, and it is now in progress.

## Supporting Information Available

Structures of chelators proposed in the literature for copper-based radiopharmaceuticals, acidity constants and distribution diagrams of the free chelators (DO4S, DO3S, DO3SAm, DO2A2S, and DOTA), equilibration times required to reach equilibrium in Cu^2+^/chelator complex formation, UV–vis spectroscopic data and spectra of Cu^2+^/chelator complexes, EPR-derived isomeric dependence on the pH, extended crystallographic data and unit cell/packing arrangement pictures, DFT-computed free energy and structures of the Cu^2+^/chelator and Cu^+^/chelator complexes, cyclic voltammograms of free Cu, free chelators, and Cu/chelator complexes at various scan rates, and NMR data and spectra of Cu^+^/chelator complexes (PDF)

## Accession Codes

CCDC 2036253 and 2078038 contain the supplementary crystallographic data for this paper. These data can be obtained free of charge via www.ccdc.cam.ac.uk/data_request/cif, or by emailing data_request@ccdc.cam.ac.uk, or by contacting The Cambridge Crystallographic Data Centre, 12 Union Road, Cambridge CB2 1EZ, UK; fax: +44 1223 336033.
