# Supplementary material for: Copper Coordination Chemistry of Sulfur Pendant Cyclen Derivatives: An Attempt to Hinder the Reductive-Induced Demetalation in 64/67Cu Radiopharmaceuticals
Source: Inorg Chem. 2021 Jul 19;60(15):11530–47. doi: 10.1021/acs.inorgchem.1c01550 (PMC8389837; doi:10.1021/acs.inorgchem.1c01550)
Supplement: Supplementary file 1 — ic1c01550_si_001.pdf [file ic1c01550_si_001.pdf]

# Supporting Information

# Copper Coordination Chemistry of Sulfur Pendant Cyclen Derivatives: An Attempt to Hinder the Reductive-Induced Demetallation in $^{64/67}$Cu Radiopharmaceuticals

Marianna Tosato[1], Marco Dalla Tiezza[1], Nóra V. May[2], Abdirisak Ahmed Isse[1], Sonia Nardella[1,3], Laura Orian[1], Marco Verona[3], Christian Vaccarin[3], André Alker[4], Helmut Mäcke[5], Paolo Pastore[1], Valerio Di Marco[1,*]

[1] *Department of Chemical Sciences, University of Padova, via Marzolo 1, 35131 Padova, Italy*

[2] *Centre for Structural Science, Research Centre for Natural Sciences, 1117 Magyar Tudósok Körútja 2, Budapest, Hungary*

[3] *Department of Pharmaceutical Sciences, University of Padova, via Marzolo 8, 35131 Padova, Italy*

[4] *Roche Pharmaceutical Research and Early Development, Roche Innovation Center Basel F. Hoffmann-La Roche Ltd, Grenzacherstrasse 124, 4058 Basel, Switzerland*

[5] *Department of Nuclear Medicine, University Hospital Freiburg, Hugstetterstrasse 55, D-79106 Freiburg, Germany*

*Corresponding author: valerio.dimarco@unipd.it

**Table S1.** Acidity constants (p$K_a$ values) of DO4S, DO3S, DO3SAm, and DO2A2S. The reported uncertainty was obtained by the fitting procedure and represents one standard deviation unit. DOTA and cyclen were also included for comparison purpose.

| **Ligand** | **Equilibrium** (a) | **p$K_a$** |
|---|---|---|
| DO4S | $H_3L^{3+} \rightleftharpoons H_2L^{2+} + H^+$ | 1.9 ± 0.3 (b) |
| | $H_2L^{2+} \rightleftharpoons HL^+ + H^+$ | 7.29 (c) |
| | $HL^+ \rightleftharpoons L + H^+$ | 10.14 (c) |
| DO3S | $H_3L^{3+} \rightleftharpoons H_2L^{2+} + H^+$ | 2.0 ± 0.1 (b) |
| | $H_2L^{2+} \rightleftharpoons HL^+ + H^+$ | 7.54 (c) |
| | $HL^+ \rightleftharpoons L + H^+$ | 10.86 (c) |
| DO3SAm | $H_3L^{3+} \rightleftharpoons H_2L^{2+} + H^+$ | 1.9 ± 0.2 (b) |
| | $H_2L^{2+} \rightleftharpoons HL^+ + H^+$ | 7.8 (c) |
| | $HL^+ \rightleftharpoons L + H^+$ | 10.42 (c) |
| DO2A2S | $H_4L^{2+} \rightleftharpoons H_3L^+ + H^+$ | 1.8 ± 0.1 (b) |
| | $H_3L^+ \rightleftharpoons H_2L + H^+$ | 3.44 (c) |
| | $H_2L \rightleftharpoons HL^- + H^+$ | 18.30 (c) |
| | $HL^- \rightleftharpoons L^{2-} + H^+$ | |
| DOTA | $H_6L^{2+} \rightleftharpoons H_5L^+ + H^+$ | 1.34 (d) |
| | $H_5L^+ \rightleftharpoons H_4L + H^+$ | 2.29 (d) |
| | $H_4L \rightleftharpoons H_3L^- + H^+$ | 4.63 (e) |
| | $H_3L^- \rightleftharpoons H_2L^{2-} + H^+$ | 4.86 (e) |
| | $H_2L^{2-} \rightleftharpoons HL^{3-} + H^+$ | 9.18 (e) |
| | $HL^{3-} \rightleftharpoons L^{4-} + H^+$ | 11.43 (e) |
| Cyclen | $H_4L^{4+} \rightleftharpoons H_3L^{3+} + H^+$ | 0.8 (f) |
| | $H_3L^{3+} \rightleftharpoons H_2L^{2+} + H^+$ | 1.6 (f) |
| | $H_2L^{2+} \rightleftharpoons HL^+ + H^+$ | 9.51 (c) |
| | $HL^+ \rightleftharpoons L + H^+$ | 10.63 (c) |

(a) L denotes the ligand in its totally deprotonated form
(b) This work, no ionic strength control
(c) From ref. [1], $I$ = 0.15 mol/L $NaNO_3$, $T$ = 25°C (see reference list at the end of this file)
(d) From ref. [2], $I$ = 0.15 mol/L $(NMe_4)Cl$, $T$ = 25°C
(e) From ref. [3], $I$ = 0.15 mol/L NaCl, $T$ = 25°C
(f) From ref. [4], $I$ = 0.5 mol/L $KNO_3$, $T$ = 25°C

**Table S2.** Approximate time required to reach equilibrium during the reaction between $Cu^{2+}$ and ligand at various pH. Data were taken from **Figures S4 - S7**.

| pH | Equilibration time | | | | |
|---|---|---|---|---|---|
| | DO4S | DO3S | DO3SAm | DO2A2S | DOTA |
| 2.0 | ~ 10 d | ~ 10 d | ~ 10 d | ~ 4 h | ~ 1 h |
| 3.0 | ~ 20 h | ~ 20 h | ~ 10 h | ~ 10 min | ~ 5 min |
| 4.8 | ~ 50 min | ~ 30 min | ~ 1 h | < 10 sec | < 10 sec |
| 7.0 | < 10 sec | < 10 sec | < 10 sec | < 10 sec | < 10 sec |

**Table S3.** Approximate time required to reach equilibrium during the reaction between $Cu^{2+}$ and ligand at various pH. Higher concentrations than those of **Table S2** were employed for the reactants.

| pH | Equilibration time | | |
|---|---|---|---|
| | DO4S | DO3S | DO2A2S |
| 2.0 [(a)] | – | – | ~ 20 min |
| 3.0 [(b)] | ~ 2 h | ~ 5 h | < 1 min |
| 4.8 [(c)] | ~ 3 min | ~ 2 min | < 10 sec |
| 7.0 [(d)] | < 10 sec | < 10 sec | < 10 sec |

[(a)] pH 2.0: $C_{DO2A2S} = C_{Cu^{2+}} = 1.0 \cdot 10^{-3}$ mol/L
[(b)] pH 3.0: $C_{DO4S,\ DO3S} = C_{Cu^{2+}} = 9.0 \cdot 10^{-4}$ mol/L; $C_{DO2A2S} = C_{Cu^{2+}} = 8.0 \cdot 10^{-4}$ mol/L
[(c)] pH 4.8: $C_{DO4S} = C_{Cu^{2+}} = 1.2 \cdot 10^{-3}$ mol/L; $C_{DO3S,\ DO2A2S} = C_{Cu^{2+}} = 1.1 \cdot 10^{-3}$ mol/L
[(d)] pH 7.0: $C_{DO4S,\ DO3S,\ DO2A2S} = C_{Cu^{2+}} = 1.0 \cdot 10^{-3}$ mol/L

**Table S4.** UV-Vis spectroscopic data of the $Cu^{2+}$-ligand complexes.

| Ligand | Complex | Transition | $\lambda_{max}$ (nm) | $\varepsilon$ calculated [(a)] (L/cm·mol) |
|---|---|---|---|---|
| DO4S | $CuL^{2+}$ | CT | 309 | $(3.6 \pm 0.9)\cdot10^3$ |
| | | *d-d* | 593 | $5.3\cdot10^2$ [(b)] |
| DO3S | $CuL^{2+}$ | CT | 303 | $(3.6 \pm 0.2)\cdot10^3$ |
| | | *d-d* | 581 | $4.3\cdot10^2$ [(b)] |
| DO3SAm | $CuL^{2+}$ | CT | 304 | $(3.7 \pm 0.8)\cdot10^3$ |
| | | *d-d* | 602 | $3.2\cdot10^2$ [(b)] |
| DO2A2S | CuL | CT | 272 | $(5.0 \pm 0.2)\cdot10^3$ |
| | | *d-d* | 715 | $1.6\cdot10^2$ [(b)] |
| | $CuHL^+$ | CT | 303 | $(4.0 \pm 0.9)\cdot10^3$ |
| | | *d-d* | 680 | – |

[(a)]: obtained from the UV-Vis data fitting aimed at the determination of the equilibrium constants.
[(b)]: estimated from the spectra

**Table S5.** Electronic and Gibbs free energies (in gas-phase and in water) for the Cyc4Me and DO4S complexes of $Cu^{2+}$, supposing no sulfur coordination in the latter. All the energies are in kcal/mol. Level of theory: (COSMO-)ZORA-OPBE/TZ2P//ZORA-OPBE/TZP.

| Ligand | M | Gas phase | | Water | |
|---|---|---|---|---|---|
| | | $\Delta E$ | $\Delta G$ | $\Delta E$ | $\Delta G$ |
| Cyc4Me | $Cu^{2+}$ | –402.5 | –387.1 | –198.4 | –183.0 |
| DO4S | | –412.4 | –399.4 | –192.7 | –179.7 |

**Table S6.** Activation Strain Model (ASM) and Energy Decomposition Analysis (EDA) of the DO4S and DO3S complexes of $Cu^{2+}$ and $Cu^{+}$. All the energies are in kcal/mol. Level of theory: ZORA-OPBE/TZ2P//ZORA-OPBE/TZP.

| M | Ligand | coordination | Δ*E* | Δ$E_{strain}$ | Δ$E_{int}$ | Δ$E_{Pauli}$ | Δ$V_{elstat}$ | Δ$E_{oi}$ |
|---|---|---|---|---|---|---|---|---|
| $Cu^{2+}$ | DO4S | [4N] | –412.4 | 13.7 | –426.1 | 166.5 | –234.6 | –358.0 |
| | | [4N,S] | –417.4 | 21.2 | –438.6 | 142.7 | –228.3 | –353.0 |
| | | [4N,2S] | –410.1 | 27.3 | –437.4 | 96.6 | –198.0 | –336.1 |
| | DO3S | [4N] | –411.4 | 12.4 | –423.8 | 168.2 | –241.6 | –350.4 |
| | | [4N,S] | –418.1 | 21.1 | –439.2 | 153.0 | –241.3 | –350.9 |
| | | [4N,2S] | –411.2 | 27.8 | –439.0 | 111.0 | –214.3 | –335.6 |
| $Cu^{+}$ | DO4S | [4N] | –117.3 | 5.6 | –122.9 | 157.3 | –171.1 | –109.0 |
| | | [4N,S] | –128.3 | 13.7 | –142.0 | 161.0 | –179.5 | –123.5 |
| | | [4N,2S] | –122.5 | 21.8 | –144.3 | 141.7 | –164.9 | –121.2 |
| | DO3S | [4N] | –119.7 | 4.9 | –124.5 | 158.9 | –176.3 | –107.2 |
| | | [4N,S] | –130.6 | 13.4 | –144.1 | 160.3 | –182.3 | –122.1 |
| | | [4N,2S] | –126.2 | 18.0 | –144.2 | 140.7 | –166.4 | –118.5 |

**Table S7.** Crystallographic data and refinement details for [Cu(DO4S)($NO_3$)]·($NO_3$).

| | | |
|---|---|---|
| Empirical formula | $C_{20}H_{44}CuN_6O_6S_4$ | |
| Formula weight | 656.39 g/mol | |
| Temperature | 183 (2) | |
| Radiation and wavelength | Mo-Kα, λ = 0.71073 Å | |
| Crystal system | Monoclinic | |
| Space group | P2 (1) | |
| Unit cell dimension | *a* = 8.128 Å | *α* = 90.0° |
| | *b* = 14.879 Å | *β* = 102.86° |
| | *c* = 12.373 Å, | *γ* = 90.0° |
| Volume | 1458.8 $Å^3$ | |
| Z | 2 | |
| Density (calculated) | 1.494 $Mg/cm^3$ | |
| Absorption coefficient, μ | 1.080 $mm^{-1}$ | |
| F(000) | 694 | |
| Crystal colour | blue | |
| Crystal size | 0.6 × 0.4 × 0.4 mm | |
| θ area data collection | 1.69 to 27.52° | |
| Index area | 0 ≤ h ≤ 10, 0 ≤ k ≤ 19, −16 ≤ l ≤15 | |
| Reflections collected | 3760 | |
| Independent reflections | 3462 [R(int) = 0.02951] | |
| Refinement method | Full-matrix least squares on $F^2$ | |
| Data/restraints/parameters | 3462/1/336 | |
| Final R indices [I <2σ(I)] | $R_1$ = 0.0570, w$R_2$ = 0.1387 | |
| R indices (all data) | $R_1$ = 0.0736, w$R_2$ = 0.1527 | |
| Absolute structure parameter | 0.00 ± 3 | |
| Extinction coefficient | 0.00 ± 2 | |
| Differential signals | 0.842 and −0.575 e.$Å^{-3}$ | |

$R_1 = \Sigma \,||F_0|-|F_c||/ \Sigma \,|F_0|$

$wR_2 = \{\Sigma\, [w(|F_0|^2-|F_c|^2)^2/\Sigma\, [w(F_0^4)]\}^{1/2}$

**Table S8.** Bond lengths of $[Cu(DO4S)(NO_3)]\cdot(NO_3)$.

| Bond | Bond length [Å] | Bond | Bond length [Å] |
|---|---|---|---|
| Cu(1)-N(5) | 2.029(7) | S(7)-C(16) | 1.773(10) |
| Cu(1)-N(3) | 2.035(7) | S(8)-C(22) | 1.784(11) |
| Cu(1)-N(2) | 2.053(7) | S(8)-C(21) | 1.805(10) |
| Cu(1)-N(4) | 2.057(7) | S(9)-C(27) | 1.690(2) |
| Cu(1)-O(31) | 2.149(6) | S(9)-C(26) | 1.799(10) |
| N(2)-C(13) | 1.447(12) | C(10)-C(11) | 1.525(11) |
| N(2)-C(10) | 1.469(10) | C(13)-C(14) | 1.420(2) |
| N(2)-C(29) | 1.599(13) | C(15)-C(16) | 1.518(11) |
| N(3)-C(18) | 1.425(12) | C(18)-C(19) | 1.423(14) |
| N(3)-C(15) | 1.477(10) | C(20)-C(21) | 1.524(12) |
| N(3)-C(14) | 1.611(12) | C(23)-C(24) | 1.453(13) |
| N(4)-C(23) | 1.459(12) | C(25)-C(26) | 1.515(13) |
| N(4)-C(20) | 1.482(10) | C(28)-C(29) | 1.447(14) |
| N(4)-C(19) | 1.573(12) | N(39)-O(33) | 1.168(14) |
| N(5)-C(28) | 1.470(12) | N(30)-O(32) | 1.174(14) |
| N(5)-C(25) | 1.482(10) | N(30)-O(31) | 1.229(9) |
| N(5)-C(24) | 1.579(13) | N(34)-O(35) | 1.194(11) |
| S(6)-C(12) | 1.765(11) | N(34)-O(36) | 1.228(12) |
| S(6)-C(11) | 1.811(9) | N(34)-O(37) | 1.245(10) |
| S(7)-C(17) | 1.736(14) | | |

**Table S9.** Bond angles of [Cu(DO4S)($NO_3$)]·($NO_3$).

| Angle | [°] | Angle | [°] |
|---|---|---|---|
| N(5)-Cu(1)-N(3) | 151.9(3) | C(25)-N(5)-C(24) | 110.4(7) |
| N(5)-Cu(1)-N(2) | 86.8(3) | C(28)-N(5)-Cu(1) | 101.9(6) |
| N(3)-Cu(1)-N(2) | 86.7(3) | C(25)-N(5)-Cu(1) | 114.4(5) |
| N(5)-Cu(1)-N(4) | 87.6(3) | C(24)-N(5)-Cu(1) | 103.3(5) |
| N(3)-Cu(1)-N(4) | 84.8(3) | C(12)-S(6)-C(11) | 101.4(5) |
| N(2)-Cu(1)-N(4) | 150.7(3) | C(17)-S(7)-C(16) | 102.1(8) |
| N(5)-Cu(1)-O(31) | 104.6(3) | C(22)-S(8)-C(21) | 100.9(8) |
| N(3)-Cu(1)-O(31) | 103.3(3) | C(27)-S(9)-C(26) | 99.7(8) |
| N8")-Cu(1)-O(31) | 110.5(3) | N(2)-C(10)-C(11) | 114.2(6) |
| N(4)-Cu(1)-O(31) | 98.7(3) | C(10)-C(11)-S(6) | 109.8(6) |
| C(13)-N(2)-C(10) | 115.8(7) | C(14)-C(13)-N(2) | 108.4(8) |
| C(13)-N(2)-C(29) | 110.9(8) | C(13)-C(14)-N(3) | 111.0(8) |
| C(10)-N(2)-C(29) | 108.8(7) | N(3)-C(15)-C(16) | 115.6(6) |
| C(13)-N(2)-Cu(1) | 102.0(6) | C(15)-C(16)-S(7) | 111.9(6) |
| C(10)-N(2)-Cu(1) | 115.4(5) | C(19)-C(18)-N(3) | 108.2(8) |
| C(29)-N(2)-Cu(1) | 103.1(5) | C(18)-C(19)-N(4) | 111.2(7) |
| C(18)-N(3)-C(15) | 116.6(7) | N(4)-C(20)-C(21) | 114.2(7) |
| C(18)-N(3)-C(14) | 109.2(7) | C(20)-C(21)-S(8) | 109.2(6) |
| C(15)-N(3)-C(14) | 108.8(7) | C(24)-C(23)-N(4) | 108.8(7) |
| C(18)-N(3)-Cu(1) | 102.7(6) | C(23)-C(24)-N(5) | 110.9(7) |
| C(15)-N(3)-Cu(1) | 116.0(4) | N(5)-C(25)-C(26) | 114.2(7) |
| C(14)-N(3)-Cu(1) | 102.6(5) | C(25)-C(26)-S(9) | 112.1(7) |
| C(23)-N(4)-C(20) | 114-1(7) | C(29)-C(28)-N(5) | 110.5(9) |
| C(23)-N(4)-C(19) | 111.2(7) | O(33)-N(30)-O(32) | 120.6(12) |
| C(20)-N(4)-C(19) | 110.7(7) | O(33)-N(30)-O(31) | 118.2(11) |
| C(23)-N(4)-Cu(1) | 101.1(5) | O(32)-N(30)-O(31) | 120.0(11) |
| C(20)-N(4)-Cu(1) | 115.3(5) | N(30)-O(31)-Cu(1) | 136.1(5) |
| C(19)-N(4)-Cu(1) | 103.5(5) | O(35)-N(34)-O(36) | 120.0(9) |
| C(28)-N(5)-C(25) | 115.4(8) | O(35)-N(34)-O(37) | 120.2(9) |
| C(28)-N(5)-C(24) | 110.5(7) | O(36)-N(34)-O(37) | 119.7(8) |

**Table S10.** Crystal data and structure refinement for [Cu(DO2A2S)].

| | | |
|---|---|---|
| Empirical formula | $C_{18}H_{34}CuN_4O_4S_2$ | |
| Formula weight | 498.15 | |
| Temperature | 295(2) | |
| Radiation and wavelength | Mo-K$\alpha$, $\lambda$ =0.71073 Å | |
| Crystal system | monoclinic | |
| Space group | *I* 2 | |
| Unit cell dimensions | $a$ = 16.1921(10) Å | $\alpha$ = 90.0° |
| | $b$ = 6.7295(4) Å | $\beta$ = 91.554(10)° |
| | $c$ = 19.970(3) Å, | $\gamma$ = 90.0° |
| Volume | 2175.2(4) $Å^3$ | |
| *Z/Z'* | 4/1 | |
| Density (calculated) | 1.521 $Mg/m^3$ | |
| Absorption coefficient, $\mu$ | 1.228 $mm^{-1}$ | |
| *F*(000) | 1052 | |
| Crystal colour | blue | |
| Crystal description | block | |
| Crystal size | 0.30 × 0.30 × 0.20 mm | |
| Absorption correction | numerical | |
| Max. and min. transmission | 0.928, 0.966 | |
| $\theta$ range for data collection | 3.195 ≤ $\theta$ ≤ 27.468° | |
| Index ranges | $-20 \leq h \leq 20$; $-8 \leq k \leq 8$; $-25 \leq l \leq 25$ | |
| Reflections collected | 23317 | |
| Completeness to 2$\theta$ | 0.998 | |
| Independent reflections | 4964 [$R$(int) = 0.0421] | |
| Reflections $I$>2$\sigma$($I$) | 4599 | |
| Refinement method | full-matrix least-squares on $F^2$ | |
| Data / restraints / parameters | 4964 /1 /266 | |
| Goodness-of-fit on $F^2$ | 1.038 | |
| Final $R$ indices [$I$>2$\sigma$($I$)] | $R_1$ = 0.0298, $wR_2$ = 0.0616 | |
| R indices (all data) | $R_1$ = 0.0345, $wR_2$ = 0.0628 | |
| Max. and mean shift/esd | 0.000; 0.000 | |
| Largest diff. peak and hole | 0.396; –0.202 e.$Å^{-3}$ | |

**Table S11.** Bond lengths for [Cu(DO2A2S)].

| Bond | Bond length [Å] | Bond | Bond length [Å] |
|---|---|---|---|
| Cu1-O1#1 | 1.954(2) | Cu1-O1 | 1.955(2) |
| Cu1-N1#1 | 2.150(3) | Cu1-N1 | 2.150(3) |
| S1-C9 | 1.789(4) | S1-C8 | 1.813(3) |
| O1-C6 | 1.283(4) | O2-C6 | 1.231(4) |
| N1-C5 | 1.481(4) | N1-C3 | 1.482(4) |
| N1-C4#1 | 1.490(4) | N2-C2 | 1.461(4) |
| N2-C1 | 1.476(4) | N2-C7 | 1.478(4) |
| C1-C4 | 1.514(4) | C2-C3 | 1.521(5) |
| C5-C6 | 1.510(5) | C7-C8 | 1.515(5) |
| Cu2-O3#2 | 1.955(2) | Cu2-O3 | 1.955(2) |
| Cu2-N3#2 | 2.110(3) | Cu2-N3 | 2.110(3) |
| Cu2-N4#2 | 2.336(3) | Cu2-N4 | 2.336(3) |
| S2-C19 | 1.785(4) | S2-C18 | 1.812(4) |
| O3-C16 | 1.277(4) | O4-C16 | 1.229(4) |
| N3-C15 | 1.485(4) | N3-C12 | 1.489(4) |
| N3-C11 | 1.490(4) | N4-C14 | 1.463(4) |
| N4-C13 | 1.479(4) | N4-C17 | 1.489(4) |
| C11-C14#2 | 1.523(4) | C12-C13 | 1.528(5) |
| C15-C16 | 1.526(5) | C17-C18 | 1.521(4) |

**Table S12.** Bond angles for [Cu(DO2A2S)].

| Angle | [°] | Angle | [°] |
|---|---|---|---|
| O1#1-Cu1-O1 | 87.0(1) | O1#1-Cu1-N1#1 | 80.3(1) |
| O1-Cu1-N1#1 | 157.49(9) | O1#1-Cu1-N1 | 157.49(9) |
| O1-Cu1-N1 | 80.3(1) | N1#1-Cu1-N1 | 117.2(2) |
| C9-S1-C8 | 101.2(2) | C6-O1-Cu1 | 118.0(2) |
| C5-N1-C3 | 110.3(2) | C5-N1-C4#1 | 107.1(3) |
| C3-N1-C4#1 | 110.8(3) | C5-N1-Cu1 | 102.5(2) |
| C3-N1-Cu1 | 114.5(2) | C4#1-N1-Cu1 | 111.1(2) |
| C2-N2-C1 | 109.9(2) | C2-N2-C7 | 111.4(2) |
| C1-N2-C7 | 110.1(2) | N2-C1-C4 | 111.1(2) |
| N2-C2-C3 | 110.9(2) | N1-C3-C2 | 111.6(3) |
| N1#1-C4-C1 | 112.0(3) | N1-C5-C6 | 110.4(2) |
| O2-C6-O1 | 125.3(3) | O2-C6-C5 | 119.6(3) |
| O1-C6-C5 | 115.1(3) | N2-C7-C8 | 112.5(3) |
| C7-C8-S1 | 112.9(2) | O3#2-Cu2-O3 | 89.6(1) |
| O3#2-Cu2-N3#2 | 84.1(1) | O3-Cu2-N3#2 | 169.4(1) |
| O3#2-Cu2-N3 | 169.4(1) | O3-Cu2-N3 | 84.1(1) |
| N3#2-Cu2-N3 | 103.3(2) | O3#2-Cu2-N4#2 | 107.21(9) |
| O3-Cu2-N4#2 | 94.2(1) | N3#2-Cu2-N4#2 | 79.70(9) |
| N3-Cu2-N4#2 | 81.75(9) | O3#2-Cu2-N4 | 94.2(1) |
| O3-Cu2-N4 | 107.21(9) | N3#2-Cu2-N4 | 81.75(9) |
| N3-Cu2-N4 | 79.70(9) | N4#2-Cu2-N4 | 149.9(1) |
| C19-S2-C18 | 102.2(2) | C16-O3-Cu2 | 117.2(2) |
| C15-N3-C12 | 111.2(2) | C15-N3-C11 | 111.2(3) |
| C12-N3-C11 | 110.4(3) | C15-N3-Cu2 | 104.3(2) |
| C12-N3-Cu2 | 109.7(2) | C11-N3-Cu2 | 109.8(2) |
| C14-N4-C13 | 114.6(3) | C14-N4-C17 | 113.2(3) |
| C13-N4-C17 | 112.5(2) | C14-N4-Cu2 | 101.4(2) |
| C13-N4-Cu2 | 107.6(2) | C17-N4-Cu2 | 106.5(2) |
| N3-C11-C14#2 | 111.3(2) | N3-C12-C13 | 112.0(3) |
| N4-C13-C12 | 113.4(2) | N4-C14-C11#2 | 111.4(2) |
| N3-C15-C16 | 113.7(3) | O4-C16-O3 | 125.8(4) |
| O4-C16-C15 | 118.1(3) | O3-C16-C15 | 116.0(3) |
| N4-C17-C18 | 115.2(3) | C17-C18-S2 | 111.1(2) |

Symmetry codes to generate equivalent atoms: #1 –x+1,y,–z+1 and #2 –x+2,y,–z+1

**Table S13.** $^1$H-NMR resonance assignments for the $Cu^+$ complexes formed by DO4S and DO2A2S (400 MHz, RT, $H_2O$ + 10% $D_2O$).

| Complex | Signal (ppm) | Multiplicity | Area | Proton Assignation |
|---|---|---|---|---|
| $Cu^+$-DO4S | 2.20 | s | 12 | $SCH_3$ |
| | 2.72 | s broad | 16 | $SCH_2$ + $NCH_2$ arms or $NCH_2$ ring |
| | 2.82 | s broad | 16 | $NCH_2$ ring or $SCH_2$ + $NCH_2$ arms |
| $Cu^+$-DO2A2S | 2.28 | s | 6 | $SCH_3$ |
| | 2.71 | m | 4 | $SCH_2$ |
| | 2.80 | t | 8 | $NCH_2$ ring |
| | 2.92 | m | 4 | $NCH_2$ |
| | 2.94 | t | 8 | $NCH_2$ ring |
| | 3.41 | s | 4 | $CH_2COOH$ |

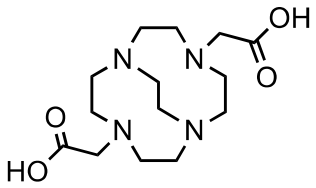

**CB-DO2A**

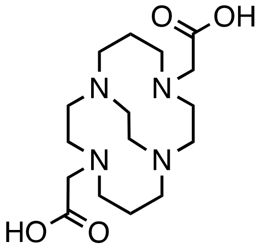

**CB-TE2A**

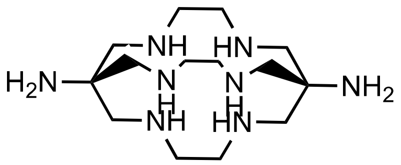

**DiamSar**

**Figure S1.** CB-DO2A, CB-TE2A and DiamSar.

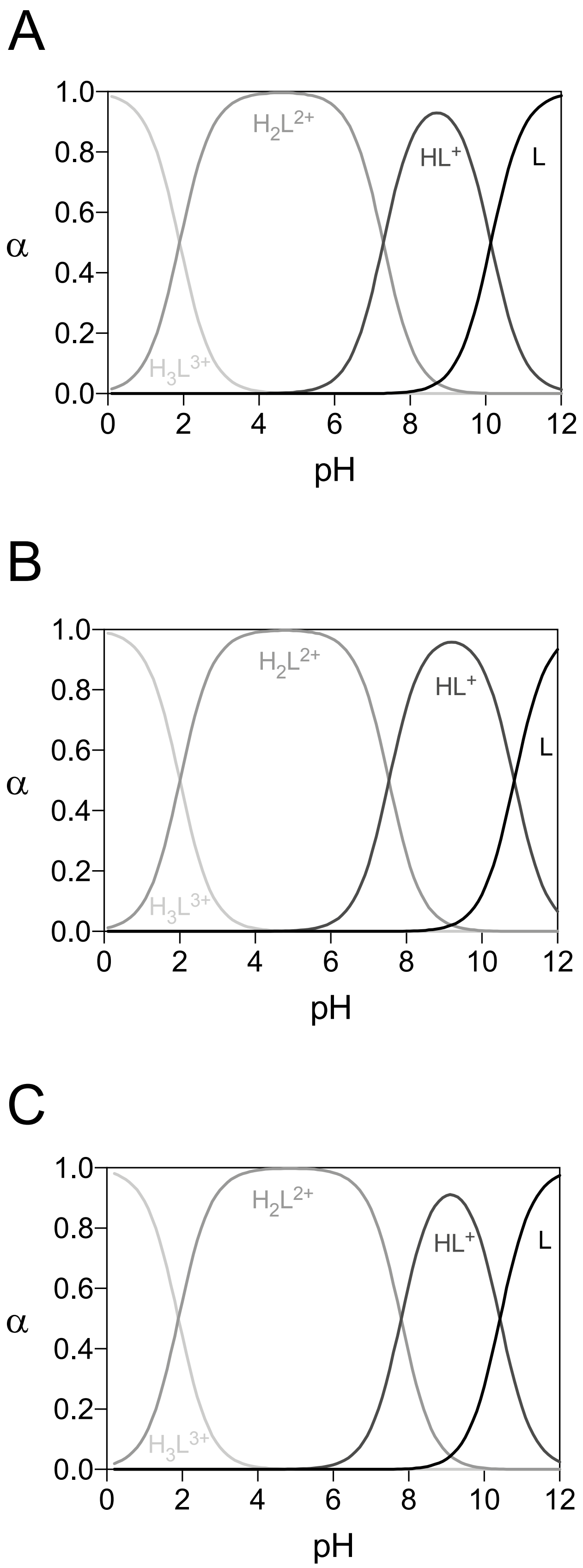


**Figure S2.** Distribution diagram of (A) DO4S, (B) DO3S and (C) DO3SAm, calculated using the protonation constants listed in **Table S1**.

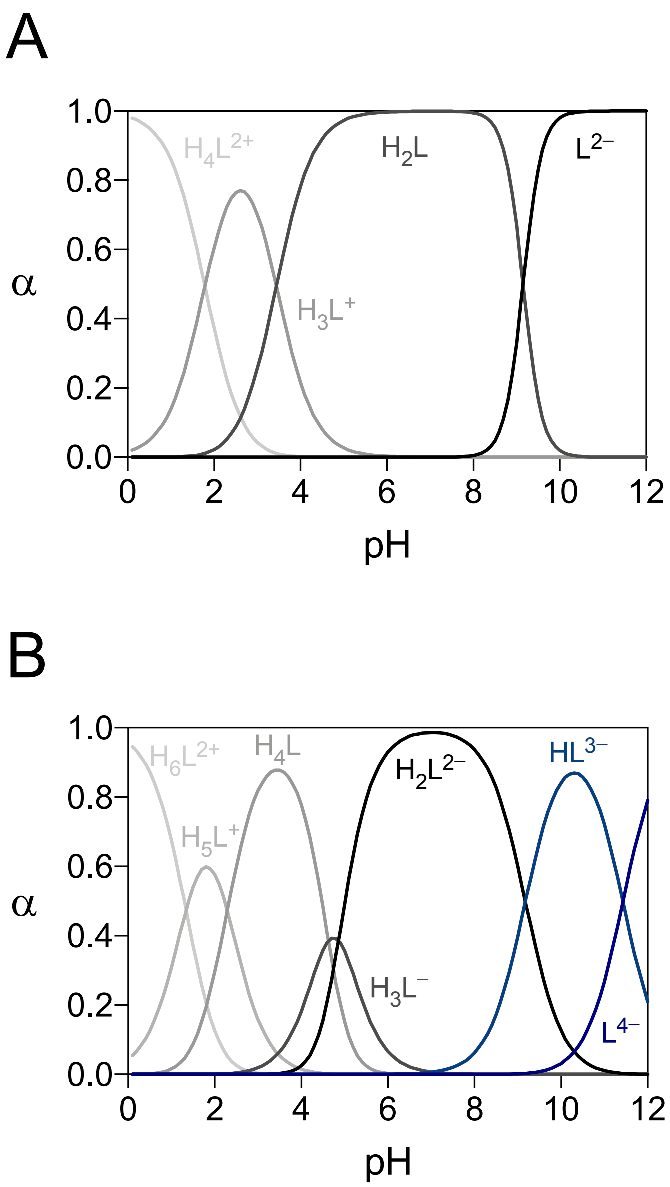


**Figure S3.** Distribution diagram of (A) DO2A2S and (B) DOTA calculated using the protonation constants listed in **Table S1**.

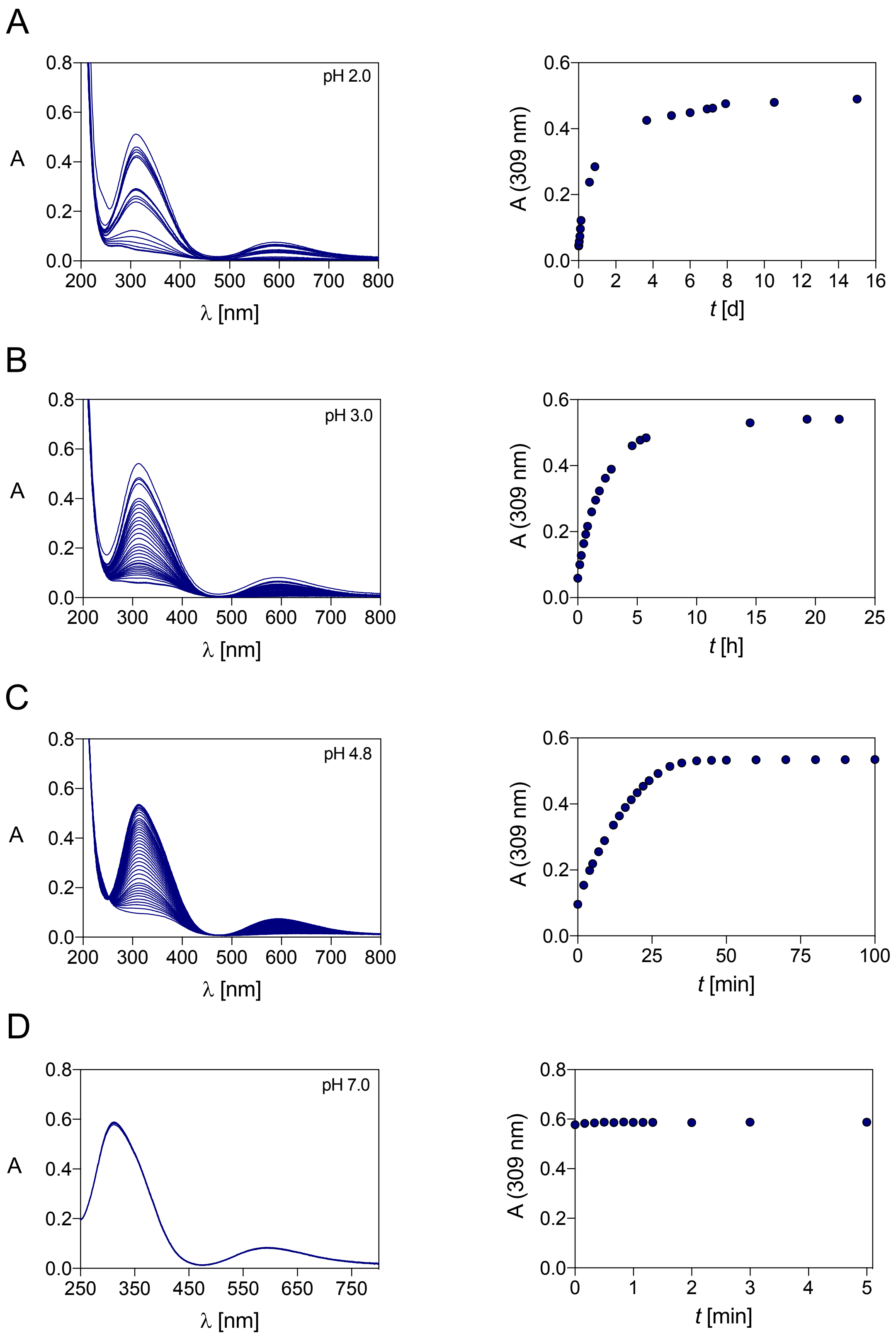


**Figure S4.** UV-Vis spectra (left) and time course (right) of the $Cu^{2+}$-DO4S complex formation at (A) pH 2.0 ($C_{DO4S} = C_{Cu^{2+}} = 1.2 \cdot 10^{-4}$ mol/L), (B) pH 3.0 ($C_{DO4S} = C_{Cu^{2+}} = 1.5 \cdot 10^{-4}$ mol/L), (C) pH 4.8 ($C_{DO4S} = C_{Cu^{2+}} = 1.3 \cdot 10^{-4}$ mol/L) and (D) pH 7.0 ($C_{DO4S} = C_{Cu^{2+}} = 1.5 \cdot 10^{-4}$ mol/L).

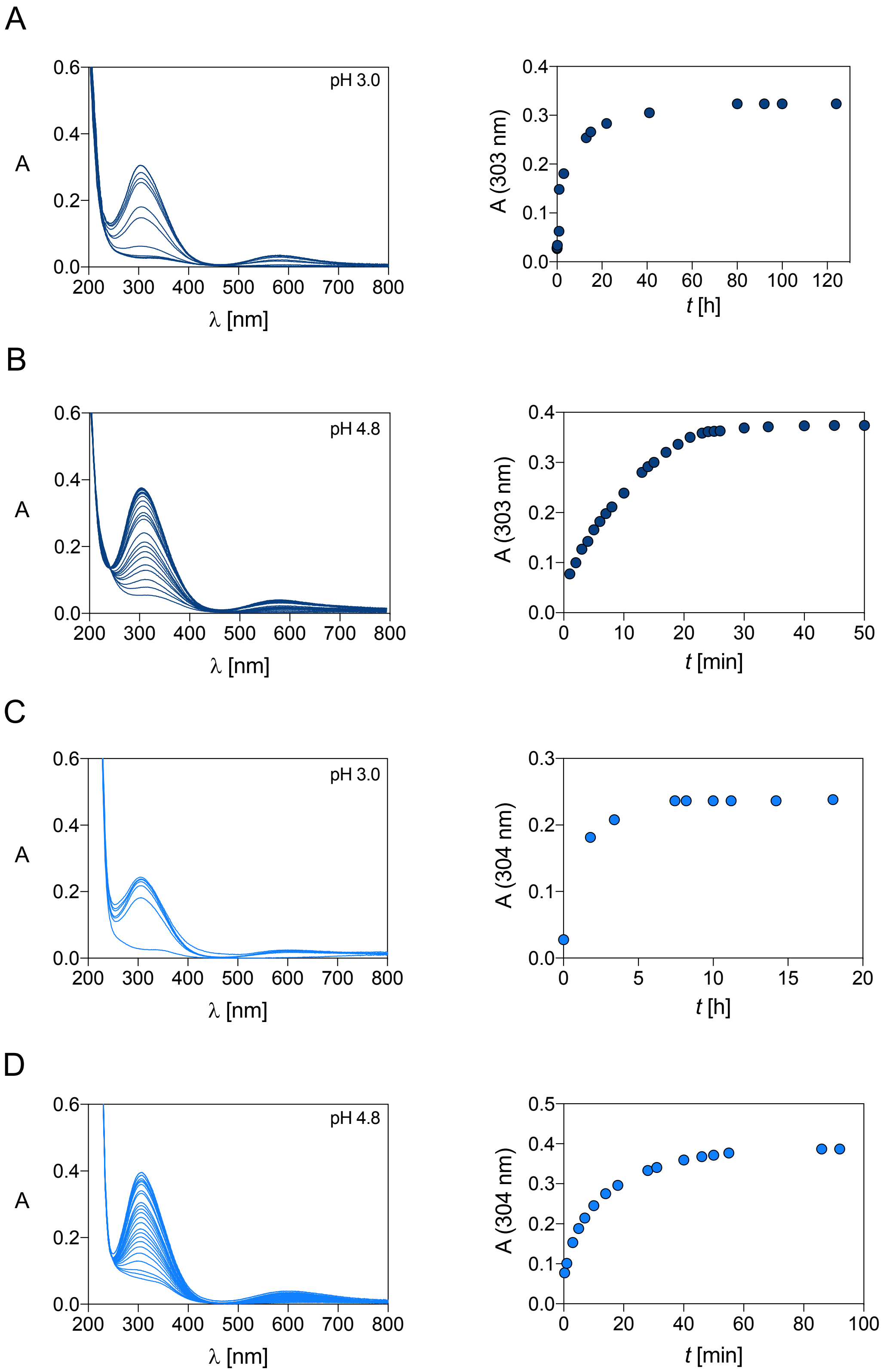


**Figure S5.** UV-Vis spectra (left) and time course (right) of the $Cu^{2+}$ complex formation with: (A) DO3S at pH 3.0 ($C_{DO3S}$ = $C_{Cu^{2+}}$ = 1.0·10$^{-4}$ mol/L); (B) DO3S at pH 4.8 ($C_{DO3S}$ = $C_{Cu^{2+}}$ = 1.2·10$^{-4}$ mol/L); (C) DO3SAm at pH 3.0 ($C_{DO3SAm}$ = $C_{Cu^{2+}}$ = 0.8·10$^{-4}$ mol/L); (D) DO3SAm at pH 4.8 ($C_{DO3SAm}$ = $C_{Cu^{2+}}$ = 1.1·10$^{-4}$ mol/L).

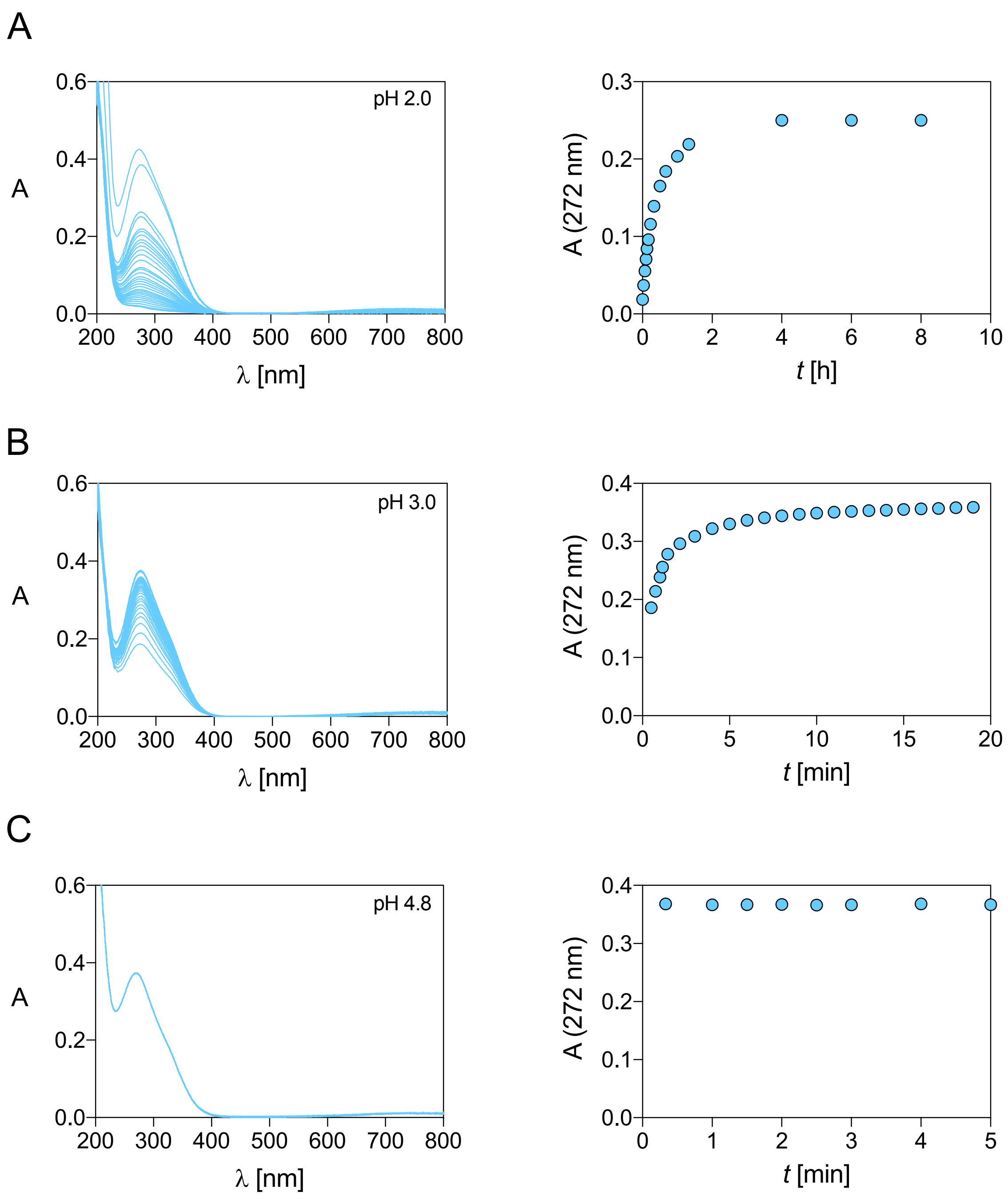


**Figure S6.** UV-Vis spectra (left) and time course (right) of the $Cu^{2+}$-DO2A2S complex formation at (A) pH 2.0 ($C_{DO2A2S} = C_{Cu^{2+}} = 0.8 \cdot 10^{-4}$ mol/L), (B) pH 3.0 ($C_{DO2A2S} = C_{Cu^{2+}} = 0.8 \cdot 10^{-4}$ mol/L) and (C) pH 4.8 ($C_{DO2A2S} = C_{Cu^{2+}} = 1.1 \cdot 10^{-4}$ mol/L).

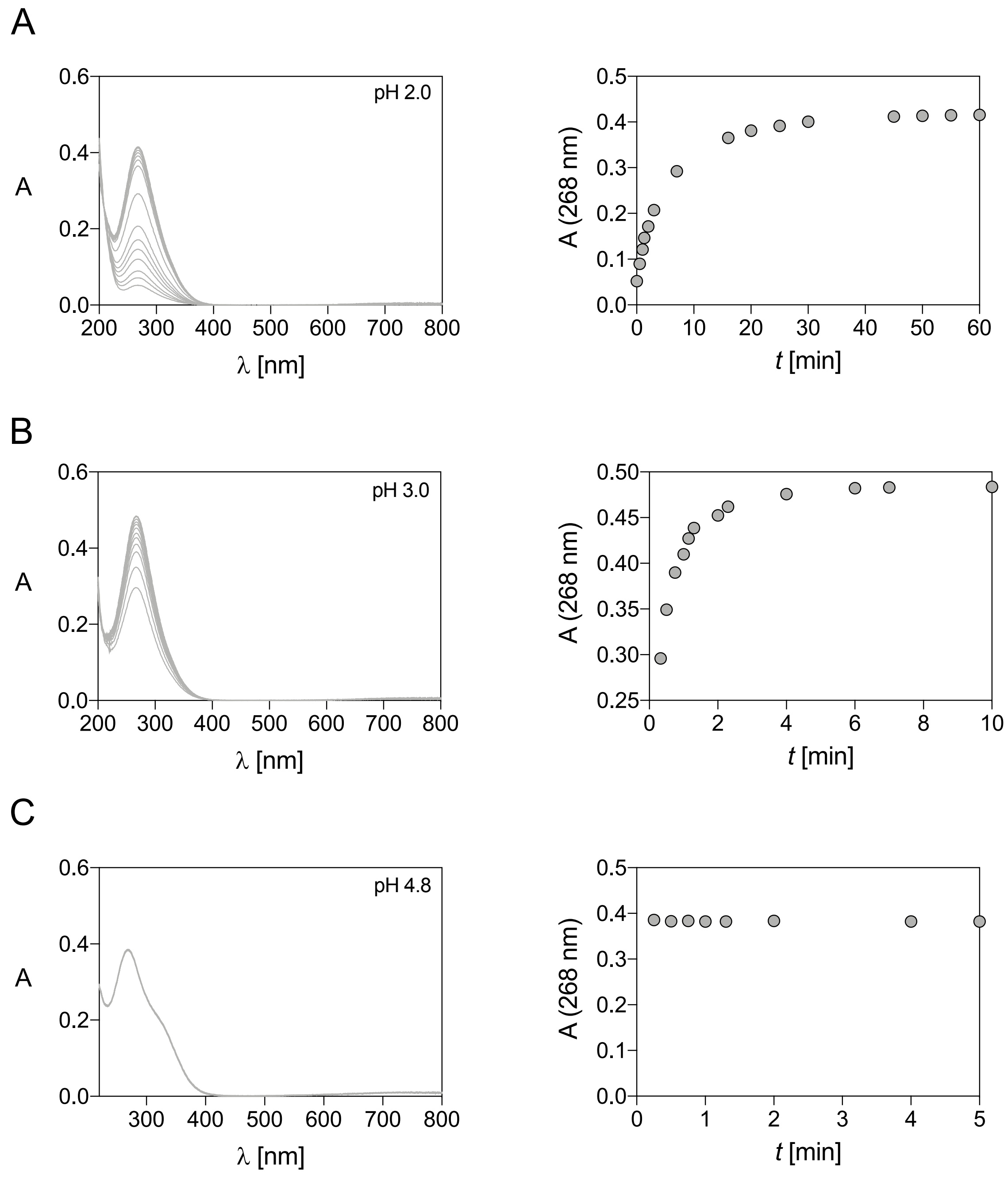


**Figure S7.** UV-Vis spectra (left) and time course (right) of the $Cu^{2+}$-DOTA complex formation at (A) pH 2.0 ($C_{DOTA} = C_{Cu^{2+}} = 1.0 \cdot 10^{-4}$ mol/L), (B) pH 3.0 ($C_{DOTA} = C_{Cu^{2+}} = 1.0 \cdot 10^{-4}$ mol/L) and (C) pH 4.8 ($C_{DOTA} = C_{Cu^{2+}} = 1.0 \cdot 10^{-4}$ mol/L).

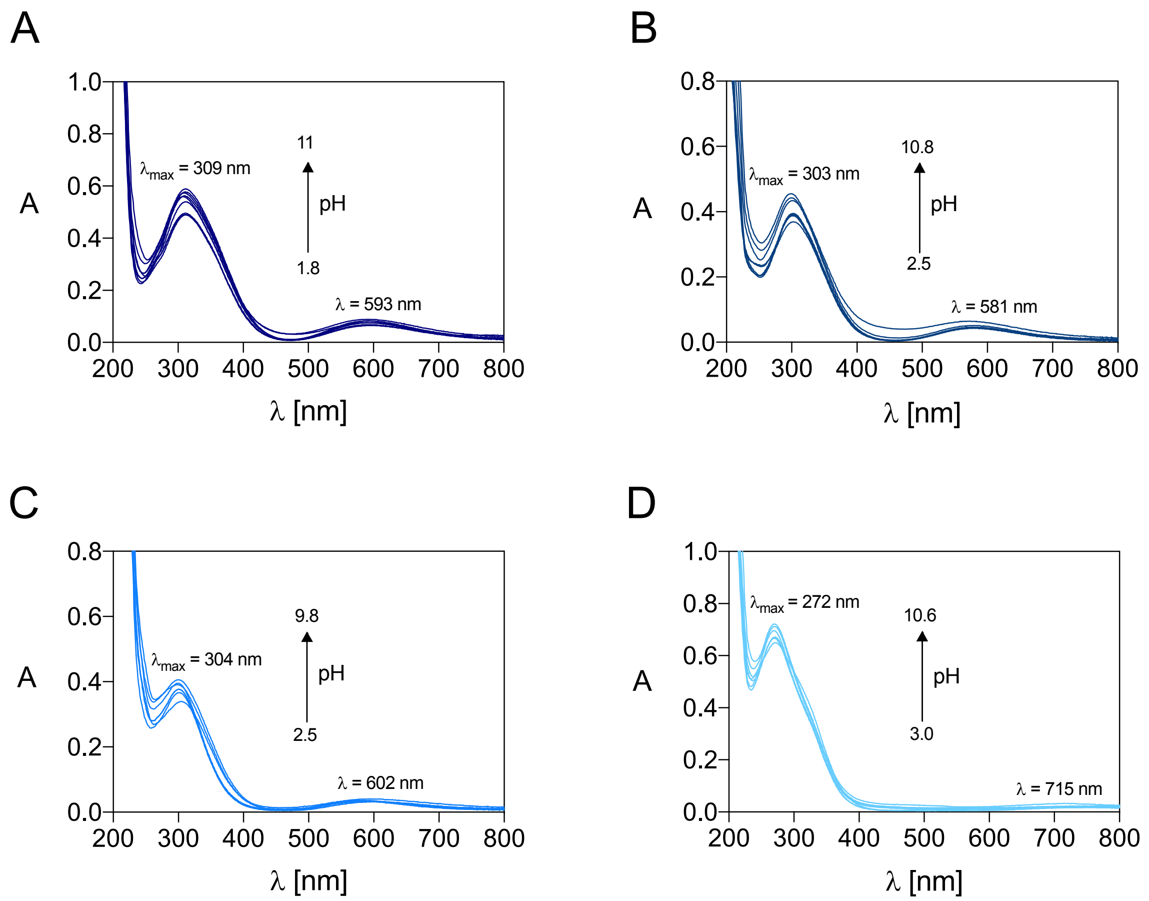


**Figure S8.** Selected UV-Vis spectra at pH > 2 of the $Cu^{2+}$ complexes with (A) DO4S ($C_{Cu^{2+}}$ = $C_{DO4S}$ = $1.5\cdot10^{-4}$ mol/L), (B) DO3S ($C_{Cu^{2+}}$ = $C_{DO3S}$ = $1.1\cdot10^{-4}$ mol/L), (C) DO3SAm ($C_{Cu^{2+}}$ = $C_{DO3SAm}$ = $1.0\cdot10^{-4}$ mol/L) and (D) DO2A2S ($C_{Cu^{2+}}$ = $C_{DO2A2S}$ = $1.4\cdot10^{-4}$ mol/L) at $I$ = 0.15 mol/L NaCl and $T$ = 25.0°C.

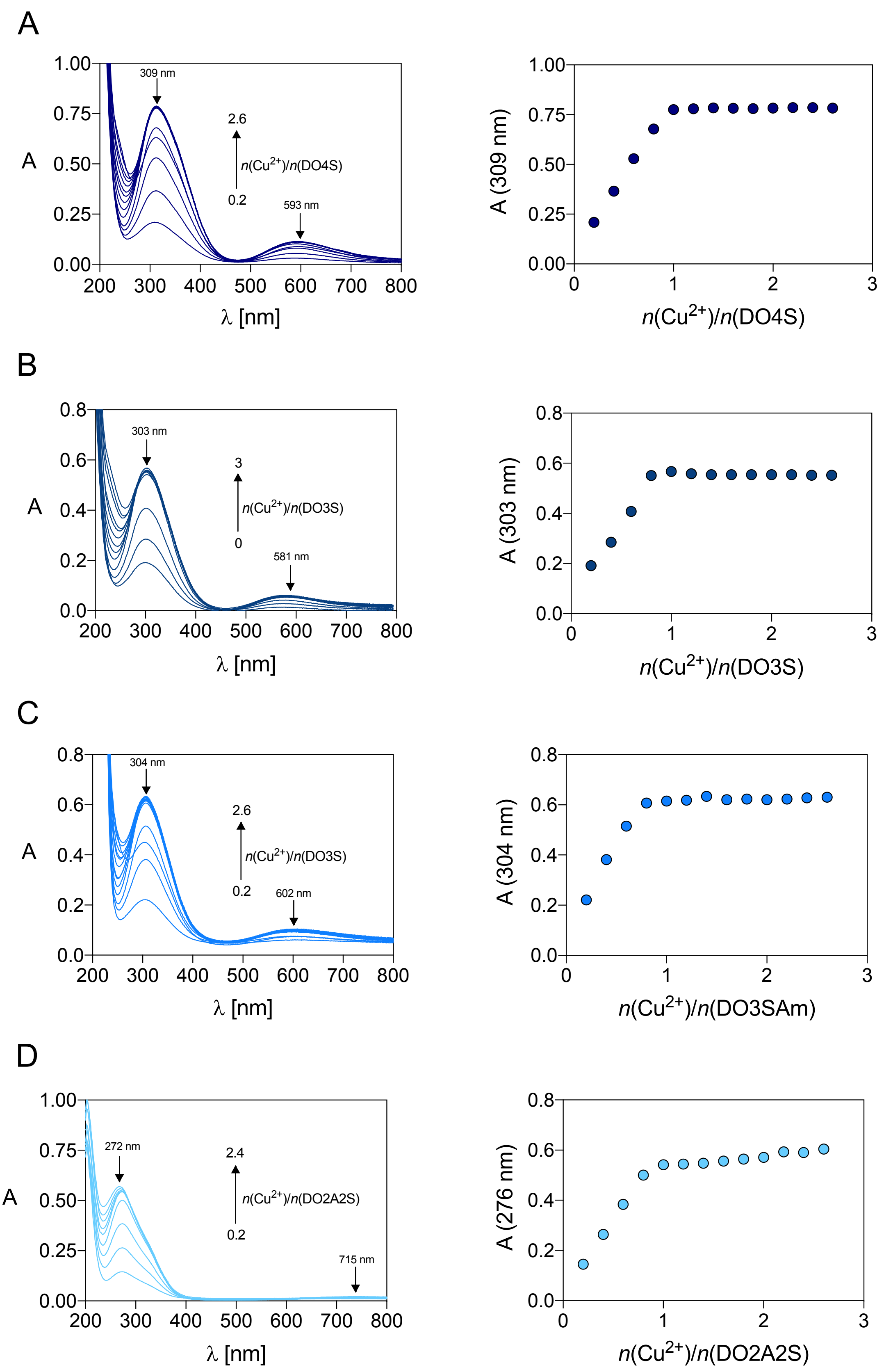


**Figure S9.** UV-Vis titrations by $Cu^{2+}$ of solutions at pH 4.8 containing (A) DO4S ($C_{DO4S}$ = $2.0 \cdot 10^{-4}$ mol/L), (B) DO3S ($C_{DO3S}$ = $1.6 \cdot 10^{-4}$ mol/L), (C) DO3SAm ($C_{DO3SAm}$ = $1.6 \cdot 10^{-4}$ mol/L) and (D) DO2A2S ($C_{DO2A2S}$ = $1.2 \cdot 10^{-4}$ mol/L).

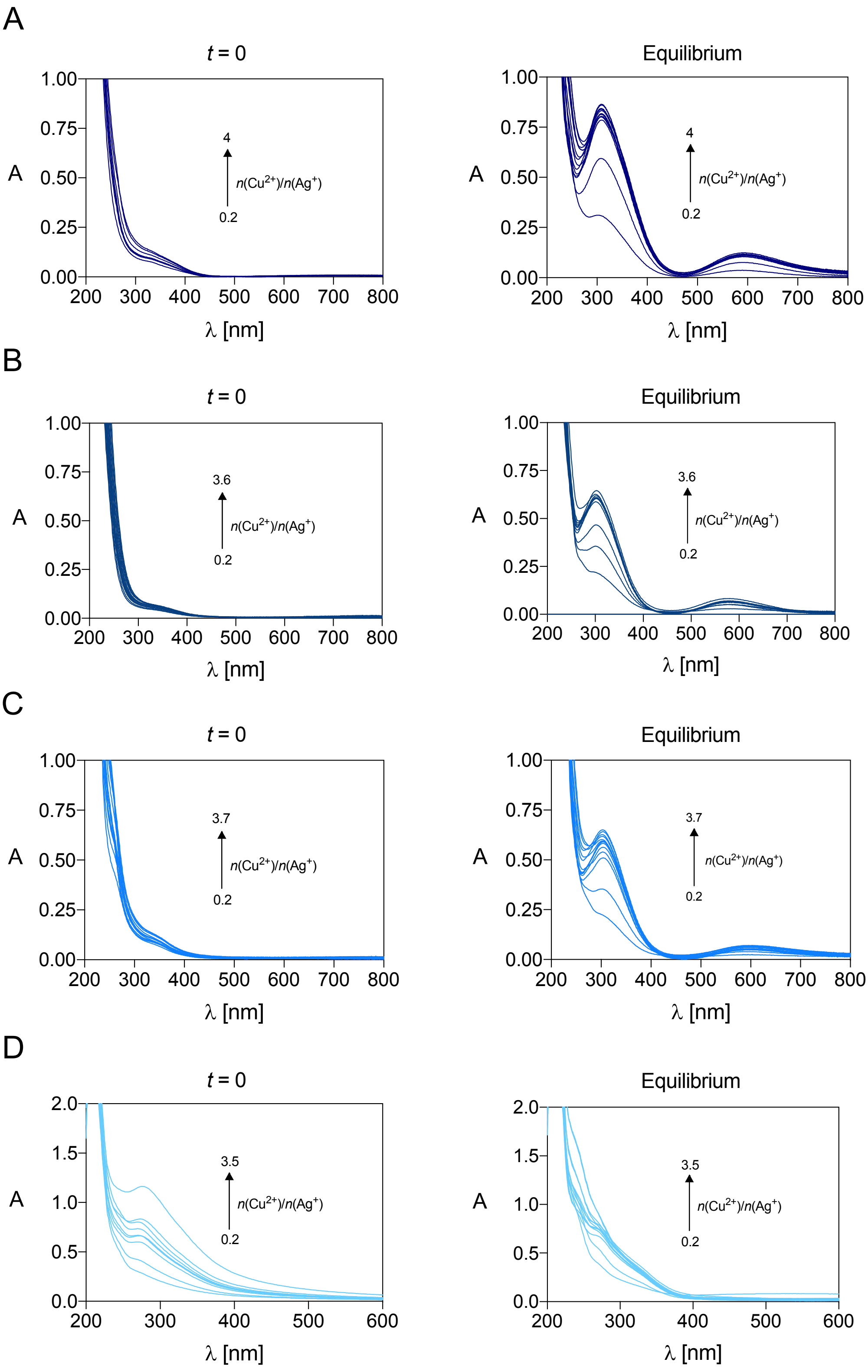


**Figure S10.** UV-Vis spectra at pH 4.8 of the preformed $Ag^+$ complex with (A) DO4S ($C_{Ag^+}$ = $C_{DO4S}$ = 2.2·10$^{-4}$ mol/L), (B) DO3S ($C_{Ag^+}$ = $C_{DO3S}$ = 1.5·10$^{-4}$ mol/L), (C) DO3SAm ($C_{Ag^+}$ = $C_{DO4S}$ = 1.6·10$^{-4}$ mol/L) and (D) DO2A2S ($C_{Ag^+}$ = $C_{DO4S}$ = 1.2·10$^{-4}$ mol/L) immediately after the addition of variable equivalents of $Cu^{2+}$ ($t$ = 0) and at equilibrium during the competition titrations.

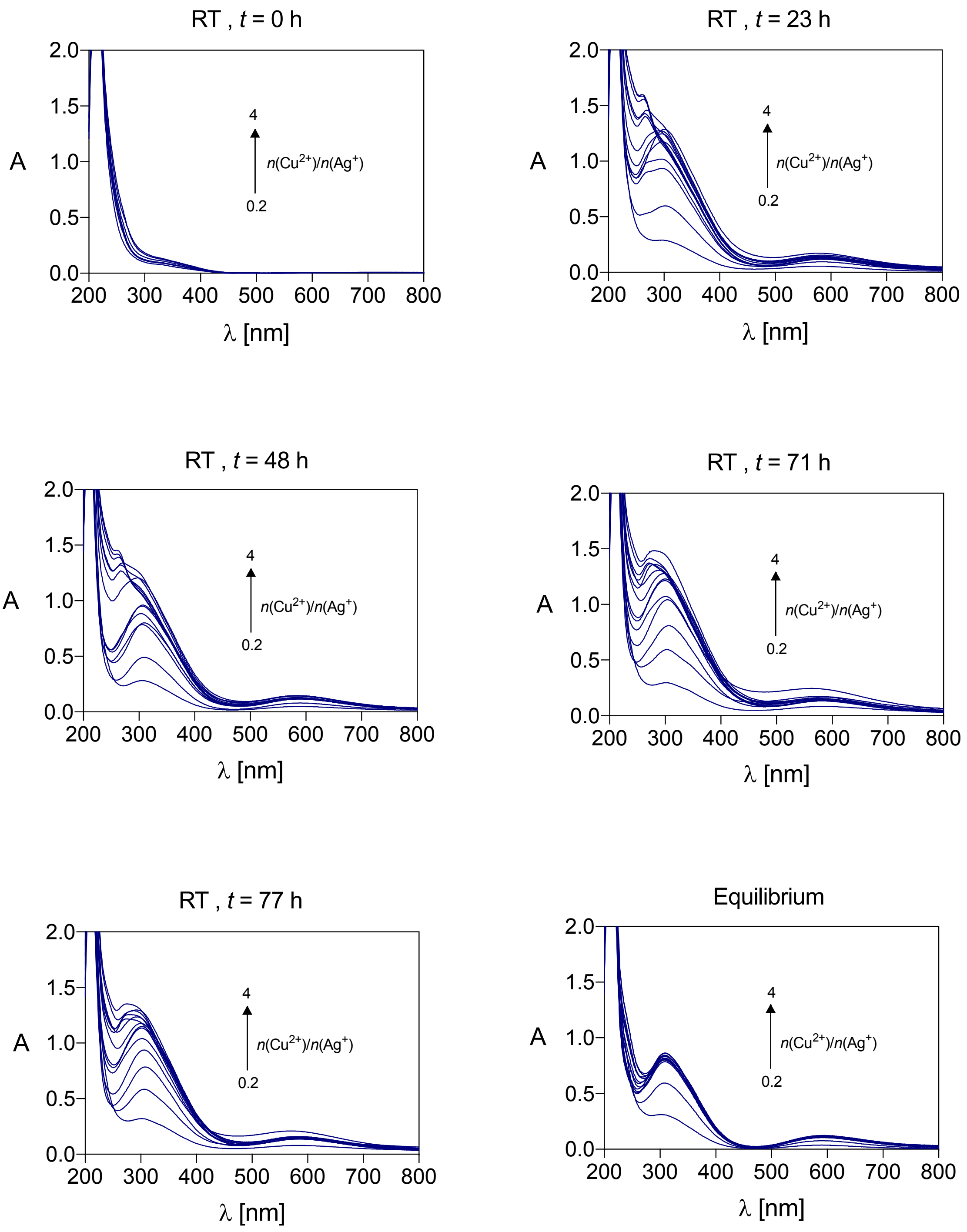


**Figure S11.** Changes in the UV-Vis spectra over time at pH 4.8 of the preformed $Ag^{+}$ complex with DO4S ($C_{Ag^{+}} = C_{DO4S} = 2.2 \cdot 10^{-4}$ mol/L) during the $Cu^{2+}$ competition titrations.

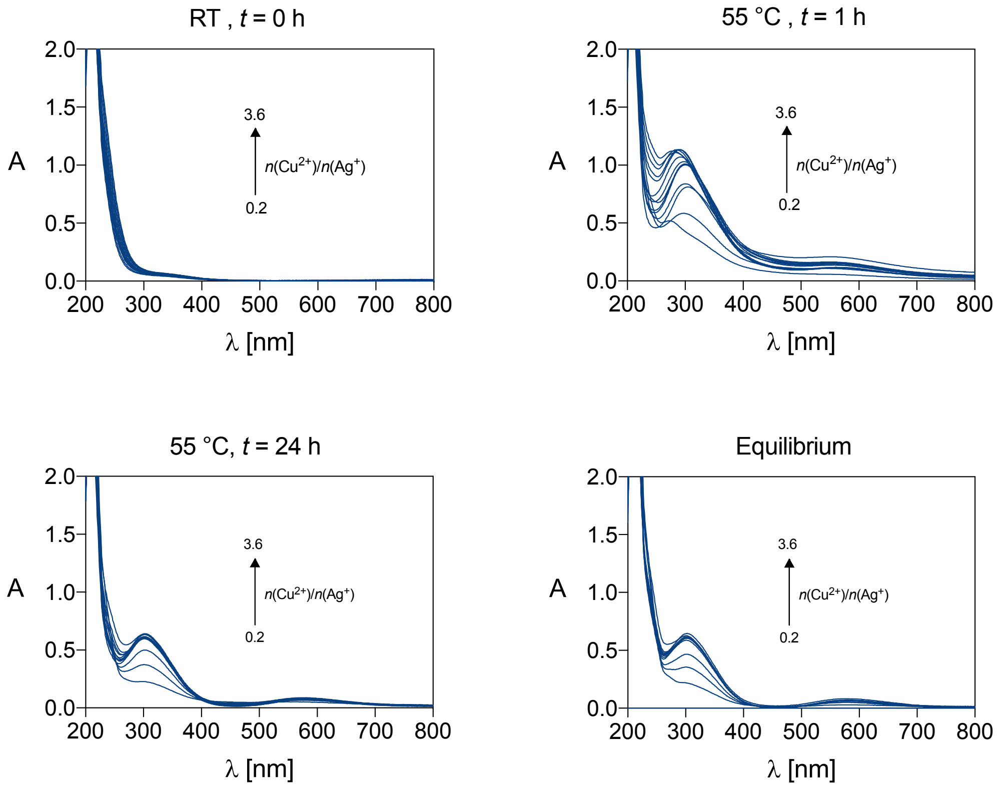


**Figure S12.** Changes in the UV-Vis spectra over time at pH 4.8 of the preformed $Ag^+$ complex with DO3S ($C_{Ag^+}$ = $C_{DO3S}$ = 1.5·10$^{-4}$ mol/L) during the $Cu^{2+}$ competition titrations.

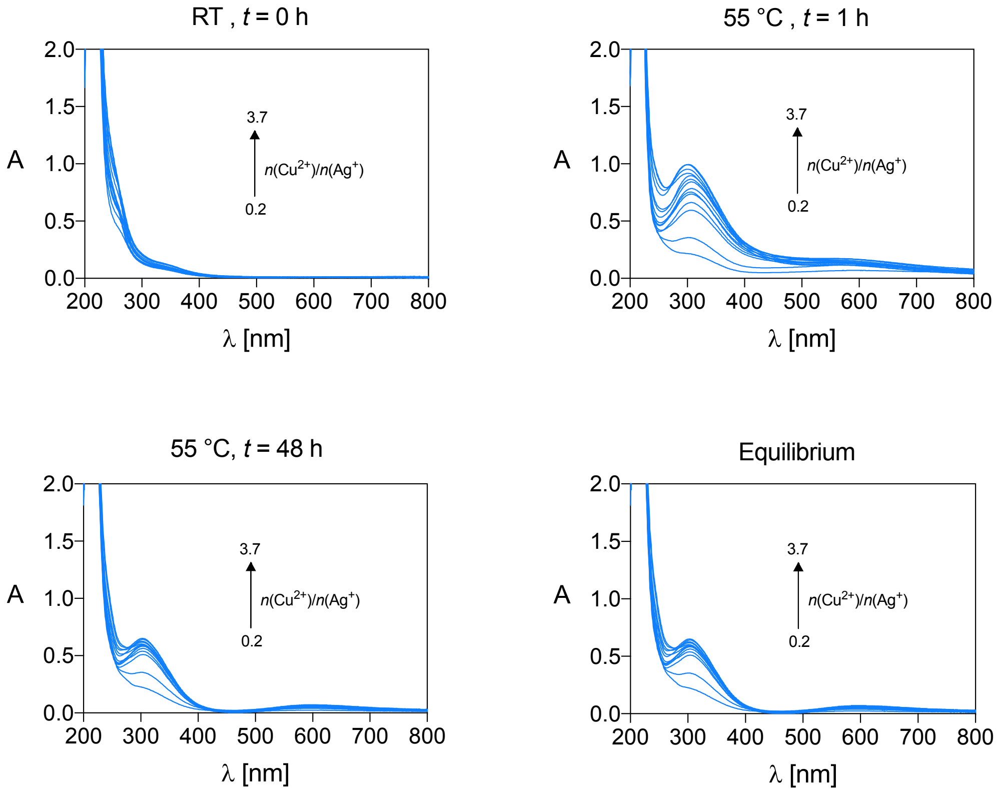


**Figure S13.** Changes in the UV-Vis spectra over time at pH 4.8 of the preformed $Ag^+$ complex with DO3SAm ($C_{Ag^+}$ = $C_{DO3SAm}$ = 1.6·10$^{-4}$ mol/L) during the $Cu^{2+}$ competition titrations.

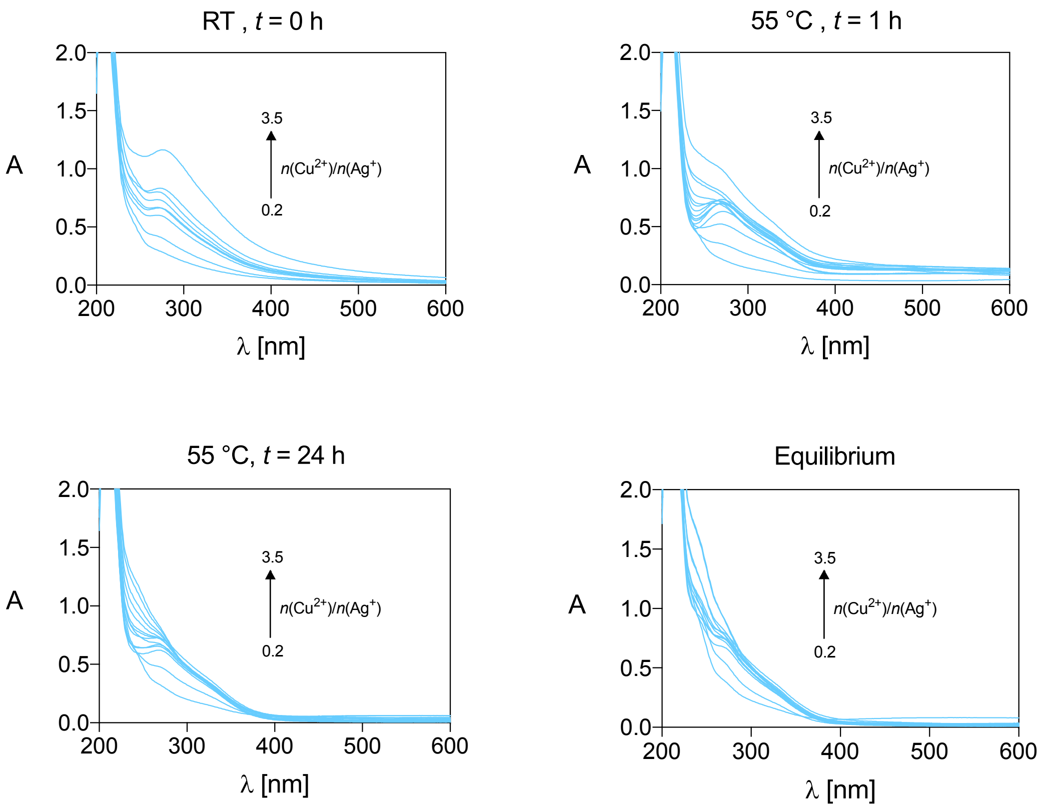


**Figure S14.** Changes in the UV-Vis spectra over time at pH 4.8 of the preformed $Ag^+$ complex with DO2A2S ($C_{Ag^+}$ = $C_{DO2A2S}$ = 1.2·10$^{-4}$ mol/L) during the $Cu^{2+}$ competition titrations.

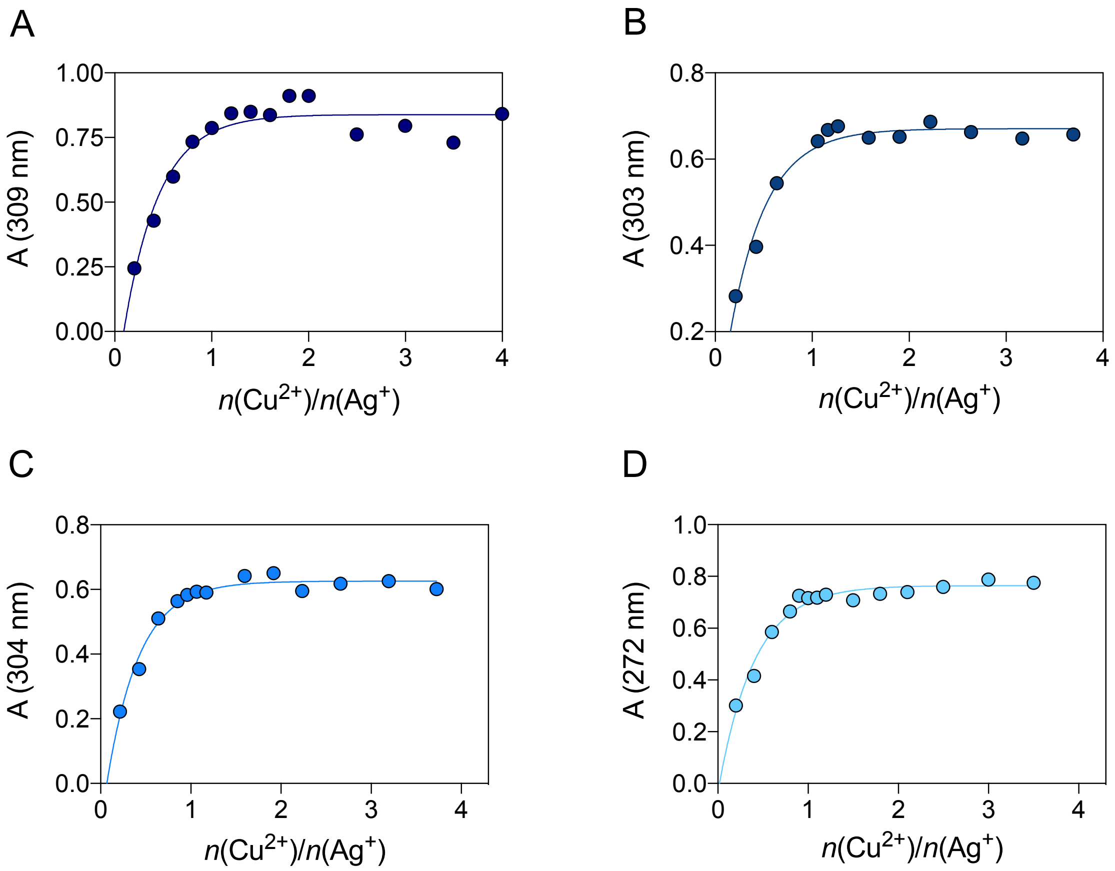


**Figure S15.** Representative *A* vs. *n*($Cu^{2+}$)/n($Ag^{+}$) profiles obtained during the $Ag^{+}$-$Cu^{2+}$ competitions measurements (see **Figure S10**) and corresponding fitting lines.

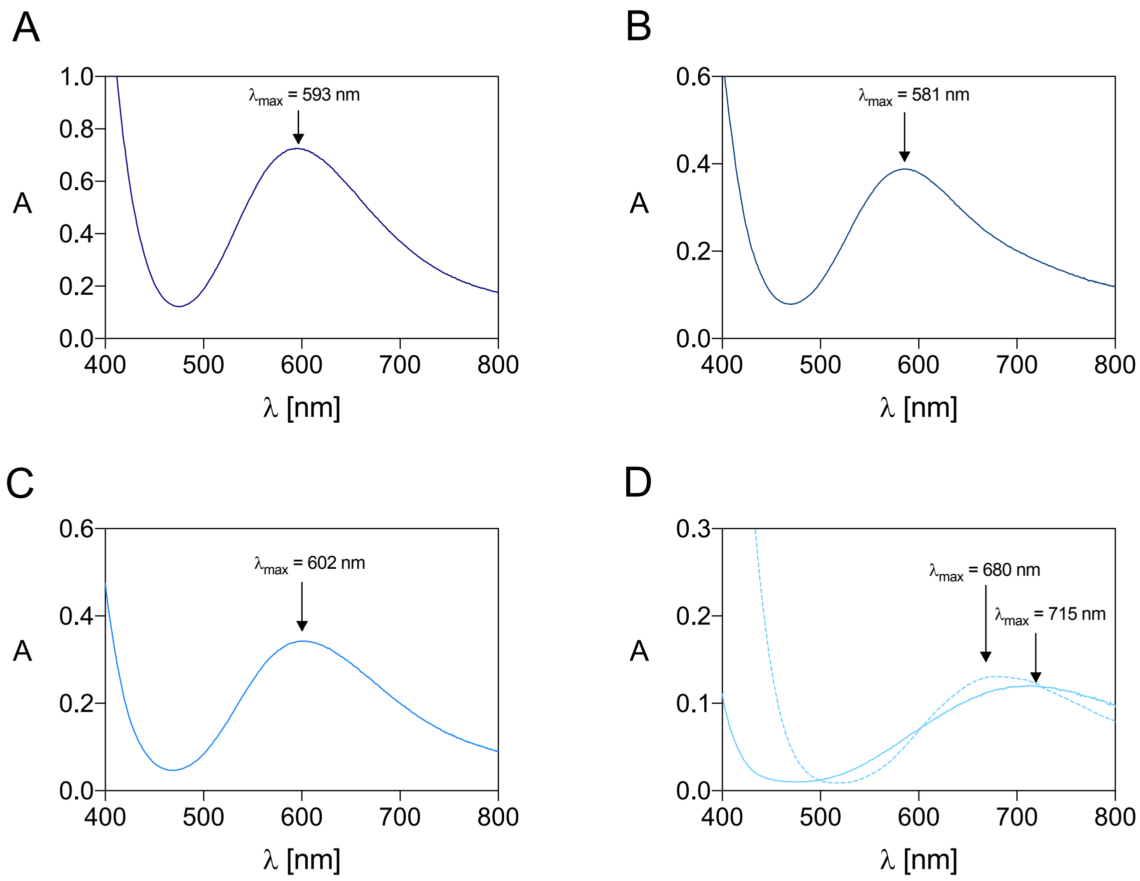


**Figure S16.** *d-d* band of the cupric complexes with (A) DO4S ($C_{Cu^{2+}}$ = $C_{DO4S}$ = 1.2·10$^{-3}$ mol/L), (B) DO3S ($C_{Cu^{2+}}$ = $C_{DO3S}$ = 9.2·10$^{-4}$ mol/L), (C) DO3SAm ($C_{Cu^{2+}}$ = $C_{DO3SAm}$ = 1.1·10$^{-3}$ mol/L) and (D) DO2A2S (pH 2.0, $C_{Cu^{2+}}$ = $C_{DO2A2S}$ = 7.8·10$^{-4}$ mol/L; pH 1.8 (dotted line), $C_{Cu^{2+}}$ = $C_{DO2A2S}$ = 9.0·10$^{-4}$ mol/L) at $I$ = 0.15 mol/L NaCl and $T$ = 25.0 °C.

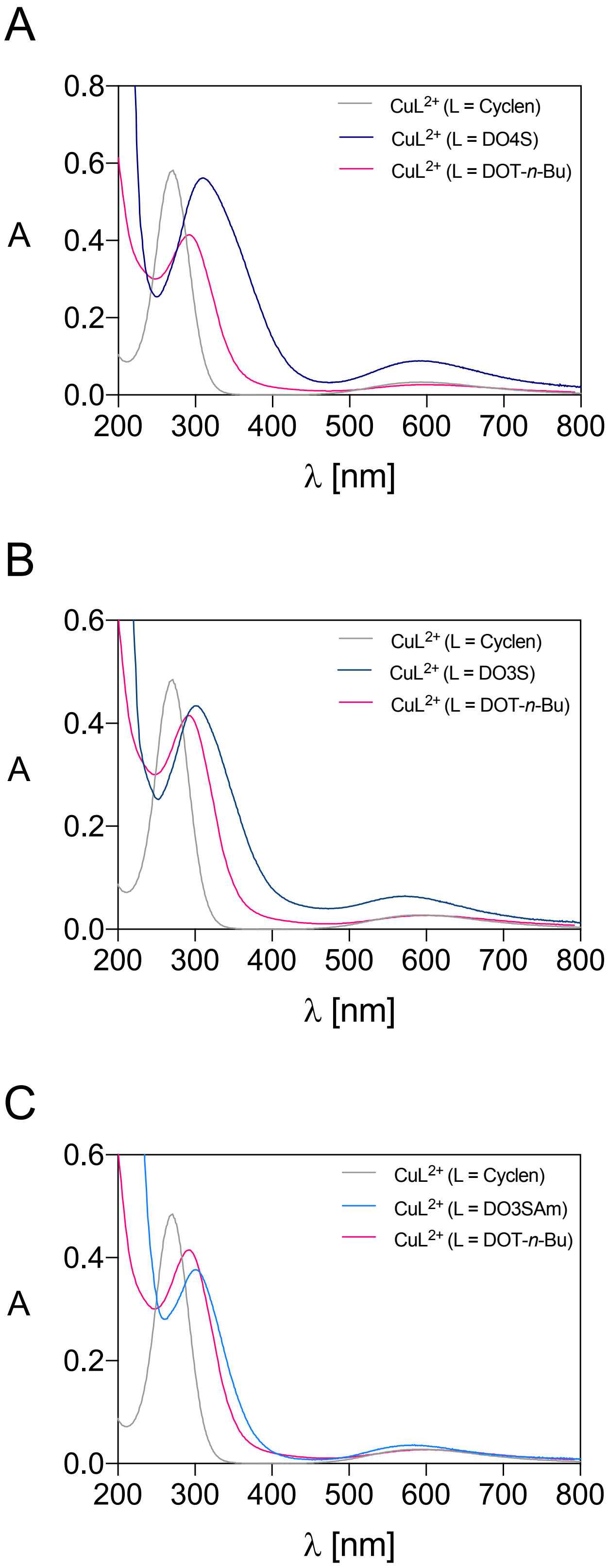


**Figure S17.** Comparison of the electronic spectra of the $CuL^{2+}$ complexes formed by (A) DO4S, (B) DO3S and (C) DO3SAm (data from **Figure S8**) with those of the same complex formed by cyclen ($C_{Cu^{2+}}$ = $C_{cyclen}$ = $1.5 \cdot 10^{-4}$ mol/L) and by DOT-*n*-Bu ($C_{Cu^{2+}}$ = $C_{DOT\text{-}n\text{-}tBu}$ = $2.1 \cdot 10^{-4}$ mol/L). The $Cu^{2+}$-cyclen spectra obtained in this work agree with the literature data.[5,6]

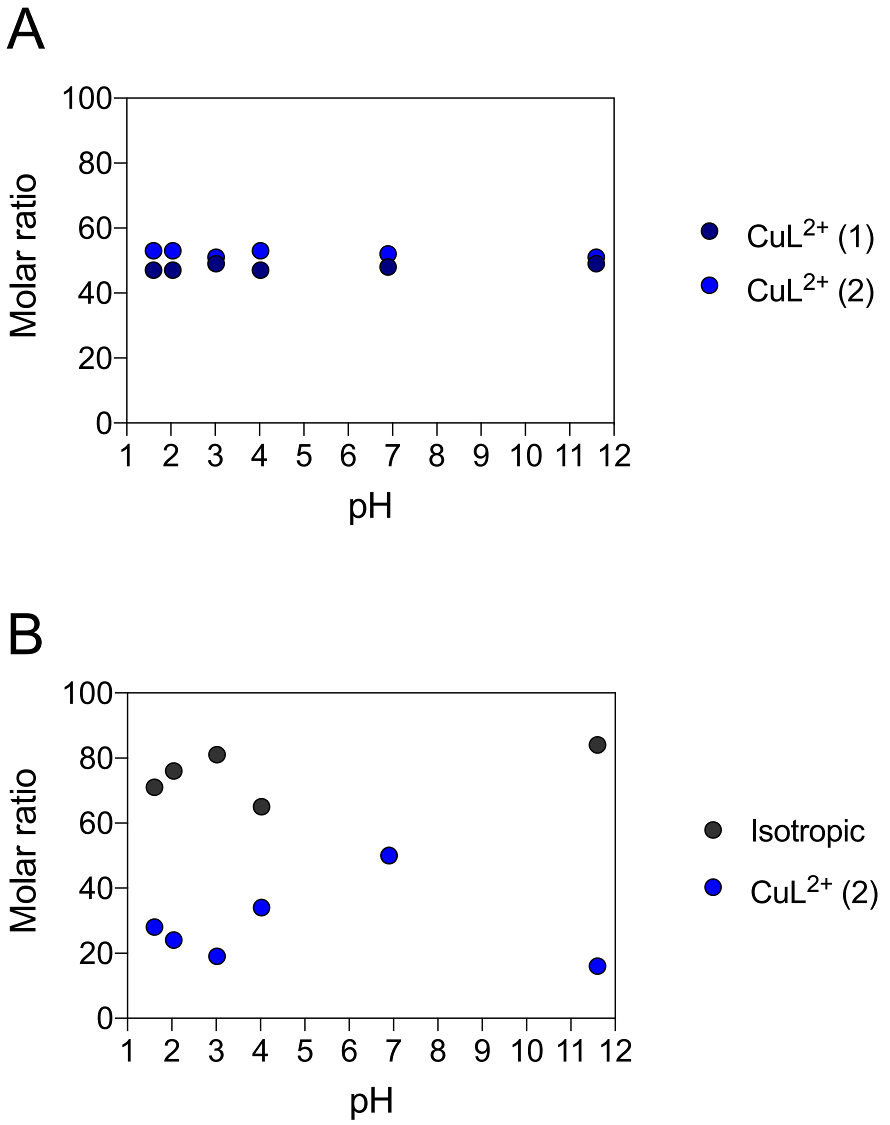


**Figure S18.** Component ratios obtained from the simulation of $Cu^{2+}$-DO4S EPR spectra recorded at (A) room temperature and (B) 77 K.

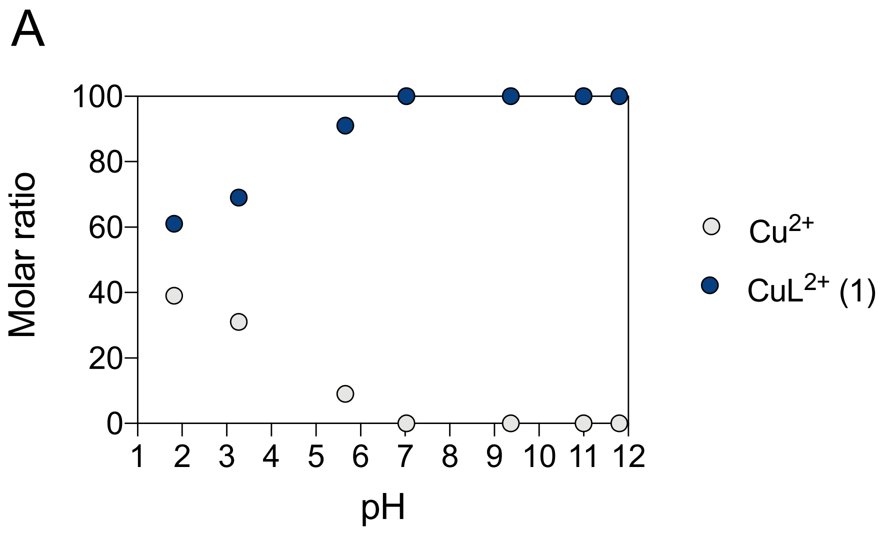


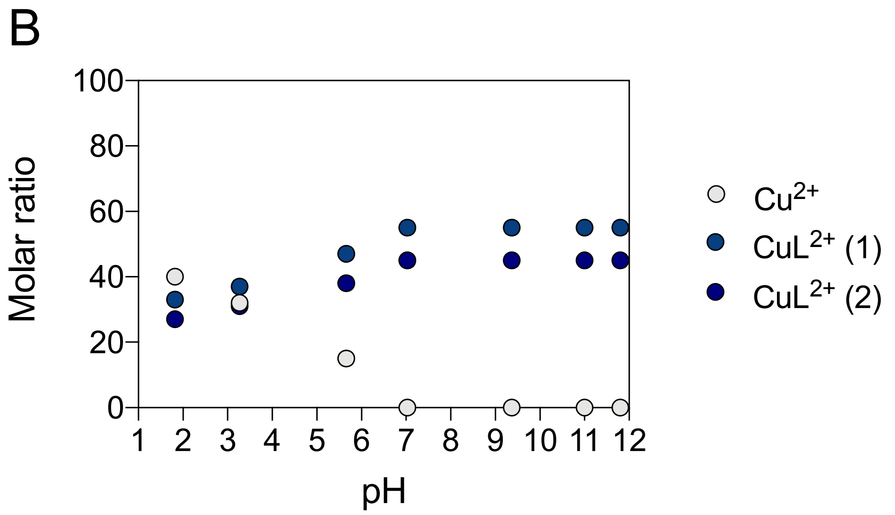


**Figure S19.** Component ratios obtained from the simulation of $Cu^{2+}$-DO3S EPR spectra recorded at (A) room temperature and (B) 77 K.

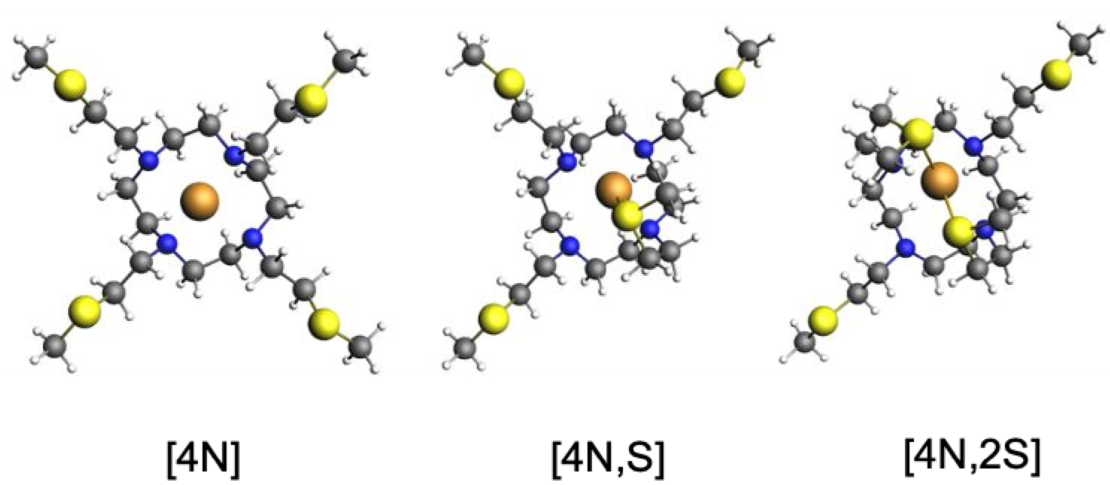


**Figure S20.** DFT-examined isomers of the $CuL^{2+}$ complex formed by DO4S.

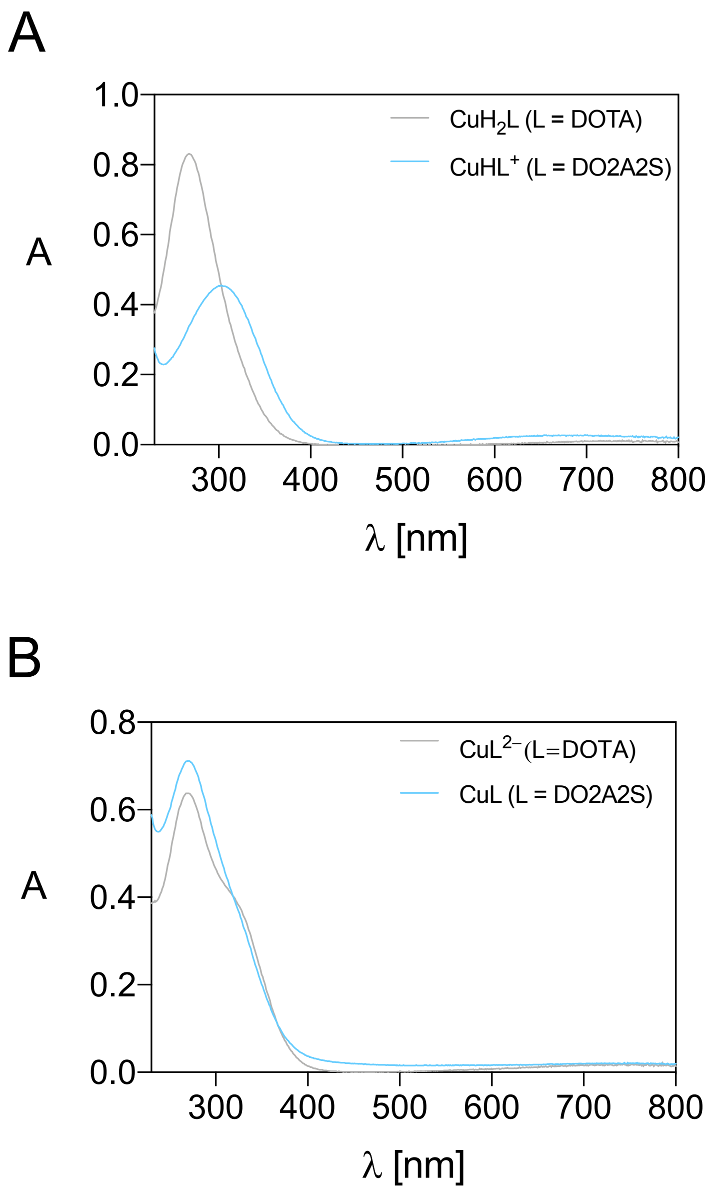


**Figure S21.** Comparison of the UV-Vis spectra of (A) $CuHL^+$ for DO2A2S (data from **Figure 2**) and $CuH_2L$ for DOTA ($C_{Cu^{2+}} = C_{DOTA} = 2.0 \cdot 10^{-4}$ mol/L); (B) CuL for DO2A2S (data from **Figure S8**) and $CuL^{2-}$ for DOTA ($C_{Cu^{2+}} = C_{DOTA} = 2.0 \cdot 10^{-4}$ mol/L). The $Cu^{2+}$-DOTA spectra obtained in this work agree with the literature data.[7]

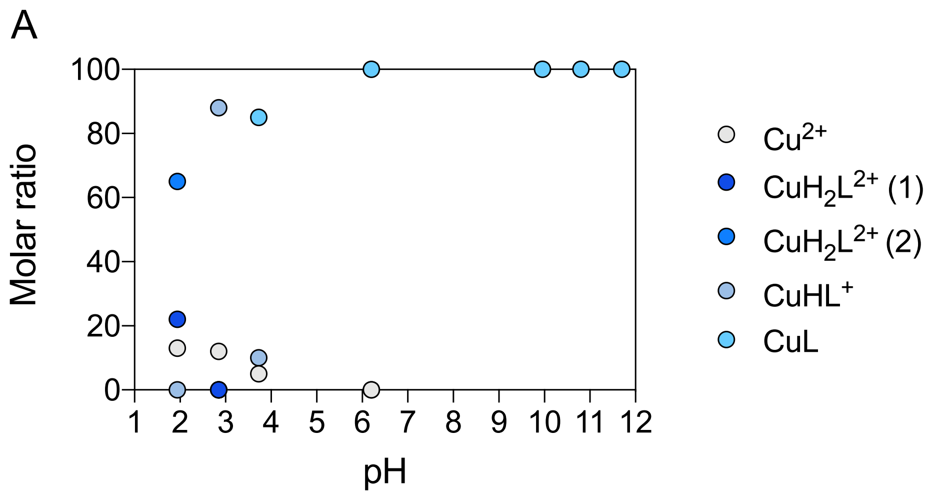


**Figure S22.** Component ratios obtained from the simulation of $Cu^{2+}$-DO2A2S EPR spectra recorded at 77 K.

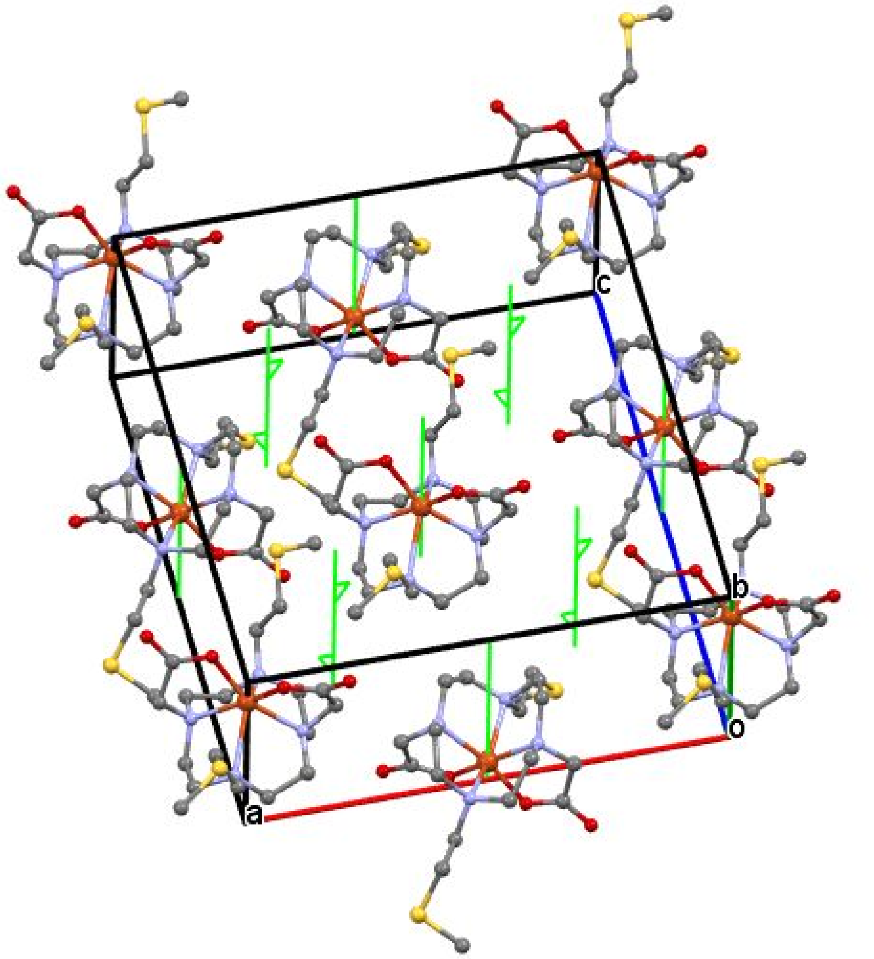


**Figure S23.** Unit cell of crystal [Cu(DO2A2S)] showing the 2-fold rotation and screw axes.

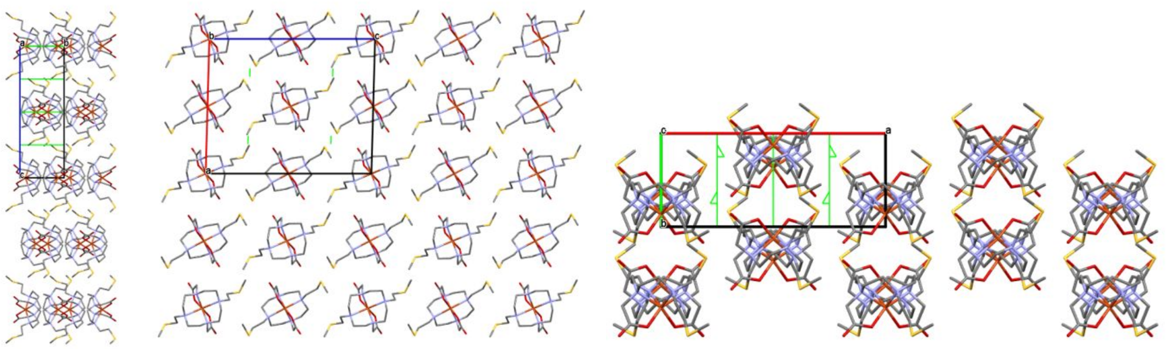

**Figure S24.** Packing arrangements in crystal [Cu(DO2A2S)] viewed from the crystallographic directions ’a’, ’b’ and ’c’.

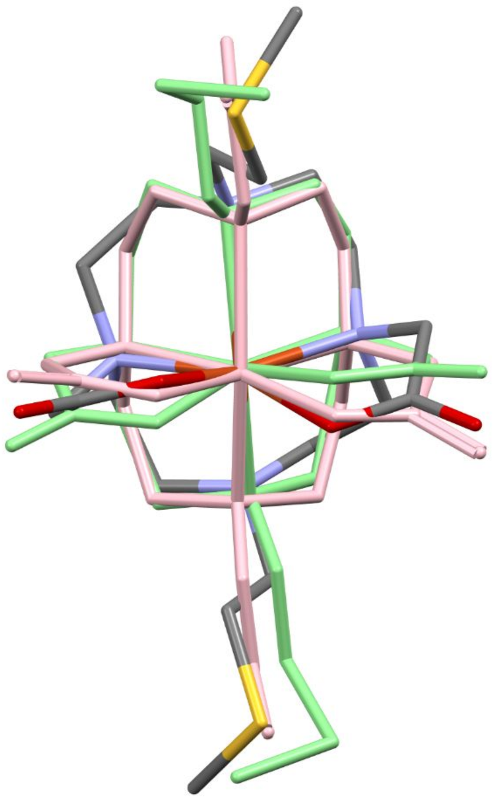

**Figure S25.** Comparison of the conformation of molecules in the asymmetrical unit of crystal [Cu(DO2A2S)] by overlay of the two molecules (molecule #1 is coloured by element, and #2 is green) together with crystal structure of Cu-DOTA (pink, Ref. Code FEKVAS).[8]

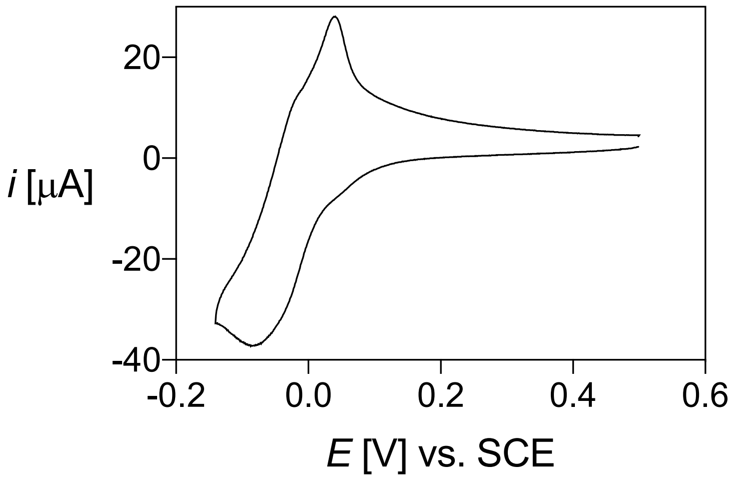


**Figure S26.** Cyclic voltammogram of unbound $Cu^{2+}$ ($C_{Cu^{2+}}$ = 2.3·10$^{-3}$ mol/L) in aqueous solution at pH 7, $I$ = 0.15 mol/L $NaNO_3$ and $T$ = 25 °C, acquired at a scan rate of 0.1 V/s.

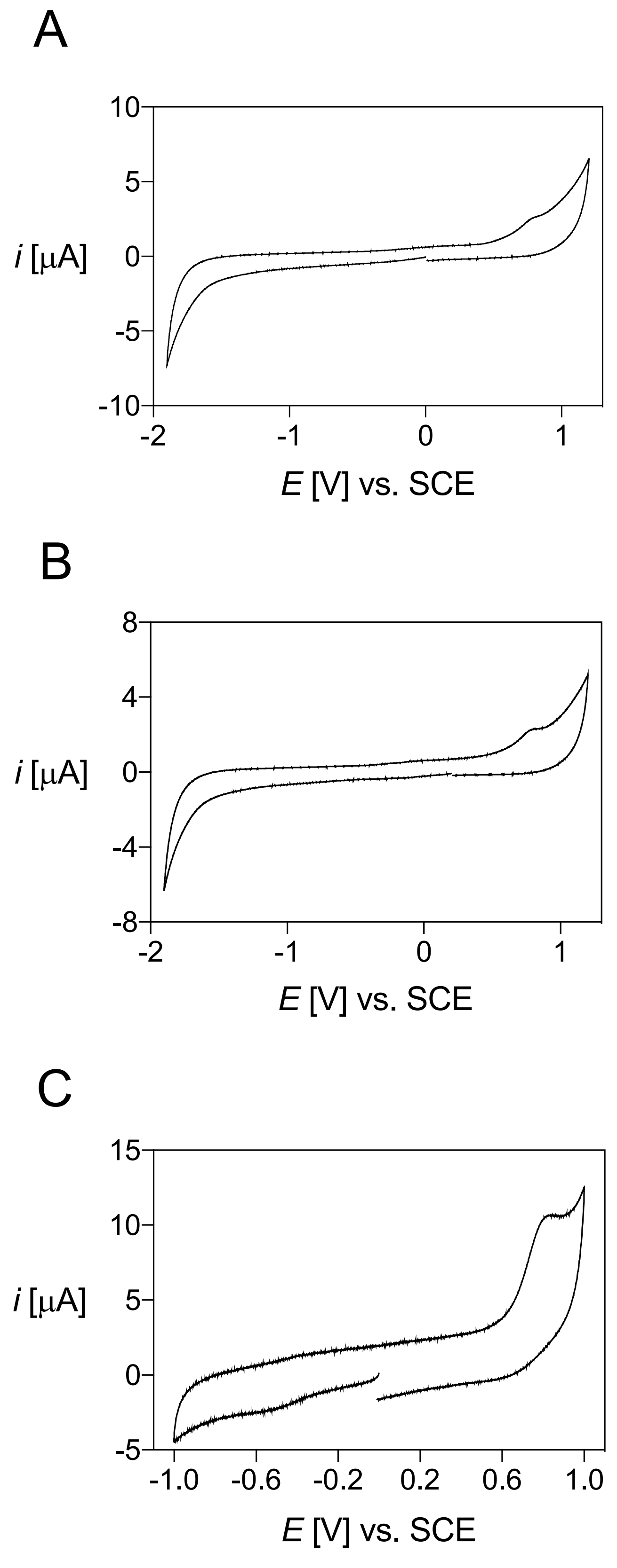


**Figure S27.** Cyclic voltammogram of (A) DO4S ($C_{DO4S}$ = 1.0·10$^{-3}$ mol/L), (B) DO3S ($C_{DO3S}$ = 1.1·10$^{-3}$ mol/L) and (C) DO2A2S ($C_{DO2A2S}$ = 1.0·10$^{-3}$ mol/L) in aqueous solution at pH 7, $I$ = 0.15 mol/L $NaNO_3$ and $T$ = 25 °C, acquired at a scan rate of 0.1 V/s.

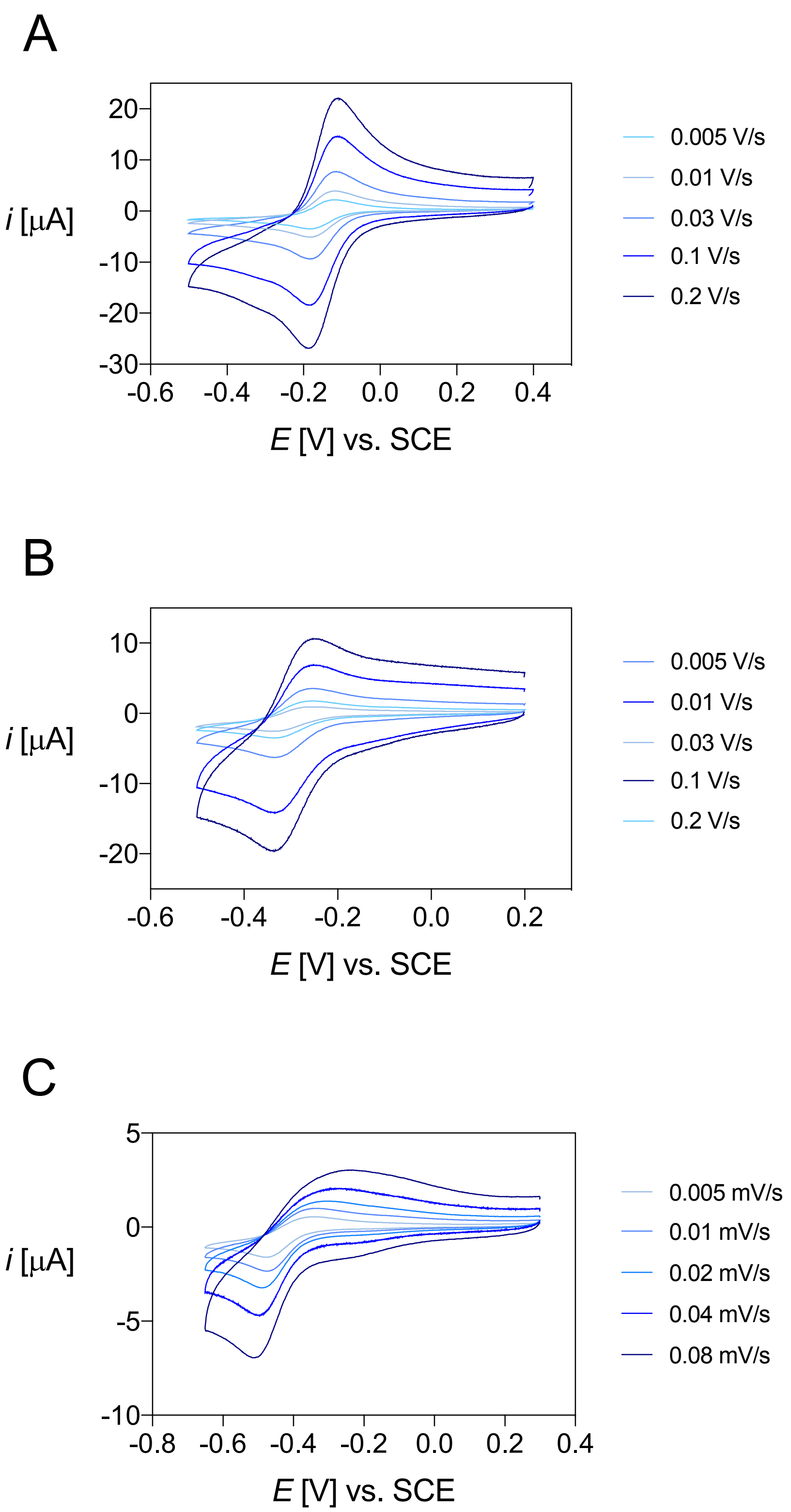


**Figure S28.** Cyclic voltammograms of copper complexes of (A) DO4S ($C_{CuL^{2+}}$ = $1.0\cdot10^{-3}$ mol/L), (B) DO3S ($C_{CuL^{2+}}$ = $1.1\cdot10^{-3}$ mol/L) and (C) DO2A2S ($C_{CuL^{2+}}$ = $6.48\cdot10^{-4}$ mol/L) in aqueous solution at pH 7, $I$ = 0.15 mol/L $NaNO_3$ and $T$ = 25 °C, acquired at different scan rates (0.005 - 0.2 V/s).

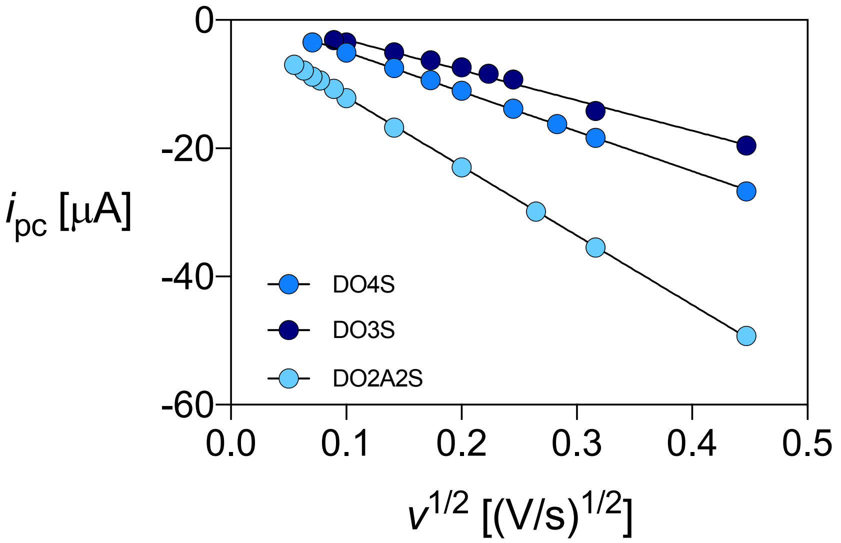


**Figure S29.** Variation of the cathodic current intensity ($i_{pc}$) with the square root of the scan speed ($v^{1/2}$) for the $Cu^{2+}$ complexes of (A) DO4S, (B) DO3S and (C) DO2A2S, and linear regressions.

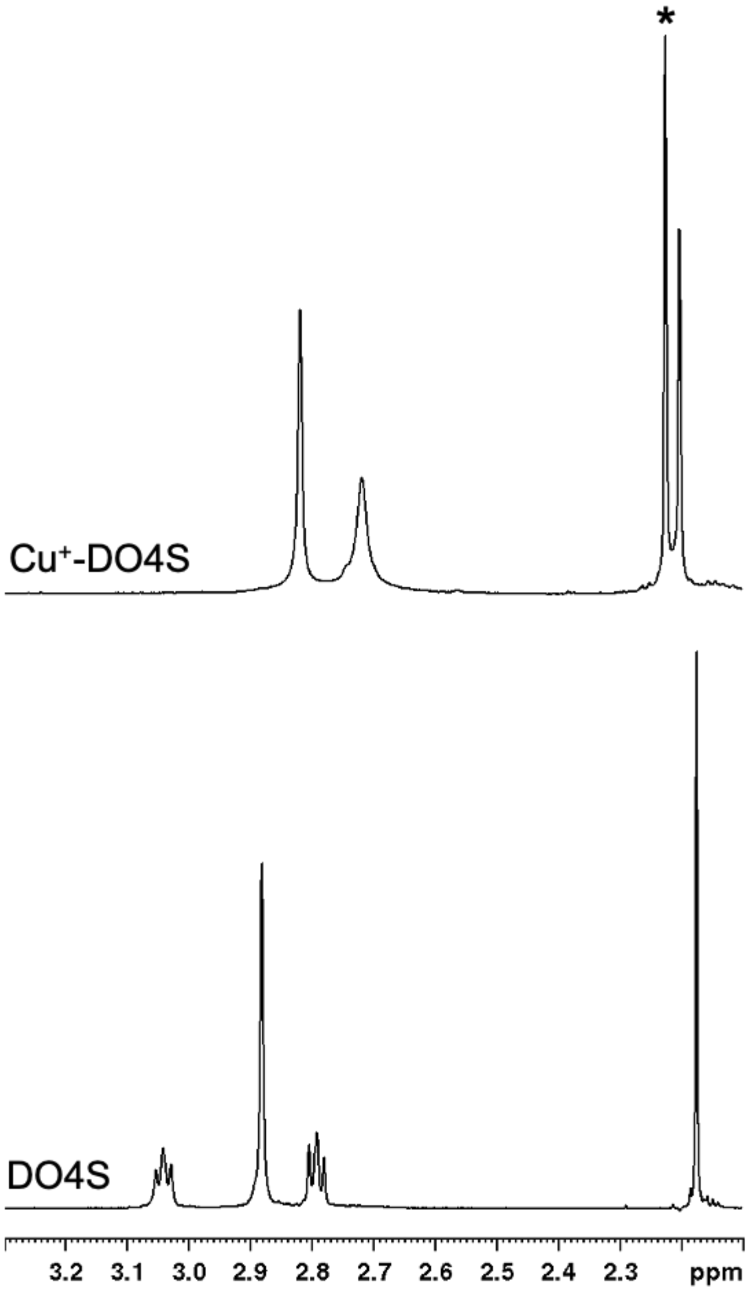


**Figure S30.** Comparison between the $^{1}$H-NMR spectra of $Cu^{+}$-DO4S (400 MHz, RT, $H_2O$ + 10% $D_2O$) at pH 7 (data from **Figure 9**) and free monoprotonated DO4S (600 MHz, RT, $D_2O$). Data for the latter were taken from our previous work.[1] The signal marked with an asterisk (2.22 ppm) is related to acetone impurity.

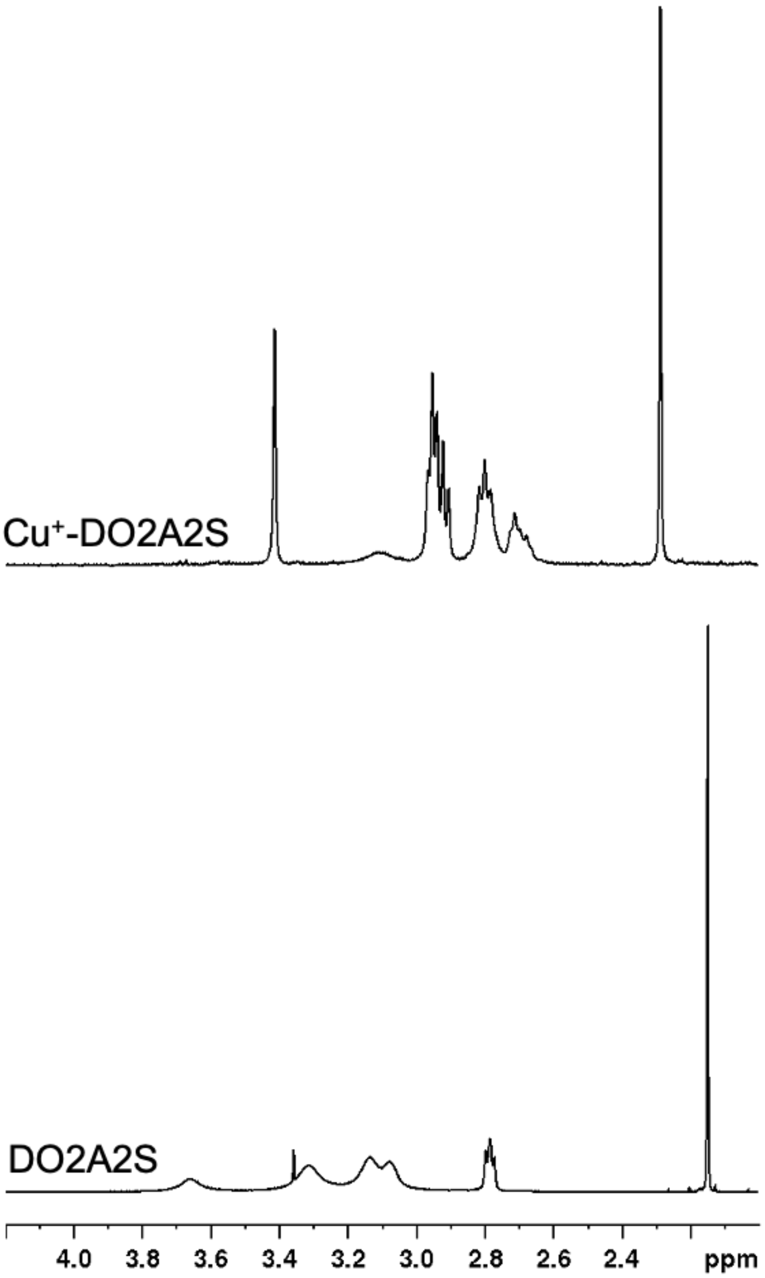


**Figure S31.** Comparison between the $^{1}$H-NMR spectra of $Cu^{+}$-DO2A2S (400 MHz, RT, $H_2O$ + 10% $D_2O$) at pH 7 (data from **Figure 9**) and free monoprotonated DO2A2S (600 MHz, RT, $D_2O$). Data for the latter were taken from our previous work.[1]

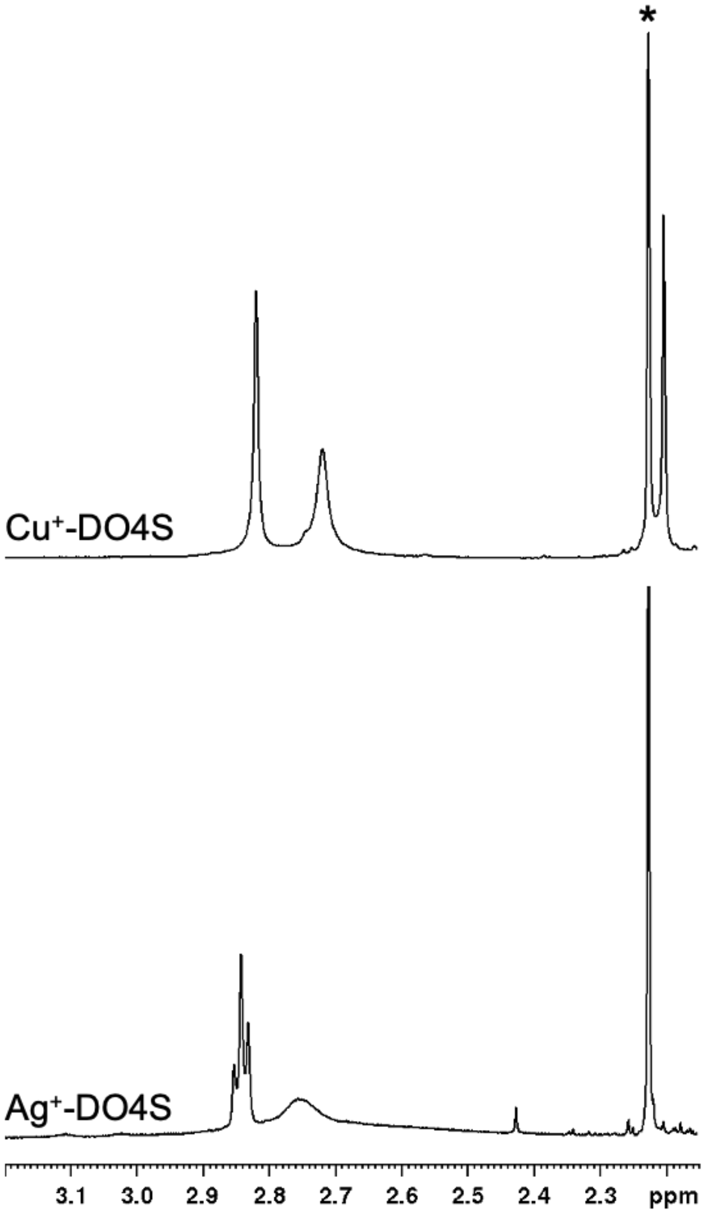


**Figure S32.** Comparison between the $^{1}$H-NMR spectra of $Cu^{+}$-DO4S (400 MHz, RT, $H_2O$ + 10% $D_2O$) (data from **Figure 9**) and of $Ag^{+}$-DO4S solutions (600 MHz, RT, $D_2O$) at pH 7. Data for the latter were taken from our previous work.[9] The signal marked with an asterisk (2.22 ppm) is related to acetone impurity.

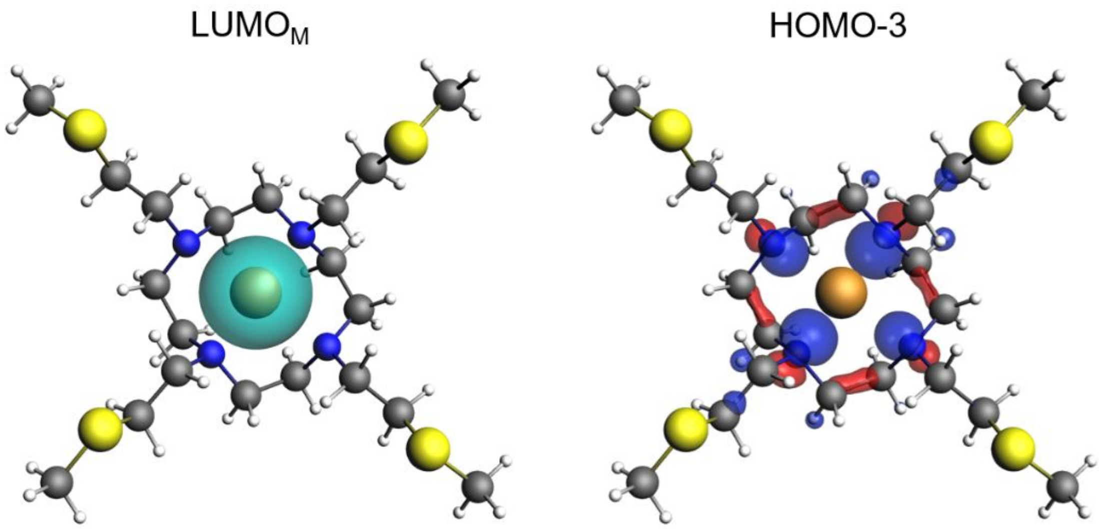


**Figure S33.** Main interacting symmetry-adapted fragment orbitals (SFOs) of the $Cu^+$-DO4S complex displaying a [4N] coordination mode.

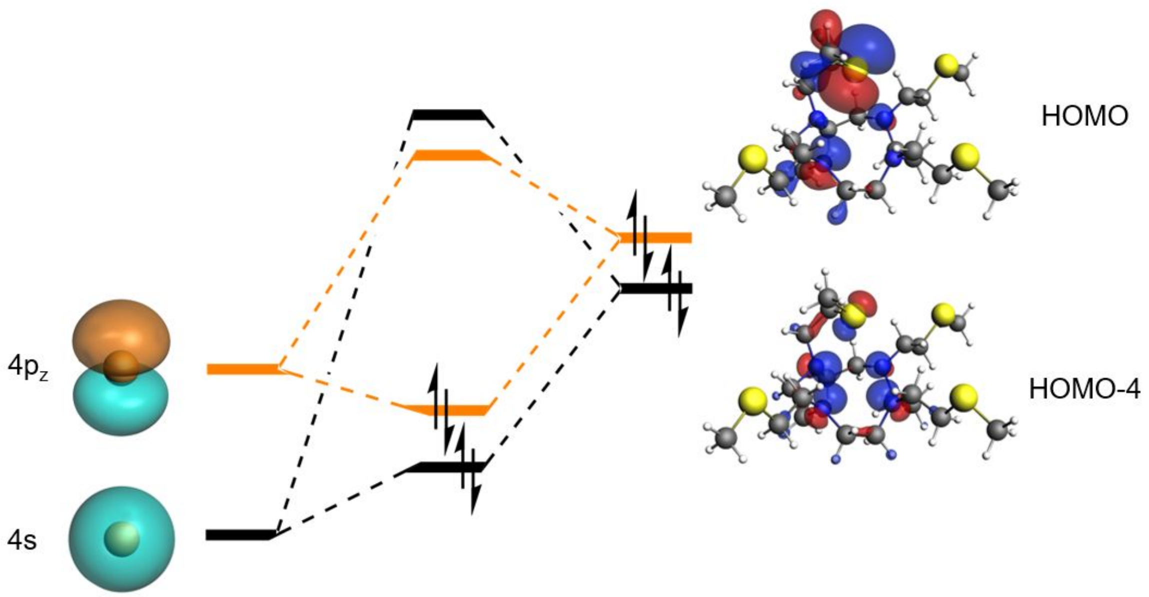


**Figure S34.** Molecular orbital diagram showing the two main bonding mode of the $Cu^+$-DO4S complex displaying a [4N,S] coordination mode. On the left, symmetry-adapted fragment orbitals (SFOs) representing the 4*s* and the 4$p_z$ orbitals located on the metal center. On the right, ligand SFOs involved in the primary (black) and secondary (orange) molecular bonding and antibonding orbital.

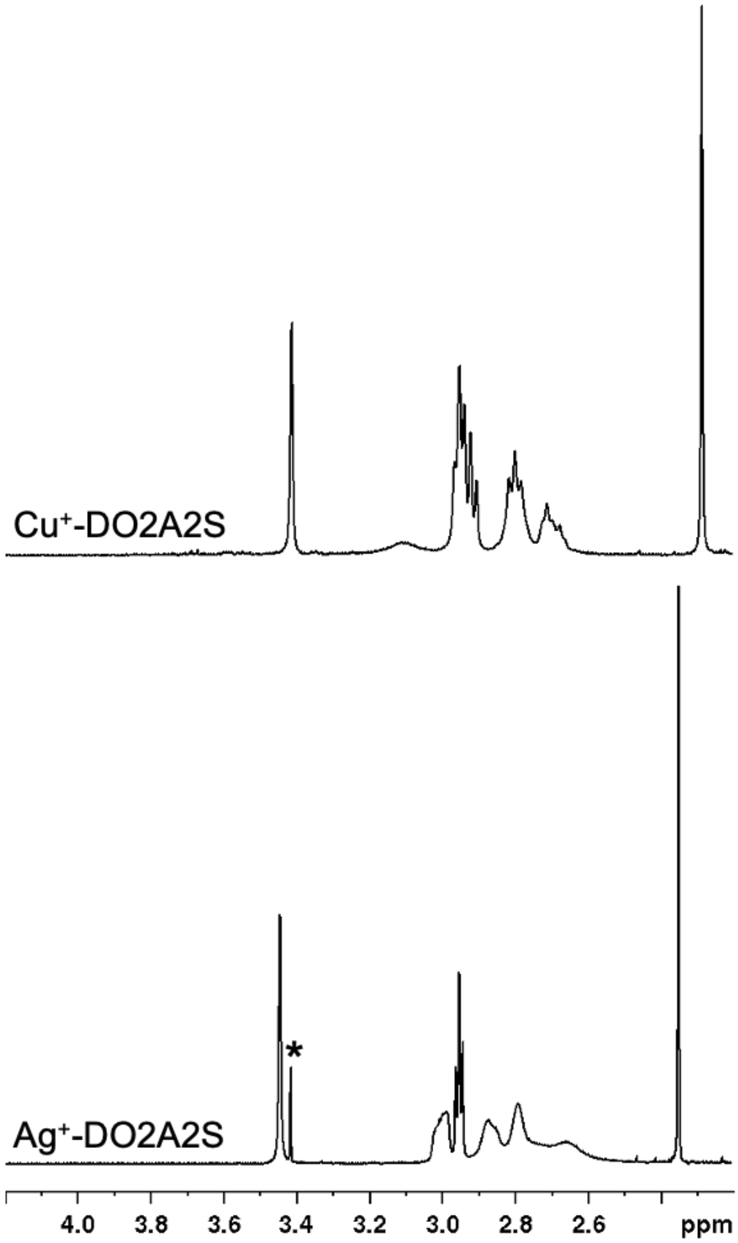


**Figure S35.** Comparison between the $^1$H-NMR spectra of $Cu^+$-DO2A2S (400 MHz, RT, $H_2O$ + 10% $D_2O$) (data from **Figure 9**) and $Ag^+$-DO2A2S (600 MHz, RT, $D_2O$) at pH 7. Data for the latter were taken from our previous work.[9] The signal marked with an asterisk is related to methanol impurity.

**Determination of the stability constants of the $Cu^+$ complexes from voltammetric data**

The relationship between the stability constants of the $Cu^{2+}$ and $Cu^+$ complexes with the ligand L was obtained using the following thermodynamic cycle:

| | | | | | | | |
|---|---|---|---|---|---|---|---|
| 1) | $Cu^{2+}$ | + L | ⇋ | $CuL^{2+}$ | $\Delta G_1^0$ | = | $-RT\ln(\beta_{II})$ |
| 2) | $CuL^{2+}$ | + $e^-$ | ⇋ | $CuL^+$ | $\Delta G_2^0$ | = | $-nF\,E_2^0$ |
| 3) | $Cu^{2+}$ | + $e^-$ | ⇋ | $Cu^+$ | $\Delta G_3^0$ | = | $-nF\,E_3^0$ |
| 4) | $Cu^+$ | + L | ⇋ | $CuL^+$ | $\Delta G_4^0$ | = | $-RT\ln(\beta_I)$ |

where $n = 1$, $\beta_{II}$ and $\beta_I$ represent the formation constants of $CuL^{2+}$ and $CuL^+$, respectively, and $E°_2$ is the standard potential for the unbound $Cu^{2+}/Cu^+$ redox couple. It was assumed that the experimental $E_{1/2}$ values approximate the standard potentials ($E°_3$).
The stability constants for the $Cu^+$ complexes were obtained from:

$$\Delta G_4^0 = \Delta G_1^0 + \Delta G_2^0 - \Delta G_3^0 \tag{6}$$

so that:

$$\ln(\beta_I) = \ln(\beta_{II}) + \frac{nF}{RT}(E_2^0 - E_3^0) \tag{7}$$

# Supplementary references

(1) Tosato, M.; Verona, M.; Doro, R.; Dalla Tiezza, M.; Orian, L.; Andrighetto, A.; Pastore, P.; Marzaro, G.; Di Marco, V. Toward Novel Sulphur-Containing Derivatives of Tetraazacyclododecane: Synthesis, Acid–Base Properties, Spectroscopic Characterization, DFT Calculations, and Cadmium(II) Complex Formation in Aqueous Solution. *New J. Chem.* **2020**, *44* (20), 8337–8350.

(2) Pniok, M.; Kubíček, V.; Havlíčková, J.; Kotek, J.; Sabatie-Gogovà, A.; Plutnar, J.; Huclier-Markai, S.; Hermann, P. Thermodynamic and Kinetic Study of Scandium(III) Complexes of DTPA and DOTA: A Step Toward Scandium Radiopharmaceuticals. *Chem. Eur. J.* **2014**, *20* (26), 7944–7955.

(3) Di Marco, V.; Pastore, P.; Tosato, M.; Andrighetto, A.; Borgna, F.; Realdon, N. pH-Static Titrations for Kinetic Studies of Metal-Ligand Complex Formation: The Case Example of the Reaction between Strontium(II) and DOTA. *Inorganica Chim. Acta* **2019**, *498*, 119147.

(4) Leugger, A. P.; Hertli, L.; Kaden, T. A. Metal Complexes with Macrocyclic Ligands. Ring Size Effect on the Complexation Rates with Transition Metal Ions. *Helv. Chim. Acta* **1978**, *61* (7), 2296.

(5) Styka, M. C.; Smierciak, R. C.; Blinn, E. L.; DeSimone, R. E.; Passariello, J. V. Copper(II) Complexes Containing a 12-Membered Macrocyclic Ligand. *Inorg. Chem.* **1978**, *17* (1), 82–86.

(6) Lacerda, S.; Campello, M. P.; Santos, I. C.; Santos, I.; Delgado, R. Study of the Cyclen Derivative 2-[1,4,7,10-Tetraazacyclododecan-1-yl]-Ethanethiol and Its Complexation Behaviour towards d-Transition Metal Ions. *Polyhedron* **2007**, *26* (14), 3763–3773.

(7) Ševčik, R.; Vanek, J.; Lubal, P.; Kotková, Z.; Kotek, J.; Hermann, P. Formation and Dissociation Kinetics of Copper(II) Complexes with Tetraphosphorus Acid DOTA Analogs. *Polyhedron* **2014**, *67*, 449–455.

(8) Riesen, A.; Zehnder, M.; Kaden, T. A. Metal Complexes of Macrocyclic Ligands. Part XXIII. Synthesis, Properties, and Structures of Mononuclear Complexes with 12- and 14-membered Tetraazamacrocycle-*N*,*N*′,*N*″,*N*‴-tetraacetic Acids. *Helv. Chim. Acta* **1986**, *69* (8), 2067–2073.

(9) Tosato, M.; Asti, M.; Dalla Tiezza, M.; Orian, L.; Häussinger, D.; Vogel, R.; Köster, U.; Jensen, M.; Andrighetto, A.; Pastore, P.; Di Marco, V. Highly Stable Silver(I) Complexes with Cyclen-Based Ligands Bearing Sulfide Arms: A Step Toward Silver-111 Labeled Radiopharmaceuticals. *Inorg. Chem.* **2020**, *59* (15), 10907–10919.
